# Druglike Molecular Degraders of the Oncogenic RNA-Binding Protein HuR

**DOI:** 10.1021/jacsau.5c00551

**Published:** 2025-07-16

**Authors:** Liann Kassabri, Raphael I. Benhamou

**Affiliations:** The Institute for Drug Research of the School of Pharmacy, Faculty of Medicine, 26742 The Hebrew University of Jerusalem, Jerusalem 91121, Israel

**Keywords:** RNA-Binding Protein, bifunctionnal molecules, biphasic degradation, HuR, breast cancer, 3D cancer models, therapeutic, degraders, molecular glues, PROTAC

## Abstract

Hu antigen R (HuR), also known as ELAVL1, is an RNA-binding protein (RBP) that plays a pivotal role in promoting oncogene expression by stabilizing oncogenic mRNAs. Elevated cytoplasmic levels of HuR are strongly associated with critical processes in breast cancer progression, including enhanced proliferation, survival, and metastasis. Targeting HuR presents a promising therapeutic strategy for aggressive subtypes of breast cancer. In this study, we developed small molecule degraders using both Molecular glues and Proteolysis TArgeting Chimera (PROTAC) technologies. The most effective degraders significantly reduced HuR levels in breast cancer cell lines, exhibiting a biphasic degradation profile due to dual-pocket engagement. This resulted in decreased expression of HuR-associated mRNAs and inhibition of breast cancer cellular phenotypes in both 2D and 3D spheroid cancer models. The lead degrader met all druglikeness criteria across the evaluated models. With the increasing interest in molecular glues and PROTACs within pharmaceutical development, targeted protein degradation is emerging as a powerful strategy for addressing previously undruggable proteins. These findings underscore the potential of molecular glues and PROTACs to navigate the challenges of targeting structurally dynamic RBPs such as HuR. The development of these degraders offers a promising therapeutic pathway with significant implications for cancer and other RNA-driven diseases.

## Graphical abstract

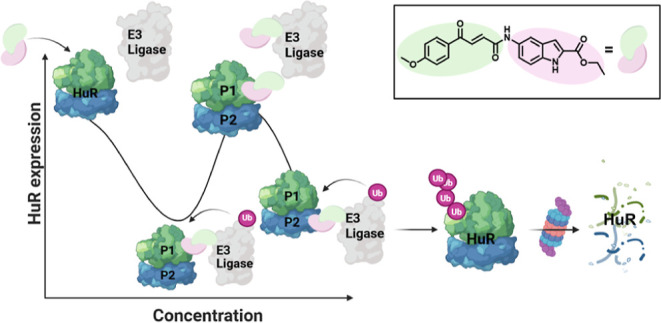

## Introduction

RNA-binding proteins (RBPs) are trans-acting factors that regulate all aspects of the RNA life cycle, including transcription, splicing, modification, translation, and metabolism.^1–4^ Due to their critical role, the dysregulation of RBPs contributes to the development of numerous diseases, positioning them as potential therapeutic targets.^2,5^ Hu antigen R (HuR), also known as ELAVL1, is an RBP that binds to adenine- and uridine-rich elements located in the 3′-untranslated region (UTR) of mRNA.^6,7^ By binding to these regions, HuR protects mRNA from exonuclease-mediated degradation and prevents rapid deadenylation, thereby increasing mRNA stability and enhancing steady-state levels.^6–8^ Accumulation of HuR in the cytoplasm and its overexpression have been observed in a wide range of cancer tissues.^9,10^ In breast cancer, HuR stabilizes transcription factors, growth factors, cytokines, and mRNAs associated with proto-oncogenes—including *B-cell leukemia/lymphoma 2* (*Bcl2*) and *forkhead box Q1* (*FOXQ1*)—by binding to their UTRs (Figure 1A).^10–13^ Consequently, HuR’s overexpression is linked to poor distant disease-free survival and serves as a prognostic factor for unfavorable clinical outcomes in breast cancer.^10,12^ Taken together, these findings underscore HuR as a compelling therapeutic target. However, due to its conformational plasticity and the equilibrium between different conformations,^14^ designing effective HuR inhibitors remains a challenge. Utilizing fragment-based drug discovery and structure–activity relationship analysis, Wu et al. identified small molecules that directly bind to the RNA-binding pocket of HuR, disrupting its interaction with target mRNAs.^10,15,16^ Nonetheless, these molecules do not completely eliminate HuR’s activity, allowing residual function that may still stabilize oncogenic mRNAs. Additionally, feedback mechanisms can lead to increased HuR expression, diminishing long-term efficacy. Dose-dependency and potential off-target effects further constrain the therapeutic window and elevate toxicity risks.^15,17^ Therefore, alternative strategies should be explored to efficiently degrade HuR.^18^

**Figure 1:**
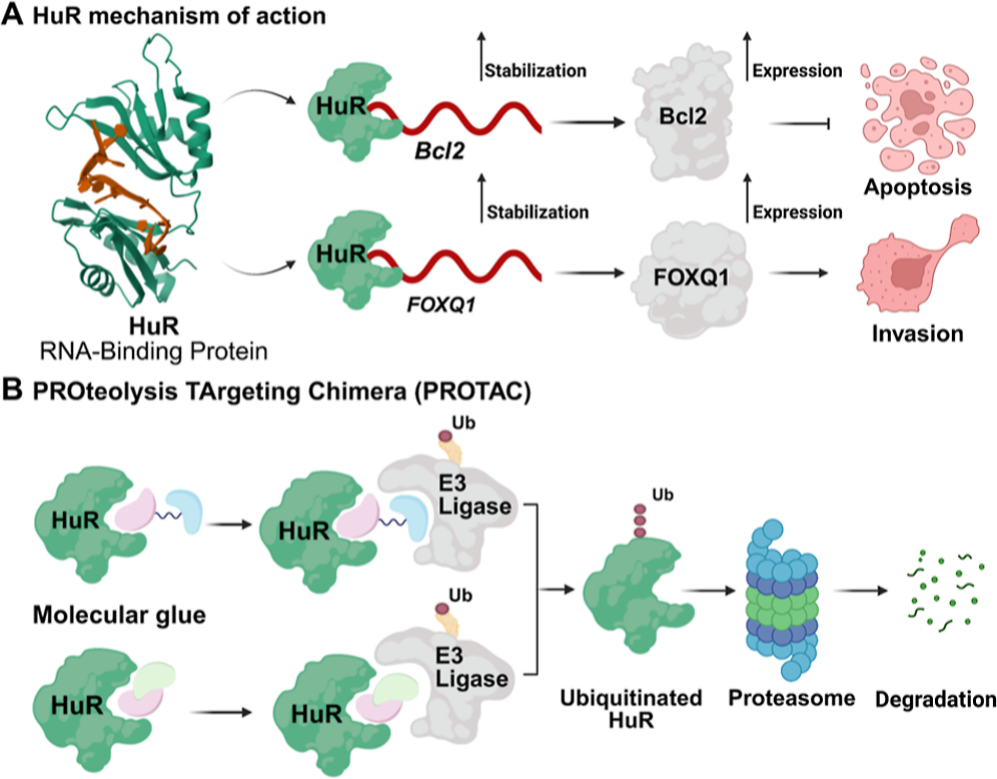
(A) HuR‘s role in breast cancer progression. (B) HuR targeted degradation strategies: PROTAC and molecular glues mechanisms of action.

Targeted protein degradation (TPD) has emerged as a significant approach for the degradation of traditionally undruggable proteins of interest (POIs).^19–21^ Proteolysis TArgeting Chimeras (PROTACs) and monovalent molecular glue degraders represent two principal strategies within TPD. PROTACs are heterobifunctional molecules comprised of a POI ligand, a linker, and a ligand that recruits an E3 ubiquitin ligase.^22^ The chemical composition and length of the linker are crucial factors that influence a PROTAC’s capacity to bridge the POI and the E3 ligase. By simultaneously binding to both the POI and the E3 ligase, a ternary complex is formed, facilitating the proximity of the E3 ligase to the POI. This interaction promotes the ubiquitination of the POI, marking it for degradation by the proteasome (Figure 1B).^21–24^

Several recruiters have been identified for the E3 ligase cereblon (CRBN) which have been leveraged for PROTAC TPD.^25–27^ While each CRBN recruiter offers distinct advantages, thalidomide-based ligands are considered a superior choice. This preference is attributed to their high efficacy, availability as small molecule ligands, well-defined structure that facilitates ease of synthesis and chemical tunability, and clinical validation in advanced-phase studies.^25,28,29^

PROTACs exhibit a bell-shaped dose–response curve, known as the hook effect, where efficacy decreases at high concentrations.^30^ This phenomenon occurs because excess PROTAC molecules can saturate binding sites on either the POI or the E3 ligase, leading to the formation of unproductive binary complexes instead of the essential ternary complex.^30^ This poses several challenges, particularly in dosing, as achieving optimal efficacy requires maintaining PROTAC concentration within a narrow therapeutic window to avoid both under-dosing and the hook effect.^31,32^ Furthermore, at high concentrations, degradation efficiency declines significantly, ultimately limiting the extent of target protein knockdown.^31^

In contrast, molecular glue degraders are small molecules that share key features such as inducing protein–protein interactions, promoting E3 ligase–substrate complex formation, and enabling targeted protein degradation with favorable drug-like properties due to their low molecular weight (<500 Da) and lack of linkers. They can be divided into two classes: monovalent glues, which bind a single protein (usually the E3 ligase) and reshape its surface to recruit a neo-substrate (e.g., thalidomide derivatives acting on CRBN),^33,34^ and bivalent glues, which engage both the E3 ligase and substrate through distinct binding sites on a compact scaffold, without necessitating a linker.^35,36^ Recent works from Nomura’s lab and Ciulli’s lab have shown that such bivalent, linkerless glues can drive degradation of proteins without the need for traditional PROTAC architecture.^36–39^ This dual engagement mechanism, combined with their small size, enhances cellular uptake and broadens the chemical space of degraders. These advances are expanding the utility of molecular glues as an emerging therapeutic strategy. Due to their dual-binding nature, these compounds may exhibit a hook effect—a feature more typical of PROTACs^30^ —distinguishing them mechanistically from classical monovalent molecular glues, yet supporting their classification as a unique, functionally distinct subclass within the broader molecular glue paradigm.

For PROTACs and molecular glues to serve as effective therapeutic agents, they should ideally exhibit druglike properties such as low molecular weight, high membrane permeability, and favorable oral bioavailability.^40^ These characteristics are crucial for efficient cellular uptake, systemic distribution, and target engagement. Molecular glues generally possess sizes under 500 Da and better comply with *Lipinski’s Rule of Five*, enhancing their potential for oral bioavailability and making them more suitable for central nervous system applications compared to larger, less permeable PROTACs.^35,41,42^ Additionally, molecular glues align with other druglikeness guidelines, including the (i) GSK criteria: prioritizing the avoidance of reactive and toxic groups, and (ii) Pfizer properties: optimizing lipophilicity (log *P* values) to balance solubility and minimize off-target effects.^41,43,44^ In contrast, PROTACs often struggle to meet Lipinski’s criteria due to their larger size and bifunctional design, which can result in reduced membrane permeability and suboptimal pharmacokinetics. Pfizer’s druglikeness criteria have expanded the acceptable size range, having been developed to accommodate the emergence of novel hybrid drugs with unique characteristics advancing into clinical phases.^41,44,45^ In this research, we developed a series of PROTACs and molecular glues or the targeted degradation of the RNA-binding protein HuR. Notably, several compounds from both classes significantly decrease HuR levels and influence the cancer-associated phenotype in breast cancer cells. The lead degrader fully satisfies druglikeness criteria across all evaluated models, positioning it as a promising candidate for therapeutic development against breast cancer pathology.

## Results and Discussion

### PROTAC and Molecular Glues Design for Targeted Degradation

The synthesis of various PROTACs and molecular glues targeting HuR was conducted with the objective of optimizing degradation efficiency and selectivity. Building on the HuR ligand scaffold introduced by Wu et al.,^15^ we synthesized a series of HuR ligand derivatives featuring indole and benzothiophene cores, along with ester and carboxylic acid functional groups. Subsequently, to convert the HuR ligands into degraders, we conjugated the derivatives to thalidomide, a CRBN recruiter, using various linkers, which can be broadly categorized into two main groups: triazole and heterocyclic linkers. For the heterocyclic linkers, connection of a HuR ligand to thalidomide was facilitated using either an aniline or phenol moiety in a coupling reaction (Scheme 1a and Figure S1). In contrast, triazole linkers were formed by connecting the two ligands via a click chemistry reaction (Scheme 1b and Figure S1). As previously mentioned, molecular glues offer a novel and promising approach to induce TPD due to their low molecular weight and favorable drug-like properties. As a second strategy for HuR-targeted degradation, we synthesized five distinct molecular glues by coupling various HuR ligand derivatives with two different fumarate derivatives: a trifluoromethylphenyl cinnamamide derivative and the other a methoxyphenyl fumarate derivative (Scheme 2 and Figure S1).

**Scheme 1:**
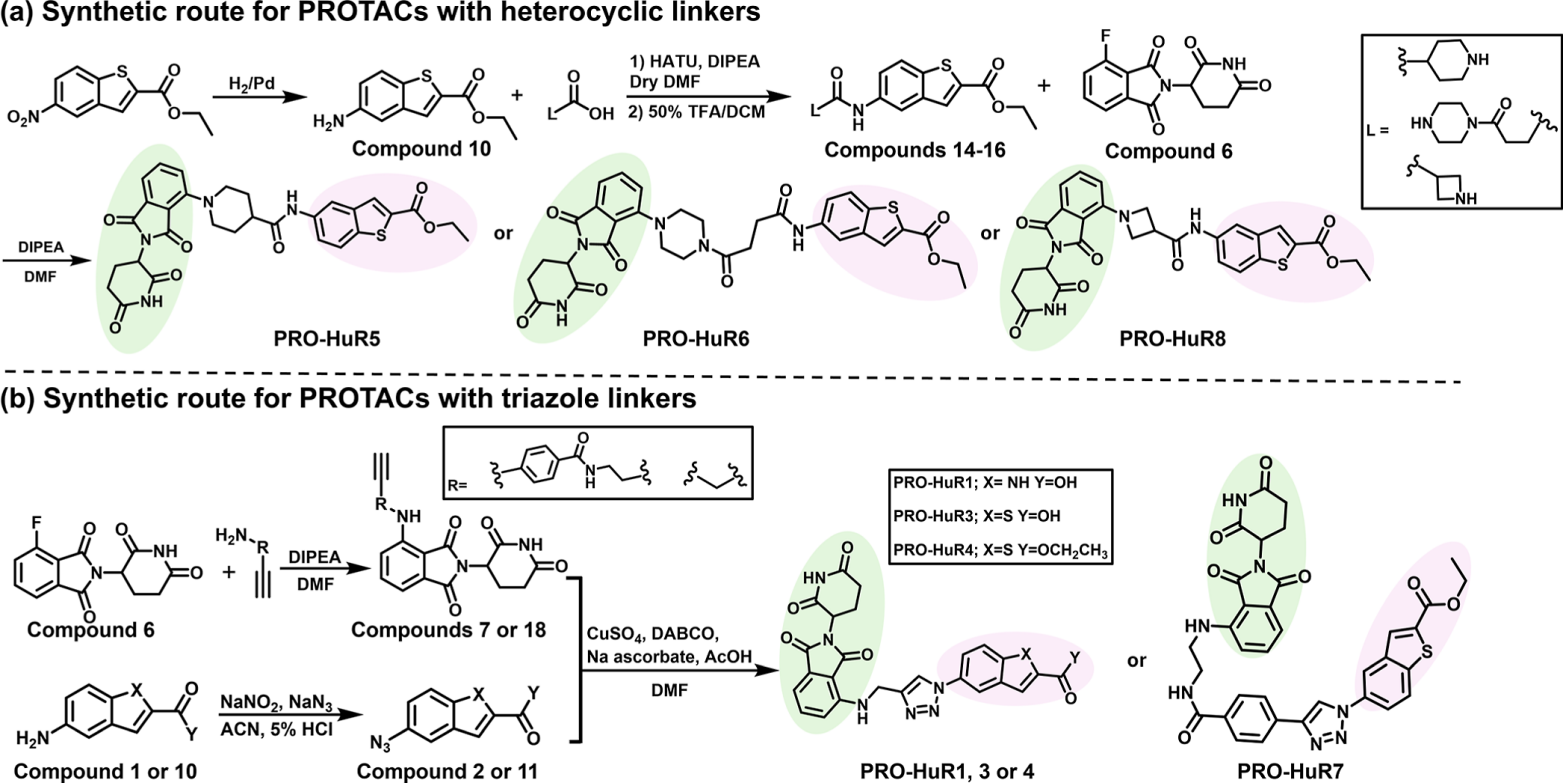
Synthetic Routes for PROTAC Synthesis^a^

a Green represents the E3 ligase ligand and pink represents the HuR ligand.

**Scheme 2:**
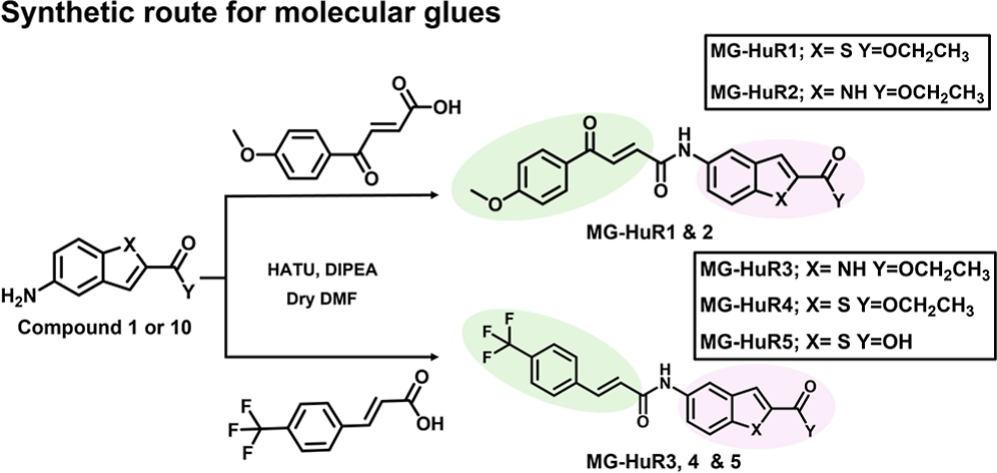
Synthetic Routes for Molecular Glues^a^

a Green represents the E3 ligase ligand and pink represents the HuR ligand.

These molecular glues are capable of recruiting RNF126, a RING E3 ubiquitin ligase and a critical component of the ubiquitin-proteasome system, to facilitate the targeted degradation of HuR.^36^

### Druglike Properties

Evaluation of physiochemical properties and druglike profiling were conducted for all synthesized compounds utilizing the SWISSADMET platform.^46^ This platform generates a plot for each compound by considering six physicochemical parameters: lipophilicity, size, polarity, solubility, flexibility, and saturation. The highlighted area on the plot indicates the physicochemical range for each parameter as defined by Lovering et al.^47^ and Ritchie et al.,^48^ defining the overall range in which a molecule should fall to be classified as druglike (Figure S2).

Among all synthesized compounds, the molecular glue derivatives predominantly fell within this range, while most of the PROTACs did not. Assessments of druglikeness based on these criteria were conducted using the ADMETLab3.0 web server.^49^ Notably, using this software all PROTAC derivatives synthesized in this study were classified as druglike according to Pfizer’s criteria (Table S2A). MG-HuR2, from the molecular glue derivatives, exhibited the most favorable druglike properties of all the compounds tested, consistent with Lipinski‘s, Pfizer‘s, and GSK’s rules—the only compound in the degrader series to meet all three criteria (Table S2B). These findings highlight MG-HuR2 as a particularly promising druglike HuR degrader.

### Fluorescence-Based Binding Evaluation

Following the synthesis, our initial aim was to ensure that the new derivatives retained their affinity for HuR’s RNA-binding pocket. Thus, a binding evaluation assay was conducted. As previously reported, the RNA-binding pocket of HuR specifically recognizes the UTR sequences of mRNAs, with UAUUUAUUUA identified as a common binding motif.^50^ To evaluate the binding of each degrader, we conducted a fluorescence-based assay utilizing a 5-carboxyfluorescein (5′ FAM)-labeled reporter. This fluorescence polarization (FP) assay is based on the displacement of the 5′ FAM-labeled mRNA from HuR’s RNA-binding pocket, which provides insight into both the binding affinity of the degrader and its capacity to disrupt the HuR-mRNA interaction, thereby serving as a means of validating target engagement. When the 5′ FAM-RNA probe is bound to HuR, it forms a binary complex, resulting in slower rotational movement and a consequently high FP signal. However, when either molecule displaces the 5′ FAM-labeled RNA, the probe is released into the solution, where it undergoes rapid rotation, leading to a significant reduction in the FP signal (Figure S3). This decrease in FP serves as a quantitative measure of the degrader’s ability to compete with mRNA for HuR binding. Importantly, all of the derivatives retained the ability to bind to HuR and to disrupt the complex between HuR and the labeled RNA. As mentioned above, we synthesized a series of HuR ligand derivatives featuring indole and benzothiophene cores, along with ester and carboxylic acid functional groups. Among these, the indole-based analogs demonstrated lower dissociation constant (*K*_d_) values in fluorescence binding assays, indicating a stronger binding affinity (Tables 1 and 2). However, no conclusive results were obtained to determine whether the ester or carboxylic acid functional group was superior in terms of potency or affinity. We observed that six PROTACs (PRO-HuR1-5 and PRO-HuR7) (Table 1 and Figure S4A) and two molecular glues (MG-HuR2 and MG-HuR3) (Table 2 and Figure S4B) exhibited *K*_d_ values lower than 0.5 μM. These results indicate strong binding affinities and suggest their potential for further development as HuR-targeted degraders.

**Table 1:**
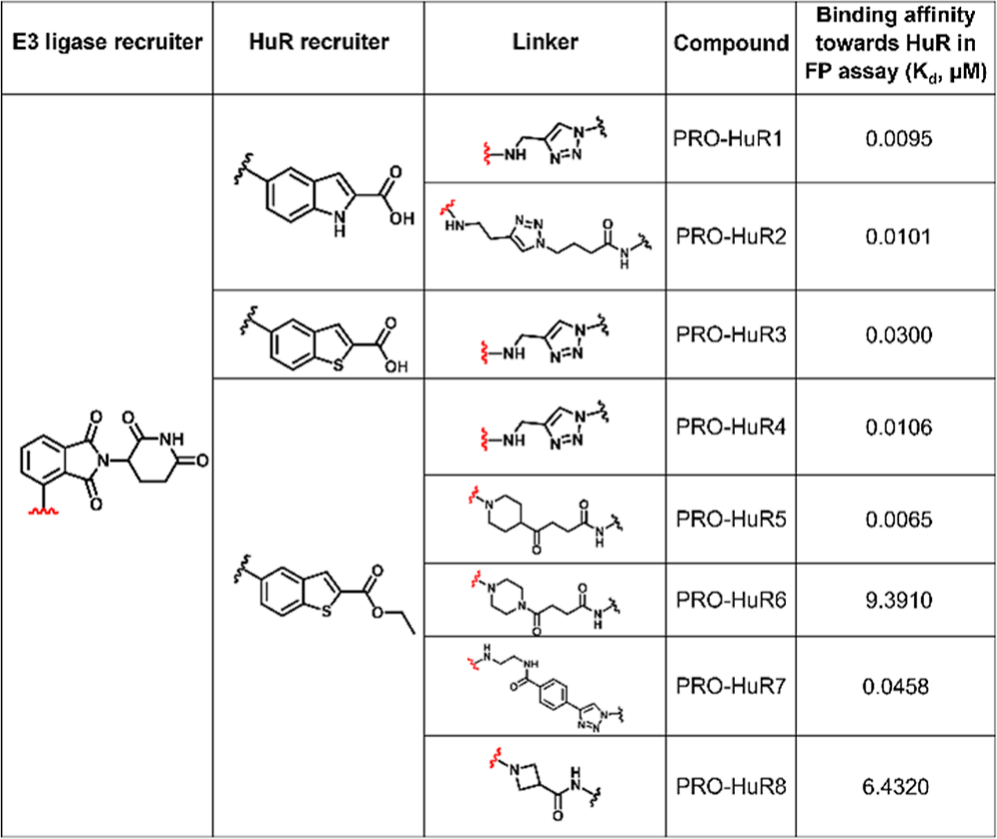
PROTAC Binding Affinities for HuR

**Table 2:**
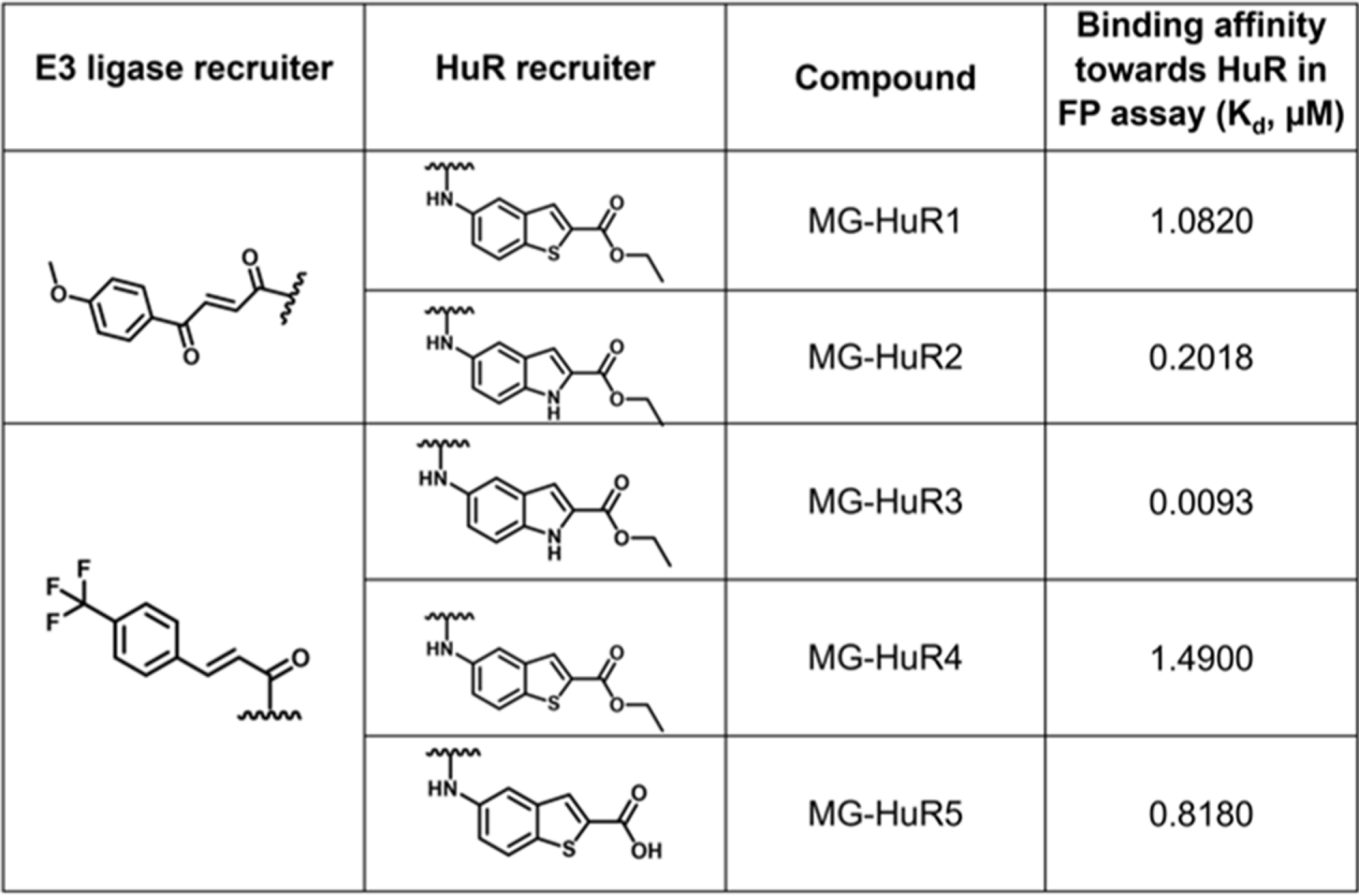
Molecular Glues Binding Affinities for HuR

### GFP Reporter Assay

To assess the effects of the HuR degraders in cells in a high-throughput fashion, a GFP-HuR reporter assay was utilized. In this assay, HuR was fused with green fluorescent protein (GFP), facilitating the monitoring of its expression and stability through fluorescence intensity (Figure 2A). A decrease in the GFP signal indicates HuR degradation. Human embryonic kidney (HEK) 293 cells were transfected with the GFP-HuR plasmid to enable the expression of the fusion protein, and the HuR degraders were administered at a single concentration of 10 μM for 24 h. Among the compounds evaluated, PRO-HuR3-5 and MG-HuR2 & 5 demonstrated a significant reduction in fluorescence intensity (Figure 2B,C), indicating successful cellular uptake and effective HuR degradation. These compounds were thus selected for further evaluation.

**Figure 2:**
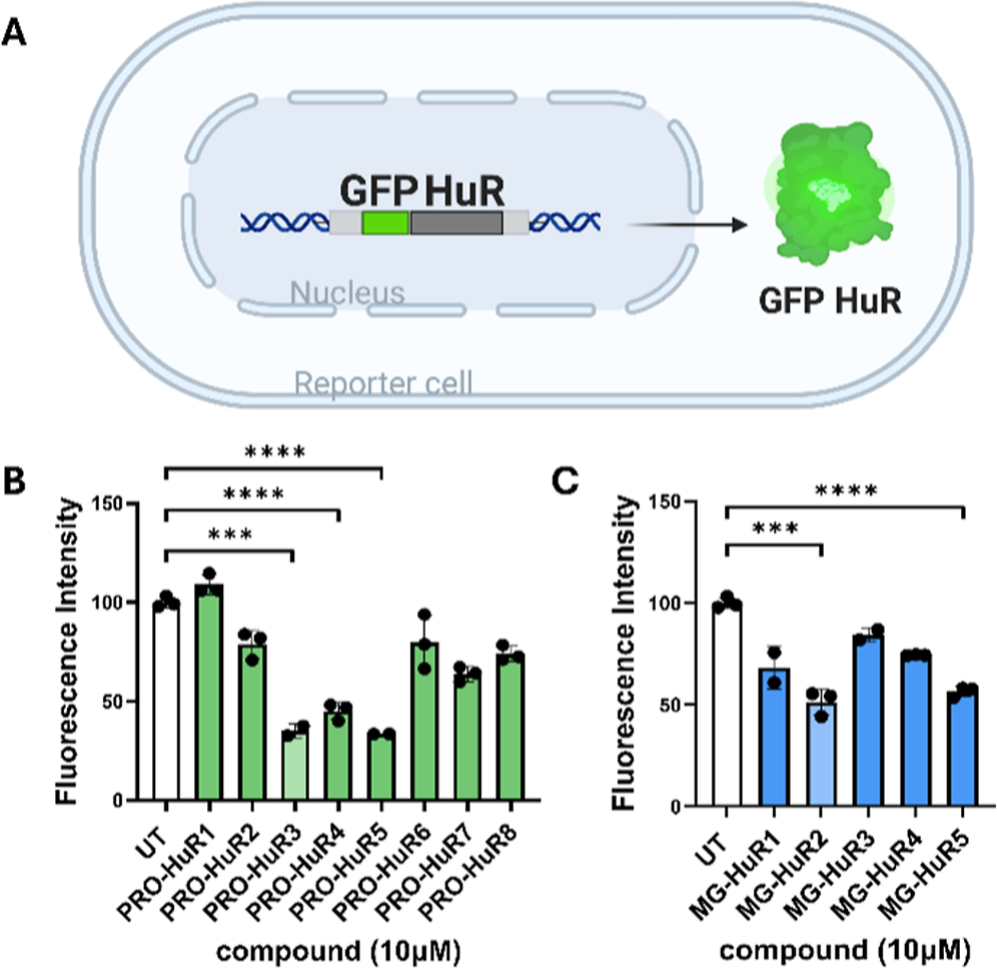
HuR cellular targeted degradation using a fluorogenic reporter. (A) Schematic representation of a reporter cell expressing GFP-tagged HuR. (B) Single-dose experiment for all PROTACs at 10 μM for 24 h. (C) Single-dose experiment for all molecular glues at 10 μM for 24 h. Data is presented as the mean ± SD (*n* = 3), *** represents *p* ≤ 0.001, **** represents *p* ≤ 0.0001 as determined by unpaired *t*-test comparison relative to UT.

### HuR Protein Expression

The next phase of the study focused on evaluating the degradation capabilities of the most promising degraders in breast cancer cell lines. Michigan Cancer Foundation-7 (MCF-7) cells were chosen due to their documented overexpression of HuR, which has been linked to cancer pathogenicity in this specific cell line.^10,12,13^ Consequently, reducing HuR expression to physiologically normal levels is considered a potential strategy for novel anticancer therapies targeting breast cancers associated with HuR overexpression.^51^ To assess the efficacy of the degraders, MCF-7 cells were treated with each compound at a 10 μM concentration for 24 h (Figure S5). Notably, the chemical composition and length of the PROTAC linkers influenced the degradation. Specifically, PRO-HuR4-8 share the same HuR binder scaffold, differing only in their linker structures. PROTACs with bulkier linkers exhibited poor degradation abilities, while PRO-HuR4 demonstrated higher degradation efficiency, indicating that a shorter linker is more favorable for facilitating the formation of the ternary complex (Figure S5B). This underscores the importance of linker optimization in the design of effective HuR-targeting degraders. Compounds that effectively reduced HuR expression, demonstrated high binding affinity in the FP assay, and exhibited a decrease in HuR expression in the GFP reporter assay were selected for further investigation. Specifically, PRO-HuR3, PRO-HuR4, and MG-HuR2 were then tested in a time-dependent manner at a concentration of 10 μM. MG-HuR2 and PRO-HuR3 showed a time-dependent response, whereas PRO-HuR4 did not (Figures 3A and 4A and S6), positioning them as the most promising degrader candidates from all of the PROTACs and molecular glues synthesized. For MG-HuR2, the onset of activity occurs at 24 h, followed by a decrease at 48 h, which may be due to its intracellular degradation. In contrast, PRO-HuR3 appears to have a longer onset time, with activity observed after 48 h. We expect that PRO-HuR3 will also eventually decrease in activity due to its intracellular degradation.

**Figure 3:**
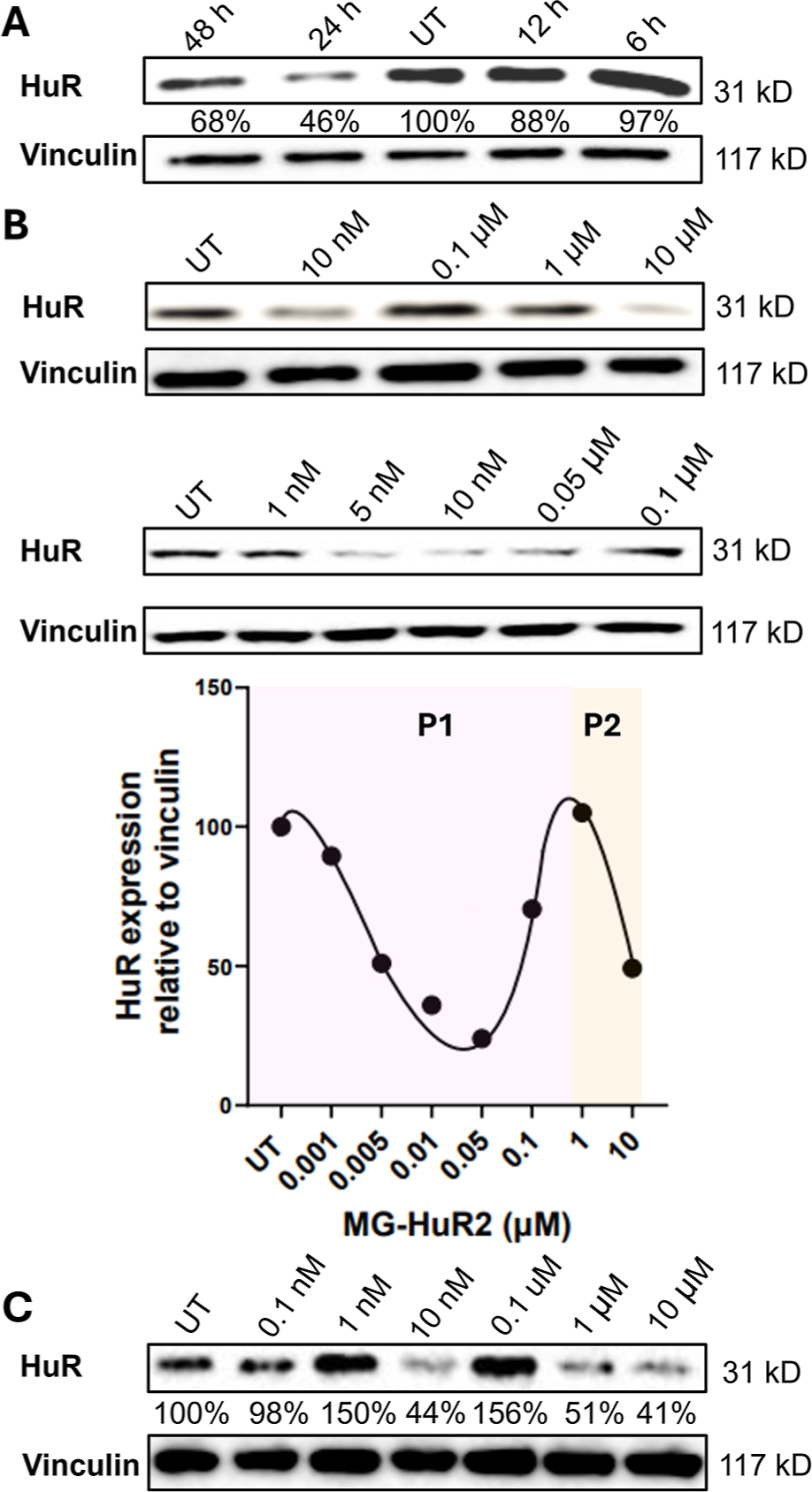
Evaluation of HuR protein degradation by MG-HuR2. (A) Time-dependent treatment of MG-HuR2 in MCF-7 cells, 10 μM dose. (B) Dose-dependent analysis and quantification of MG-HuR2 in MCF-7 cells. (C) Dose-dependent analysis of MG-HuR2 in MDA-MB-231 cells.

**Figure 4:**
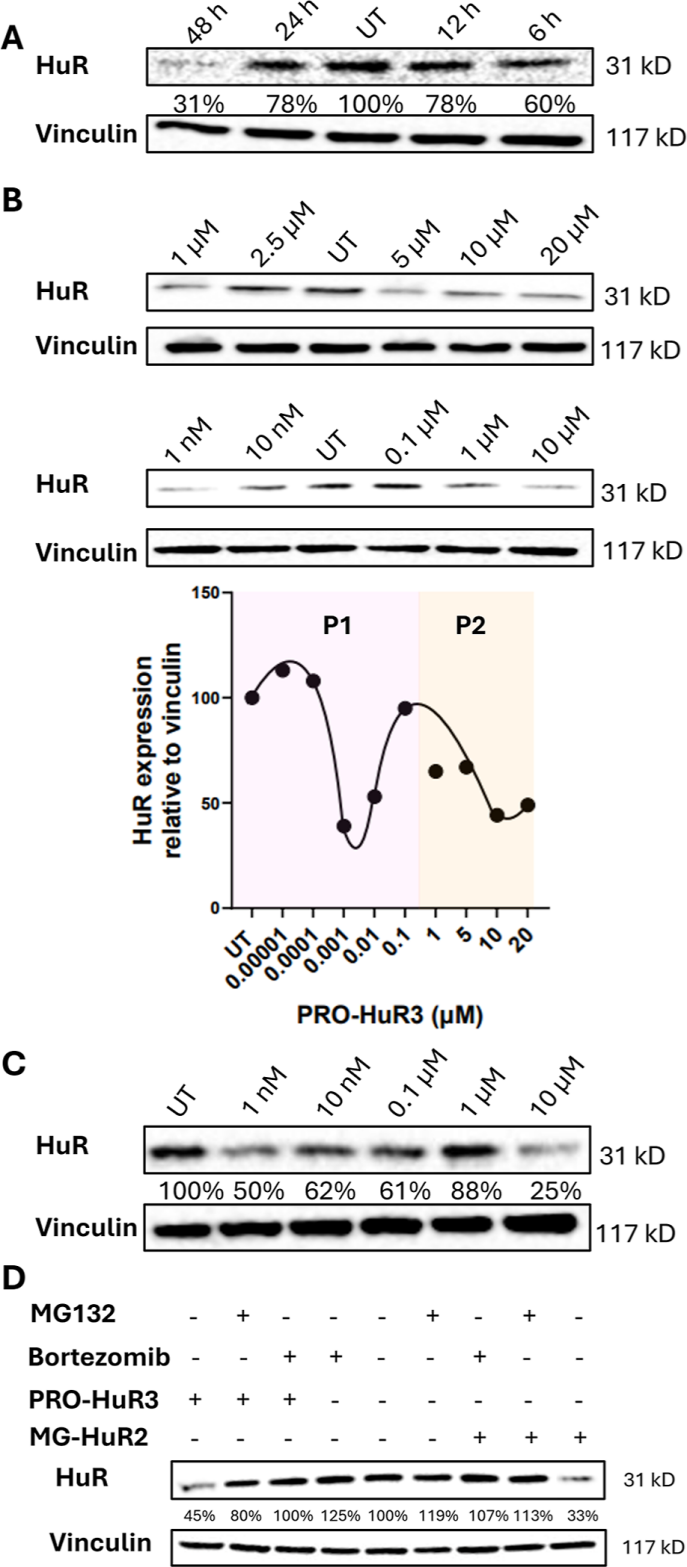
Evaluation of HuR protein degradation by PRO-HuR3. (A) Time-dependent treatment of PRO-HuR3 in MCF-7 cells, 10 μM dose. (B) Dose-dependent analysis and quantification of PRO-HuR3 in MCF-7 cells. (C) Dose-dependent analysis of PRO-HuR3 in MDA-MB-231 cells. (D) Mechanistic validation of HuR degradation in MCF-7 cells by treatment with proteasome inhibitors bortezomib and MG132.

Based on the time-dependent results, both MG-HuR2 and PRO-HuR3 were used to treat MCF-7 and MDA-MB-231 cells (metastatic differentiated adenocarcinoma of the breast 231, also known to overexpress HuR) for 24 and 48 h, respectively, across a concentration range of 10 nM to 10 μM. At a 10 μM dose of MG-HuR2, significant reductions in HuR levels were detected in both MCF-7 and MDA-MB-231 cells, with decreases of 80% and 59%, respectively. At lower degrader concentrations, lower activities were noted. Notably, an interesting pattern was observed at 10 nM; a significant decrease in HuR was again observed (85% in MCF-7 and 56% in MDA-MB-231; Figure 3B,C). To better understand this reduction at lower concentrations, we refined the dose–response analysis between 1 nM and 0.1 μM. Interestingly, within this range, a pronounced hook effect was observed, with significant reductions (∼70–80%) at 5 nM, 10 nM, and 50 nM.

To confirm that this phenomenon was not cell line-specific, the experiment was repeated in MDA-MB-231 cells, yielding a similar pattern (Figure 3C). Additionally, the most potent PROTAC derivative, PRO-HuR3, was evaluated in these cell lines for 48 h. Surprisingly, the hook effect was also observed in both MCF-7 and MDA-MB-231 cells treated with PRO-HuR3 (Figures 4B,C and S7). As with MG-HuR2, HuR expression was reduced at both 10 nM (42% in MCF-7 and 38% in MDA-MB-231) and at 10 μM (∼75% in both MCF-7 and MDA-MB-231), with a loss of degrader activity noted in between at around 0.1 μM. These results led us to identify a singular and intriguing biphasic hook effect for the most potent PROTAC and molecular glue derivatives.

To confirm that the degradation is attributable to activation of the proteolytic mechanism, MCF-7 cells were treated with 10 μM of either bortezomib or MG132, both reversible protease inhibitors.^50,51^ by inhibiting the 26S proteasome, the activity of MG-HuR2 and PRO-HuR3 was completely impaired (Figure 4D). For further validation of proteasome dependence, MCF-7 cells were treated with the ligands of HuR that compose MG-HuR2 and PRO-HuR3, referred to as Binder 1 and Binder 2, respectively (Figure S8). No significant decrease in HuR expression was observed, further confirming that the observed degradation is due to proteasomal activity rather than an inhibitory mechanism. Furthermore, to demonstrate that the observed effects stem from targeted degradation rather than nonspecific cytotoxicity resulting from E3 ligase recruitment, MCF-7 cells were treated in a dose-dependent manner with E3 ligase recruiters alone. Importantly, no decrease in HuR levels was observed (Figure S9). Based on these results, MG-HuR2 emerges as a promising new degrader of HuR, demonstrating a relatively fast onset of action and an enhanced ability to induce significant degradation at 10 nM concentrations.

### Docking Study

To elucidate the mechanism underlying the observed biphasic degradation pattern, we generated a docking model using the Schrödinger software. The biphasic hook effect can be attributed to the unique structural characteristic of RBPs, such as HuR, which can possess two distinct RNA-binding pockets.^15,50^ At lower concentrations, MG-HuR2 and PRO-HuR3 preferentially engage the P1 pocket, with molecular mechanics/generalized Born surface area (MM-GBSA) calculations indicating binding energies of −46.76 and −44.55 kcal/mol, respectively.^49^ This preferential binding correlates with the initial phase of degradation observed at low degrader concentrations (5 nM to 100 nM; Figures 3B and 4B and 5). At higher degrader concentrations (e.g., 10 μM), different complex engagement may occur at the P2 RNA-binding pocket. MM-GBSA calculations revealed binding energies of −39.04 and −41.06 kcal/mol for MG-HuR2 and PRO-HuR3, respectively.^52^ To experimentally validate the dual-pocket binding suggested by our docking data, we performed a fluorescence-based binding assay (Figure S10H). Unlike the previous displacement assay—which monitored competition between labeled RNA and HuR—we utilized the intrinsic fluorescence of the PROTAC derivative at a fixed concentration while titrating HuR protein. This approach allowed us to detect fluorescence changes corresponding to HuR binding events. Remarkably, the assay revealed two distinct IC_50_ values, indicating interaction at two independent sites. Fluorescence increased over two separate concentration ranges—0.0075–0.1 nM and 0.125–1 nM—with a clear dip between 0.1 and 0.125 nM. The subsequent increase suggests a second binding event distinct from the first. This biphasic response supports a model in which MG-HuR2 and PRO-HuR3 engage HuR via multiple binding modes—binding to site P1, P2, both, or neither—depending on the concentrations and relative availability of degrader and protein. The lower binding affinity of the P2 pocket relative to P1 likely facilitates the formation of a novel ternary complex at higher concentrations, resulting in the restoration of degradation activity despite the earlier hook effect (Figures 5 and S10A–D and Table S3).

**Figure 5:**
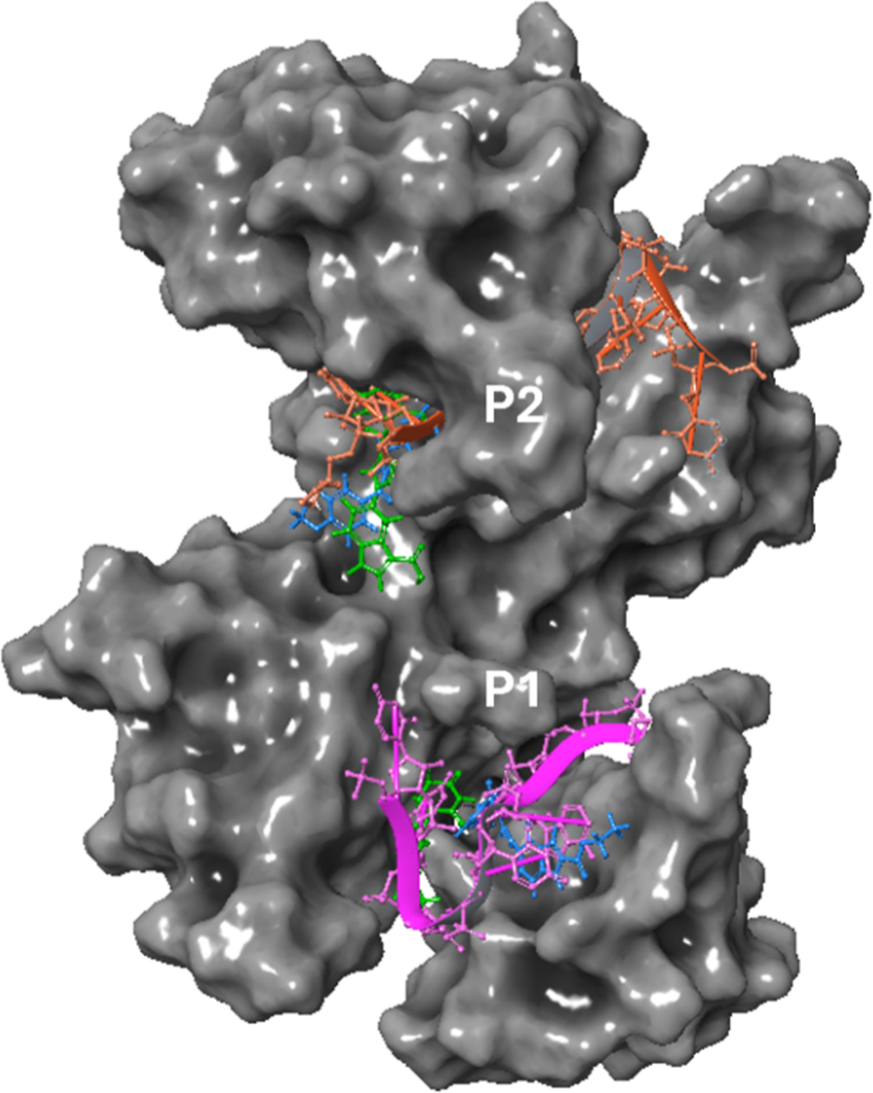
Dual binding modes of MG-HuR2 and PRO-HuR3 in the HuR-mRNA complex. 3D representation of HuR in complex with MG-HuR2 (blue) and PRO-HuR3 (green) in both RNA binding pockets, and mRNA in P1 and P2 (pink and orange, respectively), obtained after MM-GBSA docking analysis. Atomic coordinates were obtained from the PDB model 4ED5.

This dual-pocket binding mechanism may enable a more sustained and resilient degradation response, distinguishing HuR-targeting degraders from conventional PROTACs, which typically exhibit a pronounced hook effect. Supporting this, comparative docking studies with a commercially available HuR-targeting molecular glue showed that MG-HuR2 binds directly within and across both RNA-binding pockets with stronger MM-GBSA binding energies, whereas the commercial compound binds only near the P1 pocket with significantly lower binding energy (−26.8 kcal/mol) (Figure S10A–G and Table S3). By engaging both pockets, degraders effectively reduce HuR levels, leading to a more comprehensive loss of function with a larger therapeutic window. This mechanism underscores a unique advantage of ligands capable of binding two structurally similar pockets within the same protein, albeit with differing affinities. In the case of HuR, this phenomenon is facilitated by the high degree of similarity between its two RNA-binding domains.

### Effect on Downstream Pathways

To investigate the downstream effects of MG-HuR2 and PRO-HuR3, we focused on two genes that are upregulated by HuR overexpression: *Bcl2* and *FOXQ1*. *Bcl2* plays a crucial role in inhibiting apoptosis, while *FOXQ1* is primarily implicated in invasion processes.^53–55^ We first assessed the mRNA abundance of each in MCF-7 cells following treatment with either MG-HuR2, PRO-HuR3, Binder 1, or Binder 2. Our results indicated a decrease in the expression of both genes at concentrations ranging from 1 nM to 100 nM. Notably, in comparison with the HuR ligands, no effect was observed on the RNA levels of the tested genes at any of the concentrations evaluated, highlighting the strength of the targeted degradation approach (Figures 6A and S11 and S12). Interestingly, relative to the lower concentrations of both degraders an increase in *Bcl2* expression was noted at 1 μM for both degraders, which is consistent with the observed decline in degrader activity at this concentration due to the hook effect (Figure 6A). Additionally, Bcl2 protein expression was evaluated in both MCF-7 and MDA-MB-231 cells after treatment with either MG-HuR2 or PRO-HuR3 (Figures 6B and S13). The hook effect was again observed in Bcl2 protein expression, underscoring the functional link between HuR and Bcl2. Importantly, Bcl2 has long been regarded as a therapeutically attractive target for cancer treatment but remains challenging to drug due to the absence of a conventional active site amenable to small molecule binding.^56,57^ MG-HuR2 significantly reduced Bcl2 protein expression at both the RNA and protein levels in MCF-7 and MDA-MB-231 cells, achieving reductions of 88% and 45% at a concentration of 10 nM, respectively (Figures 6B and S13A). These results further underscore the potential of MG-HuR2 as a potential therapeutic compound in the treatment of breast cancer.

**Figure 6:**
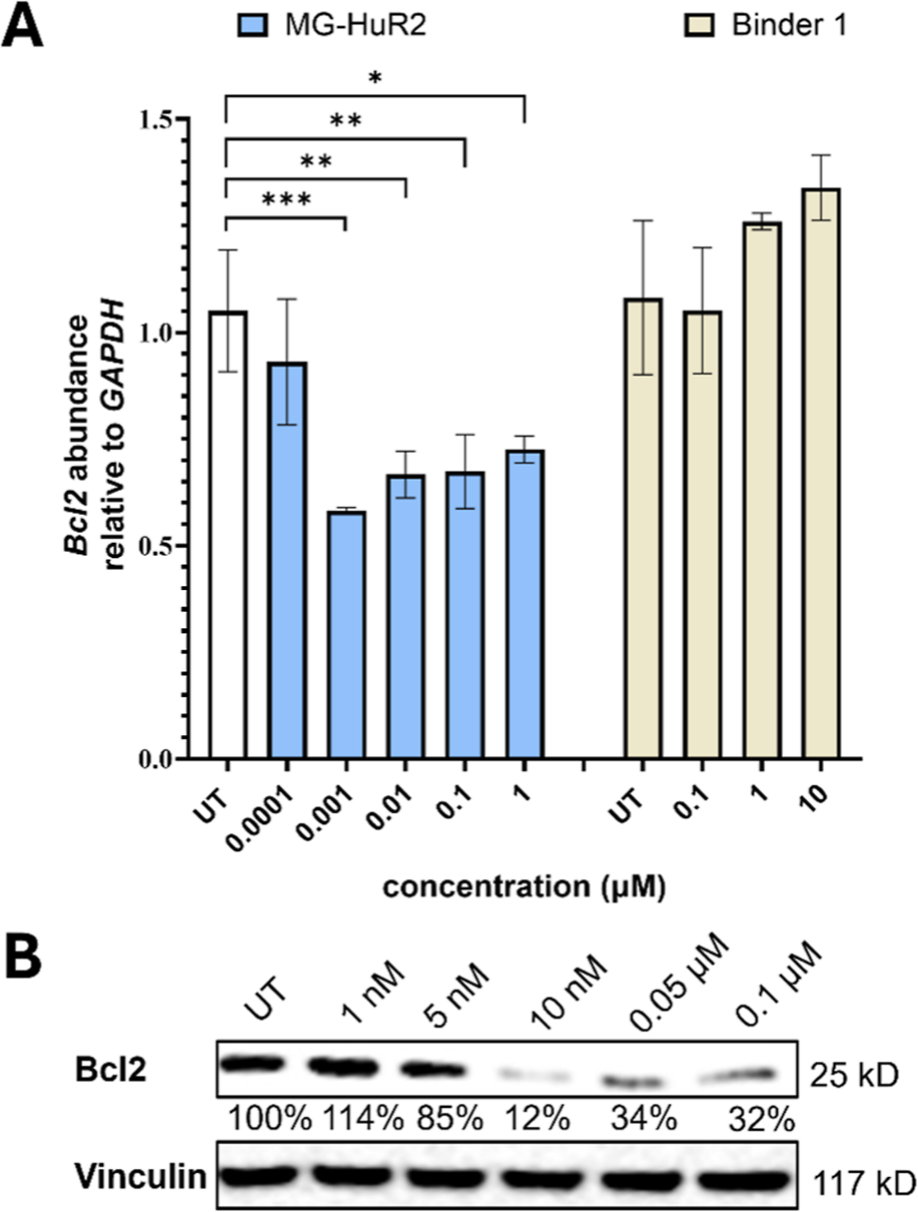
Evaluation of MG-HuR2 effect on downstream targets. (A) Abundance of *Bcl2* in MCF-7 cells treated dose-dependently with MG-HuR2 and Binder 1 for 24 h, measured by RT-qPCR. (B) Bcl2 expression in MCF-7 treated dose-dependently with MG-HuR2 for 24 h. Data is presented as the mean ± SD (*n* = 9), * represents *p* ≤ 0.05, ** represents *p* ≤ 0.01, *** represents *p* ≤ 0.001 as determined by a one-way ANOVA comparison relative to UT.

### Inhibition of Cancer Cellular Phenotypes

To further assess the cellular functional consequences of MG-HuR2 and PRO-HuR3 on cancer characteristics, proliferation, cytotoxicity, and apoptosis assays were performed using both breast cancer cell lines. First, cell viability was assessed after treatment with MG-HuR2 or Binder 1 at a 10 nM and 10 μM dose. Using crystal violet, the cell membrane, DNA, and proteins of viable adherent cells were stained, allowing for the evaluation of cytotoxicity.^58,59^ MG-HuR2 exhibited cytotoxic activity against the MCF-7 cell line, unlike the binder control and E3 ligase recruiters (Figures 7A and S14A), highlighting the impact of degradation on cancer cell viability. To assess the specificity and safety of MG-HuR2, we also tested its effect on MCF-10A, a nontumorigenic mammary epithelial cell line. No significant cytotoxicity or change in viability was observed at either 10 nM or 10 μM concentrations, indicating a favorable selectivity profile (Figure S14B,C). Furthermore, a significant decrease in cell proliferation was observed following treatment with either MG-HuR2 or PRO-HuR3 compared to their Binders 1 and 2 at both 24 and 48 h, with a more pronounced reduction at 48 h (Figures 7B and S15 and S16). To elucidate the decrease in cell viability observed, we evaluated the potential increase in apoptosis using the fluorogenic Caspase-Glo 3/7 assay system. Upon cell lysis, active caspases cleave a luminogenic substrate, releasing aminoluciferin, which is subsequently converted to light by luciferase. The intensity of the signal directly correlates with the levels of apoptosis.^60^ Consistent with the reduction of the antiapoptotic protein Bcl2, a significant increase in apoptosis was observed at a 10 nM concentration of the degraders (Figures 7C and S17). Notably, phenotype inhibition was more pronounced after 48 h, which may be attributed to HuR degradation leading to destabilization of mRNAs such as *Bcl2* and *FOXQ1*. This destabilization reduces the levels of proteins essential for cancer cell survival, resulting in cumulative cellular stress, diminished proliferation, and enhanced cytotoxicity. At 10 nM, treatment with MG-HuR2 consistently decreased the evaluated cancer phenotypes—proliferation, cytotoxicity, and apoptosis—showing improvement of several orders of magnitude over Binder 1, the ligand of HuR that composes MG-HuR2. This enhanced effect is likely due to the catalytic nature of the molecular glue degraders, offering promising potential for cancer treatment.

**Figure 7:**
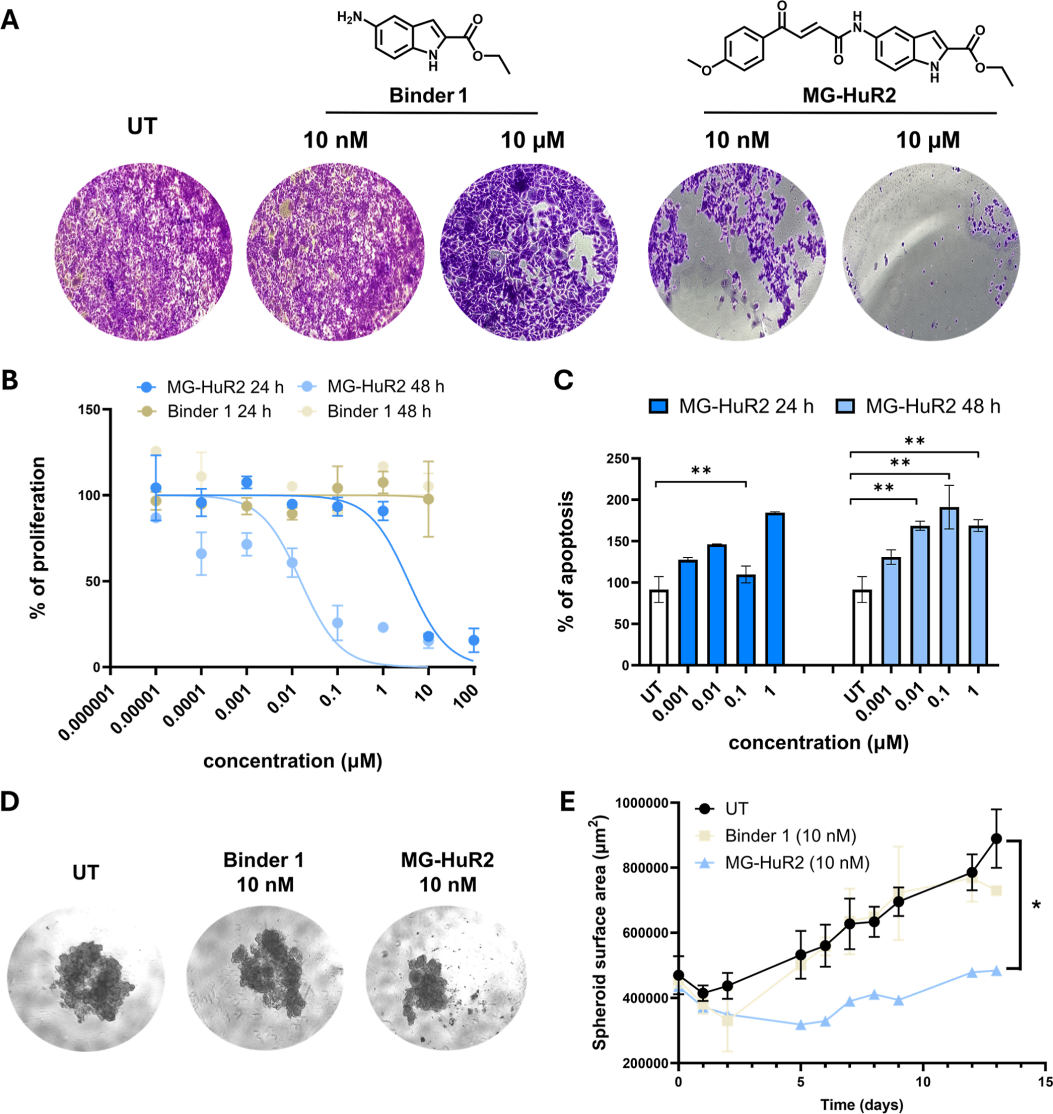
Cancer cellular phenotype evaluation. (A) Viability assay of MCF-7 cells treated with MG-HuR2 and Binder 1 for 48 h, measured using crystal violet. (B) Dose–response experiment for MG-HuR2 and Binder 1 for 24 and 48 h in MCF-7 cells. Data is presented as the mean ± SD (*n* = 3), curve was extrapolated via inhibitor vs normalized response-fitting using Prism 10.2.2. The experiments were performed in triplicate. (C) Dose–response experiment for MG-HuR2 for 24 and 48 h in MCF-7 cells. Data is presented as the mean ± SD (*n* = 3), * represents *p* ≤ 0.05, ** represents *p* ≤ 0.01 as determined by a one-way ANOVA comparison relative to UT. (D) Representative images for MCF-7 spheroid growth, day 12 after treatment. (E) MCF-7 spheroid surface area growth after treatment with a single dose of MG-HuR2 and Binder 1. Data is presented as the mean ± SD (*n* = 2). * represents *p* ≤ 0.05 as determined by a one-way ANOVA comparison relative to UT.

### Breast Cancer 3D Spheroid Model

To further assess MG-HuR2 and Binder 1 in a tumor microenvironment-mimetic system, we used 3D spheroids model of MDA-MB-231 and MCF-7. Over a 14 and 10-day treatment period, respectively, 10 nM of MG-HuR2 inhibited spheroid growth after 2 days with sustained effects throughout the duration of treatment. This resulted in markedly smaller spheroid surface areas in both cell lines (Figures 7D,E and S18). Notably, Binder 1 exhibited no significant impact on spheroid growth, with a decrease in surface area observed only at day 14 for MCF-7 and day 9 for MDA-MB-231, highlighting the potential difference of the degrader approach compared to the inhibition strategy. This progressive shrinkage suggests potent cytotoxic activity, likely mediated by apoptosis (supported by elevated caspase-3/7 activity, Figure 7C) and/or inhibition of proliferative signaling pathways targeted by the compounds (Figure 7B).

## Conclusion

The primary challenge in targeting HuR with small molecule inhibitors lies in its conformational plasticity. To address this, we designed a library of PROTAC, and molecular glues compounds based on the scaffold structure of known HuR inhibitors. Our preliminary screening identified three potential therapeutic degraders of HuR, among which MG-HuR2 and PRO-HuR3 were selected for in-depth biological evaluation. Both degraders demonstrated effective HuR reduction in breast cancer cell lines through a proteasome-dependent degradation mechanism. Unexpectedly, both compounds exhibited an unusual hook effect, with activity diminishing at intermediate concentrations but restoring at higher doses, which led us to identify a biphasic degradation profile. This unique kinetic pattern is attributed to the compound’s ability to engage two distinct binding pockets on HuR, facilitating enhanced ubiquitination and proteasomal degradation. This dual-pocket engagement contributes to a more efficient and sustained depletion of HuR, resulting in more complete functional silencing. Importantly, MG-HuR2 and PRO-HuR3 not only degraded HuR but also reduced the expression of HuR-associated mRNAs, thereby inhibiting key cancer-related phenotypes. Among the two most potent degraders, MG-HuR2 emerged as the most promising candidate, displaying potent activity at low nanomolar concentrations, significantly suppressing cancer cell proliferation, and enhancing both cytotoxicity and apoptosis. MG-HuR2 also showed enhanced potency in a 3D breast cancer model by reducing spheroid growth rate. Its compliance with essential druglikeness criteria further reinforces its therapeutic potential. These findings highlight the promise of RBPs as a strategy for cancer therapy. Notably, the molecular glue approach outperformed the PROTAC approach, potentially due to its superior druglike properties. This research paves the way for future studies exploring how RBP degradation can mitigate their pathological overexpression and contribute to novel cancer treatment strategies.

## Experimental Section

### Cell Lines

Compounds were tested in MDA-MB-231 human breast adenocarcinoma (HTB-26; ATCC), MCF-7 human breast adenocarcinoma (HTB-22; ATCC), HEK-293 (CRL-1573; ATCC) and MCF10A (CRL-10317; ATCC).

### Cell Culture

All cells were maintained at 37 °C with 5% CO_2_. MDA-MB-231 cells were cultured in RPMI 1640 medium with l-glutamine supplemented with 25 mM HEPES (Capricorn Scientific; HEP-B), 10% (v/v) FBS, and 1% (v/v) penicillin/streptomycin solution. MCF-7 and HEK-293 cells were cultured in DMEM medium with l-glutamine, 10% (v/v) fetal bovine serum (FBS; Sigma-Aldrich; F9665), and 1% (v/v) penicillin/streptomycin solution (Diagnovum; D910), and MCF-10A cells were cultured in DMEM/F12 medium supplemented with 5% (v/v) horse serum (Sigma-Aldrich; H1138), 20 ng/mL epidermal growth factor (EGF; Peprotech; AF-100-15-100), 0.5 μg/mL hydrocortisone (Sigma-Aldrich; H0888), 100 ng/mL cholera toxin (Sigma-Aldrich; C8052), 10 μg/mL insulin (Sigma-Aldrich; 91077C), and 1% (v/v) penicillin/streptomycin solution (Diagnovum; D910).

### Fluorescence Polarization Assays

As previously reported, the sequence of the fluorescently labeled (5′-FAM) RNA probe is UAUUUAUUUA.^50^ The assays were performed in PBS buffer (20 mM sodium phosphate, 150 mM NaCl, 1 mM dithiothreitol, 0.05% PLURONIC-127, 15% DMSO) at 293 K. Fluorescence was measured using the Synergy H1 Hybrid Multi-Mode Microplate Reader (Biotek, Agilent) with excitation and emission wavelengths set to 485 and 528 nm, respectively. Each well of a 384-well plate contained 20 nM of the 5′-FAM RNA probe and 500 nM of HuR RRM1/2. Compounds were tested at eight concentrations (0.1 nM–100 μM), with compound 10 serving as a positive control and PBS as the negative control. The protein-mRNA mixture was incubated for 1.5 h to allow for binding interactions. After this incubation, the compounds were added, and the mixture was incubated for an additional hour. The FP emission of the resulting HuR-compound complex was then recorded. The percent inhibition of binding was calculated by comparing the FP values of experimental conditions to the PBS control. The FP value corresponding to the HuR-RNA complex in the presence of DMSO was used as the 100% complexation reference, while the FP value of the labeled RNA alone was set as 0% complexation. The dissociation constant (*K*_d_) for the compound-HuR binding interaction was determined by fitting the dose–response curve to specific binding models using Prism 10.2.2. All experiments were performed in triplicate.

### GFP Reporter Assay

HEK-293 cells were cultured in 100 mm diameter plates to approximately 70% confluency and transfected with 15 μg of GFP-HuR (ELAVL1) reporter plasmid (Addgene #121162) in a 1:2 ratio with the PEI transfection reagent (Med Chem Express, HY-K2014) at a concentration of 1 mg/mL, following the manufacturer’s recommended protocol. After 6 h of transfection, the cells were reseeded into 96-well plates. After an additional 12 h of incubation, the cells were treated with either DMSO (vehicle, 0.1% (v/v)) or the synthesized compounds at a concentration of 10 μM for 24 h. Fluorescence measurements were taken using the Synergy H1 Hybrid Multi-Mode Microplate Reader (Biotek, Agilent) with excitation and emission wavelengths set to 479 and 520 nm, respectively, according to the manufacturer’s instructions. All experiments were performed in triplicate.

### Viability Assay

MCF-7 cells were seeded in 96-well plates to 80% confluency and treated with synthesized compounds at 10 nM for 24 h. After treatment, the medium was removed, and cells were gently washed with PBS to remove any residual media. Cells were then fixed by adding 100 μL of 100% cold methanol and incubating for 10 min. The methanol was aspirated, and the cells were allowed to air-dry fully. The cells were stained with 50 μL of 0.1% crystal violet for 10 min, after which excess crystal violet was aspirated and the cells were washed with PBS. To quantify cell viability, 100 μL of 33% acetic acid was added to each well to solubilize the bound dye. The plate was incubated for 40 min with gentle mixing. Fluorescence intensity was then measured at 590 nm using Synergy H1 Hybrid Multi-Mode Microplate Reader (Biotek, Agilent). Cell morphology and staining were visualized using a 4K HDMI Industrial Digital Camera at 75 fps. All experiments were performed in triplicate.

### Cell Counting-Proliferation Assay

MCF-7 and MDA-MB-231 cells were seeded in 96-well plates to 80% confluency and treated with synthesized compounds at eight concentrations (0.1 nM–100 μM) for 24 and 48 h. Cell proliferation was assessed using the Resazurin Assay Kit (Abcam, ab228554), where each sample was incubated with 1× WST-8/CCK8 solution for 1 h. After incubation, fluorescence was measured by absorbance at 460 nm using the Synergy H1 Hybrid Multi-Mode Microplate Reader (Biotek, Agilent). Cell proliferation was calculated as the percent decrease in fluorescence of treated cells compared to untreated controls. All experiments were performed in triplicate.

### Apoptosis Assay

MCF-7 cells were seeded in 96-well plates to 80% confluency and treated with synthesized compounds at four concentrations (1 nM–1 μM) for 24 and 48 h. Cell apoptosis was analyzed using the Caspase-Glo 3/7 Assay System (Promega, G8090). Each sample was incubated with Caspase-Glo 3/7 solution for 1 h, followed by luminescence measurement. Luminescence was measured at 255 nm using the Synergy H1 Hybrid Multi-Mode Microplate Reader (Biotek, Agilent). Cell apoptosis was calculated as the percent increase in luminescence of treated cells compared to untreated controls. All experiments were performed in triplicate.

### General Protocol for Western Blotting

MDA-MB-231 and MCF-7 cells were seeded in 6-well plates at approximately 60% confluency in complete growth medium and treated with synthesized compounds at the indicated concentrations or with vehicle for 24 h. Total protein was extracted using RIPA cell lysis buffer (BioPrep) containing a protease inhibitor, and protein concentration was measured using the BCA protein assay (Sigma-Aldrich) according to the manufacturer’s protocol. Approximately 30 μg of total protein were resolved on a 12% SDS-acrylamide gel and transferred to a PVDF membrane (Immobilon FL transfer membrane; Merck). The membrane was washed with 1× Tris-buffered saline (TBS) containing 0.1% (v/v) Tween-20 (TBST; Tris-base, pH 7.6, NaCl, and Tween-20) and blocked in 1× TBST containing 5% (w/v) BSA for 2 h at room temperature. The membrane was then incubated overnight at 4 °C with primary antibodies: a 1:1000 dilution of rabbit anti-ELAVL1/HuR (Cell Signaling Technology, #12582), a 1:1000 dilution of mouse anti-Bcl2 (Cell Signaling Technology, #15071), or a 1:10,000 dilution of rabbit anti-Vinculin (Cell Signaling Technology, #13901) in 1× TBST containing 5% (w/v) BSA. Following primary antibody incubation, the membrane was washed with 1× TBST and incubated with a 1:10,000 dilution of the appropriate horseradish-peroxidase-conjugated secondary antibody (antirabbit IgG: Cell Signaling, #7074 or antimouse IgG: Cell Signaling, #7076) at room temperature for 2 h. After washing three times with 1X TBST (10 min per wash), protein bands were visualized using Clear Band ECL Substrate (Gene Bio-Application) on the Azure C300 Imaging System. The fold change in the expression of the target proteins (HuR or Bcl2) was calculated by normalizing the band intensity to the Vinculin band intensity using ImageJ.

### Proteasome Inhibitor Treatment

MCF-7 cells were seeded in 6-well plates at approximately 60% confluency and incubated for 12 h. The cells were then treated with 1 μM of the proteasome inhibitors Bortezomib (Thermo Fisher Scientific, CAS: 179324-69-7) and MG132 (Aaron Chemicals, CAS: 1211877-36-9). After treatment, the synthesized compounds were added at a concentration of 10 μM, and the cells were incubated for 5 h.

### mRNA RT-qPCR

MCF-7 cells were seeded into 12-well plates (∼150,000 cells per well) at 60–70% confluency incubated for 12 h. The cells were then treated with different synthesized compounds at the indicated concentrations or with vehicle control for 24 and 48 h. After treatment, total RNA was extracted using the QuickRNA Miniprep Kit (Zymo Research) according to the manufacturer’s protocol, including DNase I treatment. Reverse transcription was performed on 1 μg of total RNA using the qScript cDNA Synthesis Kit (Quantabio) following the manufacturer’s instructions. Approximately 30 ng of cDNA were used for each qPCR reaction, which was carried out using the Luna Universal qPCR Master Mix (New England BioLabs, cat# E3010G) on a CFX Opus 384 Real-Time PCR System. Relative expression levels of Bcl-2 and FOXQ1 were calculated by normalizing to GAPDH expression using the ΔΔCt method.

### In Silico Studies

Using the ADMET variables, pharmaceutical efficiency of the potent compounds was determined. SwissADME^46^ and ADMETlab 3.0 ^50^ online tools were used for virtual screening, which predicted the pharmacokinetic characteristics and druglikeness of targeted candidates.

### Molecular Docking

Molecular docking was performed using Schrödinger’s Maestro 25.1v, following a structured and validated workflow. For ligand preparation, MG-HuR2 and PRO-HuR3 were sketched as 2D structures, converted to 3D conformations using Maestro’s *LigPrep* module, and energy-minimized with the OPLS3 force field to optimize geometry. During this step, tautomeric and ionization states were generated at pH 7.4 to account for physiological conditions, and ring conformations were sampled to ensure ligand flexibility. The protein preparation involved importing the HuR structure from the Protein Data Bank (PDB ID: 4ED5) and refining it using Maestro’s *Protein Preparation Wizard*. Hydrogens were added, bond orders were corrected, and the hydrogen-bonding network was optimized using PROPKA, which calculated p*K*_a_ values of residues and assigned protonation states at pH 7.4. A restrained minimization was performed to relax the structure while maintaining the experimental geometry. Crystallographic water molecules within 5 Å of the ligand or active site were retained to preserve potential key interactions, while all others were deleted. For receptor grid generation, a grid was created to include the entire protein surface for blind docking to identify all potential binding pockets. Subsequently, the grid was minimized to only cover the P2 binding pocket. Finally, Glide docking (integrated into Maestro) was used to predict binding modes. Docking results were analyzed through GlideScores, docking scores, and interaction patterns. Additionally, postdocking MM-GBSA rescoring was performed to calculate binding free energies and improve affinity predictions.

### Fluorescence Binding Assay

As part of the dual-binding evaluation, Pro-HuR3 fluorescence was first characterized across a concentration range (1–100 μM; 1, 2, 5, 10, 20, 50, and 100 μM). The assays were performed in PBS buffer (20 mM sodium phosphate, 150 mM NaCl, 1 mM dithiothreitol, 0.05% PLURONIC-127, 15% DMSO) at 293 K. Fluorescence spectra were recorded using the Synergy H1 Hybrid Multi-Mode Microplate Reader (Biotek, Agilent), with excitation and emission scans identifying optimal wavelengths of 405–415 nm (excitation) and 520 nm (emission), respectively. For the binding assay, a fixed concentration of Pro-HuR3 (100 μM) was incubated with increasing concentrations of HuR protein in the same buffer conditions. PBS alone was used as a negative control. The protein–Pro-HuR3 mixtures were incubated for 1.5 h to allow binding interactions to occur. Fluorescence intensity was recorded under the previously established excitation/emission parameters. All experiments were performed in triplicate.

### Spheroid Formation

MDA-MB-231 and MCF-7 breast cancer cells were cultured in standard tissue culture plates using RPMI or DMEM media supplemented with 10% fetal bovine serum (FBS) and 1% penicillin–streptomycin until they reached approximately 80% confluency. Cells were washed with PBS and dissociated using 0.25% Trypsin-EDTA, followed by neutralization with complete medium. Viability was assessed via trypan blue exclusion, ensuring >90% viable cells. For spheroid formation, cells were seeded into ultralow attachment 96-well plates at a density of 5000 cells/well in 200 μL of complete medium. Plates were centrifuged at 1000 rpm for 5 min to promote aggregation and incubated at 37 °C in a humidified 5% CO_2_ atmosphere. Spheroid formation was monitored daily using an inverted microscope. Media exchanges or treatment additions were performed by carefully aspirating 100 μL of medium from each well and replacing it with an equal volume of fresh or treatment-containing medium. Differences in spheroid characteristics between the two cell lines were noted: MCF-7 cells, being epithelial and E-cadherin positive, formed compact, uniform spheroids at lower seeding densities (1000–5000 cells/well) over 3–5 days, whereas MDA-MB-231 cells, which lack E-cadherin, required higher densities (5000–10,000 cells/well) and formed looser aggregates typically within 2–4 days. All experiments were performed in duplicates. Surface area was calculated using Image View’s multipoint connection function.

## Supplementary Material



## Notes

Figures S1–S15, Tables S1–S2, general chemistry in formation, synthetic procedures, compound characterizations, and ^1^H NMR, and Analytical HPLC (PDF)

## Abbreviations

HuR: Hu antigen R
RBPs: RNA binding proteins
PROTAC: proteolysis targeting chimera
UTR: 3′-untranslated region
Bcl2: B-cell leukemia/lymphoma 2
FOXQ1: forkhead box Q1
TPD: targeted protein degradation
POIs: proteins of interest
CRBN: cereblon
HEK 293: human embryonic kidney 293
MCF-7: Michigan Cancer Foundation-7
MDA-MB-231: metastatic differentiated adenocarcinoma of the breast 231
5′ FAM: 5-carboxyfluorescein
MM-GBSA: molecular mechanics/generalized Born surface area
