# Supplementary material for: Druglike Molecular Degraders of the Oncogenic RNA-Binding Protein HuR
Source: JACS Au. 2025 Jul 16;5(8):3879–91. doi: 10.1021/jacsau.5c00551 (PMC12381739; doi:10.1021/jacsau.5c00551)
Supplement: Supplementary file 1 [file au5c00551_si_001.pdf]

Drug-like Molecular Degraders of the Oncogenic RNA-Binding Protein-HuR

Liann Kassabri,[a] and Raphael I. Benhamou [a],*

[a] The Institute for Drug Research of the School of Pharmacy, Faculty of Medicine, The Hebrew University of Jerusalem, Jerusalem, Israel

*Corresponding author: Raphael I. Benhamou, Email: raphael.benhamou@mail.huji.ac.il

Content:

1. General chemistry methods and instruments
2. Abbreviations
3. Synthetic procedures
4. Figures and Tables
5. References

1. General methods and instrumentation

All reactions were carried out under argon atmosphere and using anhydrous solvents, unless otherwise noted. Reagents were purchased at the highest commercial quality and used without further purification. All chemicals, unless otherwise stated, were obtained from commercial sources. Reactions were stirred magnetically and monitored by thin-layer chromatography (TLC) performed on SiliCycle aluminum-backed silica gel plates (F-254) using UV light (254 nm/365 nm) as a visualizing agent. Compounds were purified using silica gel chromatography (Silica gel, Bio Lab, 60 Å). Additional purification was conducted with an Ultimate 3000 semi-preparative HPLC instrument (Thermo Scientific), which was composed of a VWD-3400rs Detector, HPG-3200BX Pump, and Fraction Collector F. The system was equipped with an HPLC Column Luna C18 (250 × 21.2 mm). Separations were conducted with a flow rate of 10 mL/min, using a gradient of 5-90% MeOH or ACN (+ 0.1% FA) in water (+ 0.1% FA) over 40 minutes, followed by 5 minutes at 95% MeOH or ACN (+ 0.1% FA). Analytical HPLC was performed on a Shimadzu Nexera UHPLC (LC-40). Analyses were conducted with a flow rate of 0.3 mL/min and a gradient of 2-70% ACN in water. Matrix-assisted laser desorption ionization time-of-flight (MALDI-TOF) mass spectrometry was performed on a BRUKER microflex LRF MALDI-TOF/TOF instrument using dihydroxybenzoic acid as the matrix. Spectra were acquired using Bruker Daltonics Flex Control 3.4 and analyzed using Bruker Daltonics Flex Analysis 3.4.

2. Abbreviations

ACN : acetonitrile, BSA : bovine serum albumin, DCM : dichloromethane, DIPEA : N,N-Diisopropylethylamine, DMF : N, N-dimethylformamide, DMSO : dimethylsulfoxide, DMEM: Dulbecco's Modified Eagle Medium EtAOc : ethyl acetate, FA : formic acid, FBS : fetal bovine serum, HATU : 1-[Bis(dimethylamino)methylene]-1H-1,2,3-triazolo[4,5-b]pyridinium3-oxide hexafluorophosphate,HEPES : 4-(2-hydroxyethyl)-1-piperazineethanesulfonic acid, HCl : hydrogen chloride, HPLC : high-performance liquid chromatography, MALDI-TOF : Matrix-assisted laser desorption ionization time-of-flight, MeOH : methanol, $MgSO_4$:magnesium sulfate, MS : mass spectrometry, PE: petroleum ether, PBS : phosphate buffered saline, RT-qPCR : real time quantitative polymerase chain reaction, RPMI : Roswell Park Memorial Institute formulation, TLC: Thin layer chromatography, TBS : tris buffered saline, TBST : tris buffered saline with 0.05% (v/v) Tween-20, TFA : trifluoroacetic acid, UT: Untreated, UV: Ultraviolet.

3. Synthetic procedures:

Building blocks:

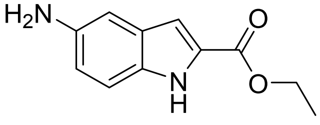

Compound 1. The compound synthesis was previously reported by David pierson P et al. [1] The procedure was, slightly modified. Briefly, 1H-Indole-2-carboxylic acid, 5-nitro-, ethyl ester (2 g, 8.5 mmol, 1 eq.) was dissolved in a 1:1 mixture of EtOAc and ethanol (5 mL). A spatula tip of Pd/C (10 wt%) was added. The reaction mixture was stirred under an atmosphere of H_2 for 3 hours. The reaction mixture was filtered through Celite, washed with EtOAc, and purified by column chromatography with a gradient up to 40% EtOAc in PE to obtain compound 1 (1.39 g, 79.7%). The characterization was done with MALDI-TOF; calculated MS: 204.23, and measured: 204.40.

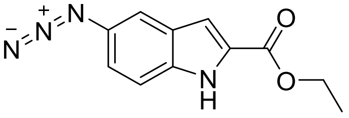

Compound 2. The compound synthesis was previously reported by Ambrose A et al.[2] The procedure was, slightly modified. Briefly, Compound 1 (0.5411 g, 2.65 mmol, 1.0 eq.) was dissolved in ACN, and 5% HCl aq (0.4 mL) was added and cooled to 0°C. An aqueous solution (0.02 mL) of $NaNO_2$ (0.22 g, 3.178 mmol, 1.2 eq.) was added dropwise to the solution. The reaction mixture was stirred at 0°C for 30 minutes. An aqueous solution (0.04 mL) of Na_3N (0.258 g, 3.974 mmol, 1.5 eq.) was added dropwise at 0°C. The resultant mixture was warmed to room temperature and stirred for 10 minutes. After completion, the reaction was diluted with EtOAc (20 mL) and water (10 mL).

After separation, the aqueous layer was extracted twice with EtOAc (20 mL). The combined organic layers were washed once with brine (10 mL), dried over $MgSO_4$, and evaporated under reduced pressure to obtain Compound 2 (0.245 g, 47%). The characterization was done with NMR; ^{1}H NMR (300 MHz, Chloroform-*d*) δ 9.11 (s, 1H), 7.40 (d, *J* = 8.8 Hz, 1H), 7.34 (d, *J* = 2.1 Hz, 1H), 7.16 (dd, *J* = 1.9, 0.9 Hz, 1H), 7.00 (dd, *J* = 8.8, 2.2 Hz, 1H), 4.42 (q, *J* = 7.1 Hz, 2H), 1.42 (t, *J* = 7.1 Hz, 3H).

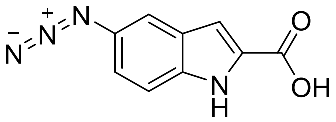

Compound 3. The compound synthesis was previously reported by Ambrose A et al.[2] The procedure was, slightly modified. Briefly, Compound 2 (0.0341 g, 0.148 mmol, 1.0 eq.) was dissolved in THF/ethanol/water (3:2:1 ratio), and NaOH (0.0176 g, 0.44 mmol, 3.0 eq.) was added and heated to 60°C overnight. THF and ethanol were removed, and the crude was acidified with 1 N HCl and dissolved in a minimal amount of water. The precipitate was then centrifuged (2500 rpm, 10 minutes, 4°C) and decanted. The remaining solid was then resuspended in water, centrifuged, and decanted again. Purified by UV-HPLC (5% - 90% MeOH in H_2O + 0.1% TFA, 40 min gradient) to obtain Compound 3 (0.0267 g, 89.2%). The characterization was done with MALDI-TOF; calculated MS: 202.17, and measured: 202.29.

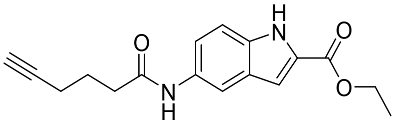

Compound 4. To a solution of 5-Hexynoic acid (0.065 g, 0.05 mL, 0.49 mmol, 1.2 eq.) in dry DMF (2 mL), DIEPA (0.3164 g, 0.42 mL, 2.44 mmol, 5 eq.) and HATU (0.28 g, 0.734 mmol, 1.5 eq.) were added and stirred for 10 minutes at room temperature. Then, Compound 1 (0.1 g, 0.489 mmol, 1 eq.) was added and stirred at room temperature for 16 hours. After completion, the reaction was diluted with EtOAc (20 mL) and water (10 mL). After separation, the aqueous layer was extracted twice with EtOAc (20 mL). The combined organic layers were washed once with brine (10 mL), dried over $MgSO_4$, and evaporated under reduced pressure. The crude product was purified using UV-HPLC (5% - 90% ACN in H_2O + 0.1% TFA, 50 min gradient) to obtain Compound 4 (0.1099 g, 75%). The characterization was done with MALDI-TOF; calculated MS: 298.34, and measured: 299.45. And used directly for the next step.

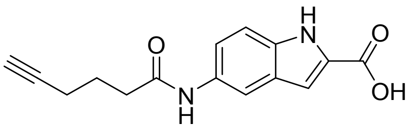

Compound 5. Compound 4 (0.043 g, 0.144 mmol, 1.0 eq.) was dissolved in THF/ethanol/water (3:2:1 ratio), and NaOH (0.0173 g, 0.432 mmol, 3.0 eq.) was added and heated to 60°C overnight. THF and ethanol were removed, and the crude was acidified with 1 N HCl and dissolved

in a minimal amount of water. The precipitate was then centrifuged (2500 rpm, 10 minutes, 4°C) and decanted. The remaining solid was then resuspended in water, centrifuged, and decanted again. Purified by UV-HPLC (5% - 90% MeOH in H_2O + 0.1% TFA, 40 min gradient) to obtain Compound 5 (0.0314 g, 80.6%). The characterization was done with NMR and MALDI-TOF; ^{1}H NMR (300 MHz, DMSO-d_6) δ 10.84 (s, 1H), 9.70 (s, 1H), 8.49 (s, 1H), 7.80 (d, *J* = 1.8 Hz, 1H), 7.24 (d, *J* = 8.7 Hz, 1H), 7.14 (dd, *J* = 8.7, 1.9 Hz, 1H), 6.53 (s, 1H), 2.81 (t, *J* = 2.6 Hz, 1H), 2.39 (t, *J* = 7.4 Hz, 2H), 2.22 (dt, *J* = 7.0, 3.5 Hz, 2H), 1.76 (p, *J* = 7.3 Hz, 2H). ^{13}C NMR (126 MHz, DMSO-d_6) δ 170.02, 166.18, 165.52, 139.15, 132.68, 131.14, 127.78, 115.70, 111.75, 111.38, 102.03, 84.36, 71.77, 35.26, 24.46, 17.65. Calculated MS: 270.29, and measured: 270.37.

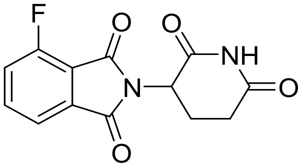

Compound 6. The compound synthesis was previously reported by Nowak R et al.[3] The procedure was, slightly modified. Briefly, 3-fluorophthalic anhydride (2 g, 12 mmol, 1 eq.) and 3-aminopiperidine-2,6-dione hydrochloride salt (2.2 g, 13 mmol, 1.1 eq.) were dissolved in AcOH (30 mL), followed by potassium acetate (3.54 g, 36 mmol, 3 eq.). The mixture was heated to 90°C overnight. The mixture was diluted with 150 mL of water and cooled over ice. The precipitate was then centrifuged (2500 rpm, 10 minutes, 4°C) and decanted. The remaining solid was then resuspended in water, centrifuged, and decanted again. The grey crude solid was adsorbed onto silica, and column chromatography was performed with a gradient up to 15% MeOH in DCM to obtain white solid Compound 7 (0.403 g, 62%). The characterization was done with MALDI-TOF; calculated MS: 276.22, and measured: 277.29.

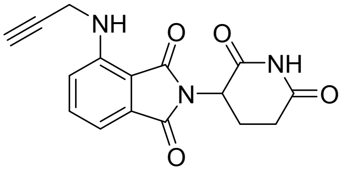

Compound 7. Compound 5 (0.3 g, 1.086 mmol, 1 eq.) was dissolved in DMF (10 mL), and DIPEA (0.421 g, 0.55 mL, 3.3 mmol, 3 eq.) was added. 2-Propynylamine (0.066 g, 77 µL, 1.21 mmol, 1.1 eq.) was dissolved separately in DMF (2 mL) and added to the reaction. The reaction mixture was heated to 90°C for 6 hours. The reaction was cooled to room temperature, diluted with EtOAc (40 mL) and water (20 mL). After separation, the aqueous layer was extracted twice with EtOAc (40 mL). The combined organic layers were washed once with brine (40 mL), dried over $MgSO_4$, and evaporated under reduced pressure. The crude product was purified using column chromatography with a gradient up to 5% MeOH in DCM and UV-HPLC (5% - 90% ACN in H_2O + 0.1% TFA, 60 min gradient) to obtain Compound 8 (0.318 g, 22.2%) as a yellow solid. The characterization was done with NMR and MALDI-TOF; ^{1}H NMR (300 MHz, DMSO-d_6) δ 11.13 (s, 1H), 7.95 (td, *J* = 7.9, 4.5 Hz, 1H), 7.80 (s, 1H), 7.77 (d, *J* = 3.6 Hz, 1H), 7.74 – 7.69 (m, 1H),

5.16 (dd, J = 12.9, 5.4 Hz, 1H), 2.92 (dd, J = 13.1, 4.4 Hz, 1H), 2.85 (t, J = 7.1 Hz, 1H), 2.63 (dd, J = 4.4, 2.4 Hz, 1H), 2.58 (d, J = 3.7 Hz, 1H), 2.54 (d, J = 4.5 Hz, 1H), 2.12 – 2.07 (m, 1H), 2.07 – 2.01 (m, 1H). ^{13}C NMR (75 MHz, DMSO-d_6) δ 173.02, 169.97, 166.41, 164.26, 158.84, 155.36, 138.40, 138.30, 133.75, 123.43, 120.36, 120.31, 117.41, 49.38, 31.20, 22.13. Calculated MS: 311.30, and measured: 312.14.

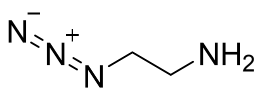

Compound 8. The compound synthesis was previously reported by Gnaccarini C et al.[4] The procedure was, slightly modified. Briefly, 2-(t-Butoxycarbonylamino)ethyl Bromide (2 g, 8.92 mmol, 1 eq.) was dissolved in DMF. Sodium azide (2 g, 31 mmol, 3.5 eq.) was dissolved separately and added. The reaction was stirred overnight at 110°C. The mixture was diluted with EtOAc (10 mL) and water (5 mL). After separation, the aqueous layer was extracted twice with EtOAc (10 mL). The combined organic layers were washed once with brine (10 mL), dried over $MgSO_4$, and evaporated under reduced pressure. For Boc deprotection, Compound 9-Boc (1 eq.) was dissolved in a 50% TFA solution in DCM (5 mL) and reacted for 30 min at room temperature. After completion, volatiles were evaporated under reduced pressure to obtain Compound 9 (0.65 g, 90%). The characterization was done with NMR; ^{1}H NMR (300 MHz, DMSO-d_6) δ 7.58 (s, 2H), 3.12 (s, 4H).

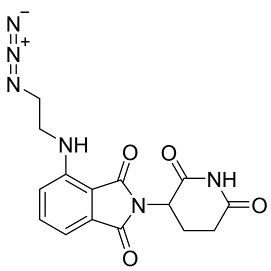

Compound 9. The compound synthesis was previously reported by Patil K et al.[5] The procedure was, slightly modified. Briefly, Compound 7 (0.05 g, 0.18 mmol, 1 eq.) was dissolved in DMF (10 mL), and DIPEA (69.78 g, 0.1 mL, 0.55 mmol, 3 eq.) was added. Compound 9 (0.017 g, 0.199 mmol, 1.1 eq.) was dissolved separately in DMF (2 mL) and added to the reaction. The reaction mixture was heated to 90°C for 6 hours. The reaction was cooled to room temperature, diluted with EtOAc (20 mL) and water (10 mL). After separation, the aqueous layer was extracted twice with EtOAc (20 mL). The combined organic layers were washed once with brine (20 mL), dried over $MgSO_4$, and evaporated under reduced pressure. The crude product was purified using column chromatography with a gradient up to 5% MeOH in DCM and UV-HPLC (5% - 90% MeOH in H_2O + 0.1% TFA, 45 min gradient) to obtain Compound 11 (0.1328 g, 55.5%). The characterization was done with MALDI-TOF; calculated MS: 342.31, and measured: 343.50.

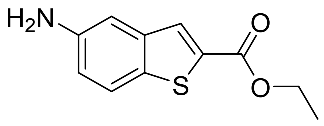

Compound 10. The compound synthesis was previously reported by Van Snick W et al.[6] The procedure was, slightly modified. Briefly, Ethyl 5-nitrobenzo[b]thiophene-2-carboxylate (1 g, 3.98 mmol, 1 eq.) was dissolved in a 1:1 mixture of EtOAc and ethanol (5 mL), and a spatula tip of Pd/C (10 wt%) was added. The reaction mixture was stirred under an atmosphere of H_2 for 4 hours. The reaction mixture was filtered through Celite, washed with EtOAc, and purified by column chromatography with a gradient up to 30% EtOAc in PE to obtain Compound 11 (0.838 g, 95.2%). The characterization was done with MALDI-TOF; calculated MS: 221.27, and measured: 221.18.

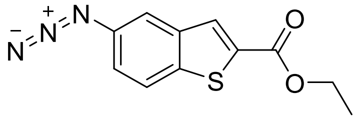

Compound 11. The compound synthesis was previously reported by Fieser L [7]. The procedure was adopted from Ambrose A et al.[2] and was slightly modified. Briefly, Compound 11 (0.2 g, 0.9 mmol, 1 eq.) was dissolved in ACN, and 5% HCl aq (0.4 mL) was added and cooled to 0°C. An aqueous solution (0.02 mL) of $NaNO_2$ (0.0758 g, 1.084 mmol, 1.2 eq.) was added dropwise to the solution. The reaction mixture was stirred at 0°C for 30 min. An aqueous solution (0.04 mL) of Na_3N (0.088 g, 1.356 mmol, 1.5 eq.) was added dropwise at 0°C. The resultant mixture was warmed to room temperature and stirred for 10 min. After completion, the reaction was diluted with EtOAc (20 mL) and water (10 mL). After separation, the aqueous layer was extracted twice with EtOAc (20 mL). The combined organic layers were washed once with brine (10 mL), dried over $MgSO_4$, and evaporated under reduced pressure to obtain Compound 12 (0.187 g, 83.8%). The characterization was done with NMR; ^{1}H NMR (300 MHz, Chloroform-*d*) δ 7.98 (s, 1H), 7.81 (d, J = 8.7 Hz, 1H), 7.50 (d, J = 2.2 Hz, 1H), 7.13 (dd, J = 8.7, 2.2 Hz, 1H), 4.41 (q, J = 7.1 Hz, 2H), 1.42 (t, J = 7.1 Hz, 3H).

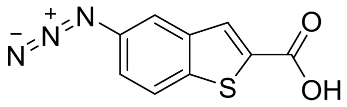

Compound 12. The compound synthesis was previously reported by Fang X.[8] The procedure was adopted from Ambrose A et al.[2] and was slightly modified. Briefly, Compound 12 (0.1 gr, 4.04 mmol, 1.0 eq.) was dissolved in THF/ethanol/water (3:2:1 ratio), NaOH (0.057 gr, 14.14 mmol, 3.5 eq.) was added and heated at 60 °C overnight. THF and ethanol were removed, and the crude was acidified with 1 N HCl and dissolved in a minimal amount of water. The precipitate was then centrifuged (2500 rpm, 10 minutes, 4 °C) and decanted to obtain Compound 13 (0.0814 gr, 88%). The characterization was done with NMR; ^{1}H NMR (300 MHz, DMSO-d_6) δ 8.02 (d, J = 8.7 Hz, 1H), 7.92 (s, 1H), 7.74 (d, J = 2.2 Hz, 1H), 7.20 (dd, J = 8.7, 2.3 Hz, 1H), 3.17 (s, 1H).

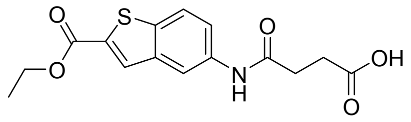

Compound 13. The procedure was adopted from Zhou F et al.[9] and was slightly modified. Briefly, Compound 11 (0.07 g, 0.316 mmol, 1 eq.) was dissolved in DMF (10 mL), and succinic anhydride (0.192 g, 1.898 mmol, 6 eq.) was added. The reaction mixture was heated to 90°C for 8 hours. The reaction was cooled to room temperature, diluted with EtOAc (70 mL) and water (40 mL). After separation, the aqueous layer was extracted twice with EtOAc (70 mL). The combined organic layers were washed once with brine (40 mL), dried over $MgSO_4$, and evaporated under reduced pressure. The crude product was purified using column chromatography with a gradient up to 20% MeOH in DCM to obtain Compound 14 (0.068 g, 46.8%). The characterization was done with MALDI-TOF; calculated MS: 321.35, and measured: 321.09.

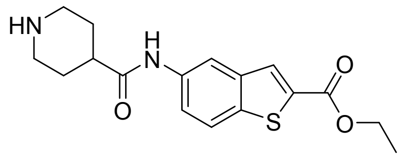

Compound 14. The procedure was adopted from Zhou F et al.[9] and was slightly modified. Briefly, To a solution of N-Boc-piperidine-4-carboxylic acid (0.05 g, 0.218 mmol, 1.2 eq.) in dry DMF (2 mL), DIPEA (0.141 g, 185.43 µL, 1.09 mmol, 5 eq.) and HATU (0.124 g, 0.327 mmol, 1.5 eq.) were added and stirred for 10 minutes at room temperature. Then, Compound 11 (0.0579 g, 0.262 mmol, 1 eq.) was added and stirred at room temperature for 16 hours. After completion, the mixture was evaporated under reduced pressure. For Boc deprotection, Compound 15-Boc (1 eq.) was dissolved in a 50% TFA solution in DCM (10 mL) and reacted for 30 minutes at room temperature. After completion, the volatiles were evaporated under reduced pressure and purified using column chromatography with a gradient up to 20% MeOH in DCM to obtain Compound 15 (0.027 g, 37.97%). The characterization was done with MALDI-TOF; calculated MS: 332.42, and measured: 333.34.

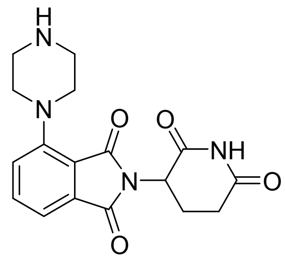

Compound 15. The compound synthesis was previously reported by Chen Z et al.[10] The procedure was, slightly modified. Briefly, Compound 7 (0.05 g, 0.18 mmol, 1 eq.) was dissolved in DMF (4 mL), and DIPEA (0.091 g, 0.54 mmol, 3 eq.) was added. N-Boc-piperazine (0.037 g, 0.2 mmol, 1.1 eq.) was separately dissolved in DMF (2 mL) and added to the reaction. The reaction mixture was heated to 50 °C for 4 hours. The reaction was cooled to room temperature, diluted with EtOAc (20 mL) and water (10 mL). The aqueous layer was then extracted twice with EtOAc (20 mL). The combined organic layers were washed once with brine (25 mL), dried over $MgSO_4$, and evaporated under reduced pressure. For Boc deprotection, Compound 16-Boc was dissolved in a 50% TFA solution in DCM (5 mL) and

reacted for 30 minutes at room temperature. After completion, the volatiles were evaporated under reduced pressure, and the crude product was purified using column chromatography with a gradient up to 55% MeOH in DCM to obtain Compound 16 (0.0716 g, 89.5%). The characterization was done with MALDI-TOF; calculated MS: 343.13, and measured MS: 343.33.

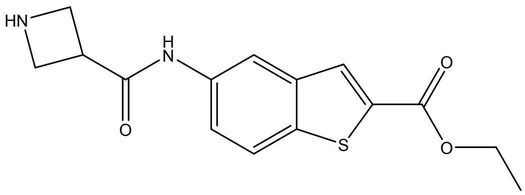

Compound 16. The procedure was adopted from Zhou F et al.[9] and was slightly modified. Briefly, To a solution of 1-Boc-azetidine-3-carboxylic acid (0.7 g, 0.347 mmol, 1.2 eq.) in dry DMF (2 mL)DIPEA (0.224 g, 295.8 µL, 1.735 mmol, 5 eq.) and HATU (0.198 g, 0.522 mmol, 1.5 eq.) were added. The mixture was stirred for 10 minutes at room temperature. Then, Compound 11 (0.0924 g, 0.417 mmol, 1 eq.) was added and stirred at room temperature for 16 hours. After completion, the reaction mixture was evaporated under reduced pressure. For Boc deprotection, Compound 17-Boc was dissolved in a 50% TFA solution in DCM (10 mL) and reacted for 30 minutes at room temperature. After completion, the volatiles were evaporated under reduced pressure, and the crude product was purified using column chromatography with 20% MeOH in DCM to obtain Compound 17(0.0922 g, 87.1%). The characterization was done with NMR and MALDI-TOF . ^{1}H NMR (300 MHz, DMSO-d_6) δ 10.50 (s, 1H), 8.40 (d, J = 1.9 Hz, 2H), 8.17 (s, 1H), 7.98 (d, J = 8.8 Hz, 1H), 7.66 (dd, J = 8.9, 2.0 Hz, 1H), 4.34 (q, J = 7.1 Hz, 3H), 3.81 (s, 1H), 1.33 (t, J = 7.1 Hz, 3H). ^{13}C NMR (75 MHz, DMSO-d_6) δ 169.67, 162.27, 139.22, 136.78, 136.61, 134.21, 131.12, 123.55, 120.75, 115.54, 61.78, 50.01, 47.37, 36.91, 14.48. calculated MS: 304.36 and measured MS: 305.12.

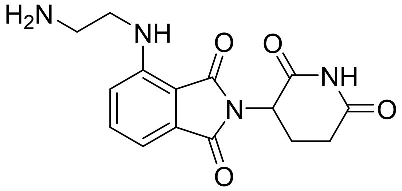

Compound 17. The compound synthesis was previously reported by Qiu X et al.[11] The procedure was, slightly modified. Briefly, Compound 7 (0.222 g, 0.8 mmol, 1 eq.) was dissolved in DMF (4 mL), and DIPEA (0.410 g, 2.4 mmol, 3 eq.) was added. N-Boc-ethylenediamine (0.1402 g, 0.8 mmol, 1.1 eq.) was separately dissolved in DMF (2 mL) and added to the reaction. The reaction mixture was heated to 90 °C for 4 hours. The reaction was cooled to room temperature, diluted with EtOAc (20 mL) and water (10 mL). The aqueous layer was then extracted twice with EtOAc (20 mL). The combined organic layers were washed once with brine (25 mL), dried over $MgSO_4$, and evaporated under reduced pressure. For Boc deprotection, Compound 18-Boc was dissolved in a 50% TFA solution in DCM (5 mL) and reacted for 30 minutes at room temperature. After completion, the volatiles were evaporated under

reduced pressure, and the crude product was then purified using column chromatography with a gradient up to 15% MeOH in DCM to obtain Compound 18 (0.157 g, 61.8%). The characterization was done with MALDI TOF; calculated MS: 316.31 and measured MS: 316.92.

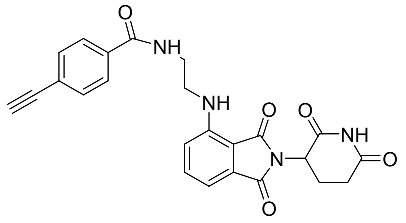

Compound 18. The procedure was adopted from Zhou F et al.[9] and was slightly modified. Briefly, To a solution of 4-Ethynylbenzoic acid (0.01 g, 0.068 mmol, 1.2 eq.) in dry DMF (2 mL), DIPEA (0.053 g, 0.071 mL, 0.412 mmol, 5 eq.) and HATU (0.0469 g, 0.124 mmol, 1.5 eq.) were added and stirred for 10 minutes at room temperature. Then Compound 18 (0.026 g, 0.0825 mmol, 1 eq.) was added and stirred at room temperature for 16 hours. After completion, the mixture was evaporated under reduced pressure and purified using column chromatography with a gradient up to 4% MeOH in DCM to obtain Compound 19 (0.0142 g, 18%). The compound was used directly in the subsequent step, with characterization performed solely by MALDI-TOF; calculated MS: 444.45 and measured: 444.32.

FINAL MOLECULES:

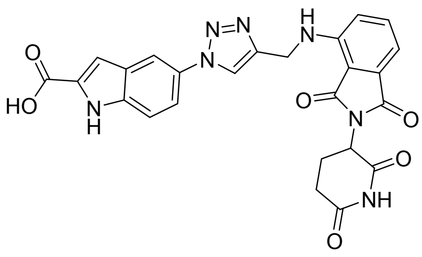

PRO-HuR1. Compound 3 (26.8 mg, 0.123 mmol, 1 eq.) and Compound 7 (46.1 mg, 0.148 mmol, 1.2 eq.) were dissolved in DMF. Copper sulfate pentahydrate (3.095 mg, 0.0123 mmol, 10% mmol), sodium ascorbate (9.79 mg, 0.492 mmol, 40% mmol), DABCO (2.76 mg, 0.0246 mmol, 20% mmol), and acetic acid (1.48 mg, 1.41 µL, 0.0246 mmol, 20% mmol) were added. The reaction mixture was stirred at room temperature for 3 hours. After completion, the mixture was evaporated under reduced pressure and purified using column chromatography with a gradient up to 20% MeOH in DCM, and UV-HPLC (5% - 90% ACN in H2O + 0.1% TFA, 55 min gradient) to obtain PRO-HuR1 (2.3 mg, 3.5%). Purity was evaluated using analytical HPLC (Retention time = 6.7 minutes, purity: 100%). The characterization was done with NMR and MALDI TOF; ^{1}H NMR (300 MHz, DMSO-d_6) δ 11.34 (s, 1H), 11.09 (s, 1H), 8.61 (s, 1H), 8.35 (s, 1H), 7.94 (d, J = 1.9 Hz, 1H), 7.59 (q, J = 7.3, 6.8 Hz, 1H), 7.53 – 7.46 (m, 2H), 7.24 (d, J = 8.6 Hz, 1H), 7.13 (t, J = 6.3 Hz, 1H), 7.07 (d, J = 7.0 Hz, 1H), 6.73 (s, 1H), 5.07 (dd, J = 12.6, 5.2 Hz, 1H), 4.69 (d, J = 6.0 Hz, 2H), 2.97 – 2.81 (m, 3H), 2.09 – 1.97 (m, 1H). ^{13}C NMR (126 MHz, DMSO-d_6) δ 172.83, 170.09, 168.81, 167.33, 167.10, 161.05, 145.88, 145.29, 136.23, 135.70,

132.16, 129.52, 127.40, 121.39, 117.71, 112.99, 112.87, 111.01, 109.85, 62.39, 41.45, 37.71, 30.99, 22.16. Calculated MS: 514.47 [M+H]+ and measured: 514.37.

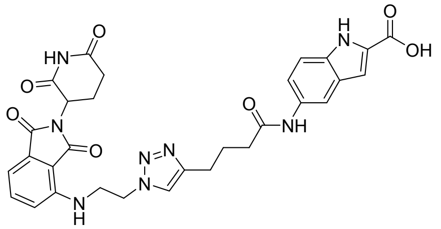

PRO-HuR2. Compound 5 (20 mg, 0.073 mmol, 1 eq.) and Compound 9 (31 mg, 0.088 mmol, 1.2 eq.) were dissolved in DMF. Copper sulfate pentahydrate (1.86 mg, 0.007 mmol, 10% mmol), sodium ascorbate (5.9 mg, 0.03 mmol, 40% mmol), DABCO (1.66 mg, 0.015 mmol, 20% mmol), and acetic acid (0.88 mg, 0.015 mmol, 0.85 µL, 20% mmol) were added. The reaction mixture was stirred at room temperature for 3 hours. After completion, the mixture was evaporated under reduced pressure and purified using column chromatography with a gradient up to 20% MeOH in DCM, and UV-HPLC (5% - 90% ACN in H2O + 0.1% TFA, 55 min gradient) to obtain PRO-HuR2 (5.1 mg, 11.2%). Purity was evaluated using analytical HPLC (Retention time = 4.04 minutes, purity: 98.2%). The characterization was done with NMR and MALDI TOF; ^{1}H NMR (300 MHz, DMSO-d_6) δ 11.43 (s, 1H), 11.08 (s, 1H), 9.71 (s, 1H), 7.94 (d, *J* = 5.2 Hz, 2H), 7.54 (t, *J* = 7.8 Hz, 1H), 7.29 (d, *J* = 5.5 Hz, 2H), 7.03 (d, *J* = 6.4 Hz, 3H), 6.90 (s, 1H), 6.76 (s, 1H), 5.04 (dd, *J* = 12.8, 5.3 Hz, 1H), 4.53 (d, *J* = 6.4 Hz, 2H), 3.79 (d, *J* = 6.8 Hz, 2H), 2.84 (d, *J* = 13.6 Hz, 2H), 2.64 (t, *J* = 7.2 Hz, 4H), 2.33 (t, *J* = 7.3 Hz, 2H), 1.88 (t, *J* = 7.5 Hz, 2H). ^{13}C NMR (126 MHz, DMSO-d_6) δ 173.33, 170.91, 170.59, 169.18, 167.75, 147.09, 146.43, 136.75, 132.70, 122.97, 117.56, 111.49, 110.09, 49.08, 42.62, 36.27, 31.49, 29.54, 25.71, 25.17, 22.65 . Calculated MS: 613.60 [M+H]+ and measured: 613.10.

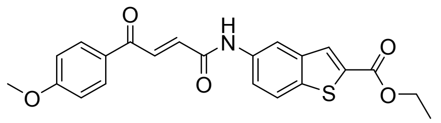

MG-HuR1. To a solution of 3-(4-Methoxybenzoyl)acrylic acid (112 mg, 0.542 mmol, 1.2 eq.) in dry DMF (2 mL), DIPEA (292 mg, 0.4 mL, 2.26 mmol, 5 eq.) and HATU (257.8 mg, 0.678 mmol, 1.5 eq.) were added, and the mixture was stirred for 10 minutes at room temperature. Then Compound 10 (100 mg, 0.452 mmol, 1 eq.) was added and stirred at room temperature for 16 hours. After completion, the mixture was evaporated under reduced pressure and purified using column chromatography with a gradient up to 20% MeOH in DCM and UV-HPLC (5% - 90% ACN in H2O + 0.1% TFA, 65 min gradient) to obtain MG-HuR1 (45.5 mg, 24.6%). Purity was evaluated using analytical HPLC (Retention time = 10.2 minutes, purity: 100%). The characterization was done with NMR and MALDI TOF; ^{1}H NMR (300 MHz, DMSO-d_6) δ 10.82 (s, 1H), 8.51 (d, J = 1.9 Hz, 1H), 8.22 (s, 1H), 8.11 – 8.05 (m, 2H), 8.03 (s, 1H), 7.96 (d, J = 15.2 Hz, 1H), 7.75 (dd, J =

8.9, 2.0 Hz, 1H), 7.22 (d, J = 15.2 Hz, 1H), 7.12 (d, J = 8.7 Hz, 2H), 4.36 (q, J = 7.1 Hz, 2H), 3.88 (s, 3H), 1.34 (t, J = 7.1 Hz, 3H).). ^{13}C NMR (75 MHz, DMSO-d_6) δ 162.75, 161.25, 140.83, 139.01, 137.77, 134.41, 132.24, 128.31, 128.00, 126.68, 125.96, 125.89, 125.55, 118.85, 112.79, 111.81, 107.78, 60.46, 14.36. Calculated MS: 410.46 $[M+H]^+$ and measured: 410.10.

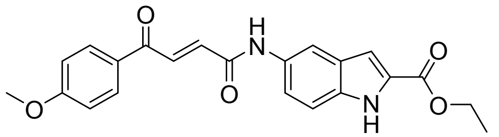

MG-HuR2. To a solution of 4-(4-Methoxybenzoyl)acrylic acid (1.2 eq.) in dry DMF (2 mL), DIPEA (316.4 mg, 2.4 mmol, 5 eq.) and HATU (280 mg, 0.8 mmol, 1.5 eq.) were added. The reaction mixture was stirred for 10 minutes at room temperature. Then Compound 1 (100 mg, 0.49 mmol, 1 eq.) was added and stirred at room temperature for 16 hours. After completion, the mixture was evaporated under reduced pressure and purified using column chromatography with a gradient up to 20% MeOH in DCM and UV-HPLC (5% - 90% ACN in H2O + 0.1% TFA, 45 min gradient) to obtain MG-HuR2 (0.185 g, 96%). Purity was evaluated using analytical HPLC (Retention time = 7.7 minutes, purity: 99.9%). The characterization was done with NMR and MALDO TOF; ^{1}H NMR (300 MHz, DMSO-d_6) δ 10.58 (s, 1H), 8.26 (s, 1H), 8.13 (dd, J = 8.8, 2.5 Hz, 2H), 7.97 (dd, J = 15.8, 2.2 Hz, 1H), 7.50 (s, 2H), 7.28 (dd, J = 15.1, 2.8 Hz, 1H), 7.22 – 7.13 (m, 4H), 4.39 (dt, J = 8.9, 6.6 Hz, 2H), 3.94 – 3.91 (m, 3H), 1.39 (td, J = 7.2, 2.3 Hz, 4H). ^{13}C NMR (75 MHz, DMSO-d_6) δ 188.08, 164.20, 162.11, 161.63, 136.81, 134.99, 132.79, 132.34, 131.66, 130.03, 128.52, 127.05, 119.19, 114.79, 113.28, 112.47, 108.26, 60.89, 56.13, 40.40, 39.52, 14.77.Calculated MS: 393.41 $[M+H]^+$ and measured: 393.41.

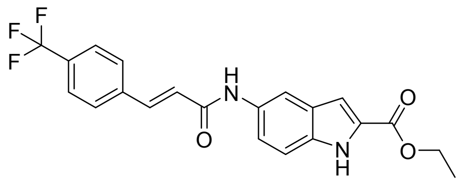

MG-HuR3. To a solution of 2-Propenoic acid, 3-[4-(trifluoromethyl)phenyl]-, (63.5 mg, 0.293 mmol, 1.2 eq.) in dry DMF (2 mL), DIPEA (158.2 mg, 1.2 mmol, 0.2 mL, 5 eq.) and HATU (139.6 mg, 0.37 mmol, 1.5 eq.) were added and stirred for 10 minutes at room temperature. Then Compound 1 (50 mg, 0.245 mmol, 1 eq.) was added, and the reaction was stirred at room temperature for 16 hours. After completion, the mixture was evaporated under reduced pressure and purified using column chromatography with a gradient up to 20% MeOH in DCM and UV-HPLC (5% - 90% MeOH in H2O + 0.1% TFA, 45 min gradient) to obtain MG-HuR3 (4.4 mg, 4.5%). Purity was evaluated using analytical HPLC (Retention time = 8.6 minutes, purity: 100%). The characterization was done with NMR and MALDI TOF; ^{1}H NMR (300 MHz, DMSO-d_6) δ 11.86 (s, 1H), 10.28 (s, 1H), 8.18 (d, J = 1.7 Hz, 1H), 7.88 – 7.76 (m, 4H), 7.64 (d, J = 15.6 Hz, 1H), 7.49 – 7.38 (m, 2H), 7.14 (s, 1H), 7.00 (d, J =

15.7 Hz, 1H), 4.34 (q, J = 7.1 Hz, 2H), 1.34 (t, J = 7.1 Hz, 3H). ^{13}C NMR (75 MHz, DMSO-d_6) δ 188.00, 164.26, 162.70, 162.41, 141.24, 139.42, 137.20, 136.83, 136.22, 134.50, 133.52, 131.72, 131.35, 129.94, 123.89, 120.87, 115.91, 114.82, 61.95, 56.15, 14.62. Calculated MS: 403.37 $[M+H]^+$ and measured: 403.31.

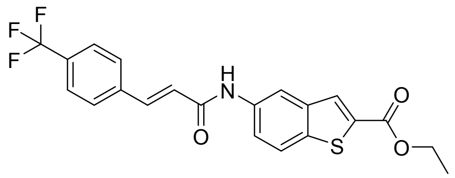

MG-HuR4. To a solution of 2-Propenoic acid, 3-[4-(trifluoromethyl)phenyl] (117 mg, 0.542 mmol, 1.2 eq.) in dry DMF (2 mL), DIPEA (292 mg, 0.4 mL, 2.26 mmol, 5 eq.) and HATU (257.8 mg, 0.678 mmol, 1.5 eq.) were added, and the mixture was stirred for 10 minutes at room temperature. Then Compound 10 (1 eq.) was added and stirred at room temperature for 16 hours. After completion, the mixture was evaporated under reduced pressure and purified using column chromatography with a gradient up to 20% MeOH in DCM. Purity was evaluated using analytical HPLC (Retention time = 9.8 minutes, purity: 99.7%) to obtain MG-HuR4 (101.9 mg, 53.4%). The characterization was done with NMR and MALDI TOF; ^{1}H NMR (300 MHz, DMSO-d_6) δ 10.58 (s, 1H), 8.54 (d, J = 2.0 Hz, 1H), 8.26 (s, 1H), 8.07 (d, J = 8.8 Hz, 1H), 7.94 – 7.69 (m, 6H), 7.05 (d, J = 15.8 Hz, 1H), 4.40 (q, J = 7.1 Hz, 2H), 1.39 (t, J = 7.1 Hz, 3H). ^{13}C NMR (126 MHz, DMSO-d_6) δ 163.29, 162.04, 139.06, 138.83, 138.61, 136.79, 136.41, 133.97, 130.96, 129.65, 129.40, 128.44, 128.10, 126.01, 125.98, 125.95, 125.24, 125.06, 123.40, 120.49, 115.28, 61.53, 14.22. Calculated MS: 514.4420.42 $[M+H]^+$ and measured: 420.24.

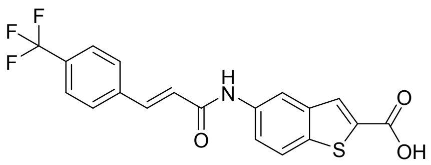

MG-HuR5. MG-HuR4 (50 mg, 0.12 mmol, 1.0 eq.) was dissolved in THF/ethanol/water (3:2:1 ratio), and NaOH (16.7 mg, 0.42 mmol, 3.0 eq.) was added and heated at 60 °C overnight. THF and ethanol were removed, and the crude was acidified with 1 N HCl and dissolved in a minimal amount of water. The precipitate was then centrifuged (2500 rpm, 10 minutes, 4 °C) and decanted. The product was purified using column chromatography with a gradient up to 20% MeOH in DCM to obtain MG-HuR5 (11.7 mg, 24.9%). Purity was evaluated using analytical HPLC (Retention time = 7.6 minutes, purity: 99.8%). The characterization was done with NMR and MALFI TOF; ^{1}H NMR (300 MHz, DMSO-d_6) δ 8.01 (d, J = 11.4 Hz, 3H), 7.89 (q, J = 8.3 Hz, 5H), 7.80 – 7.61 (m, 2H), 3.84 (s, 2H), 3.39 (s, 15H), 3.22 (s, 4H). ^{13}C NMR (126 MHz, DMSO-d_6) δ 170.82, 164.14, 163.60, 140.02, 139.30, 138.87, 136.76,

129.78, 126.42, 126.39, 126.36, 125.66, 125.60, 123.61, 119.82, 115.28, 60.23, 21.24, 14.56. Calculated MS: 392.36 $[M+H]^+$ and measured: 392.19.

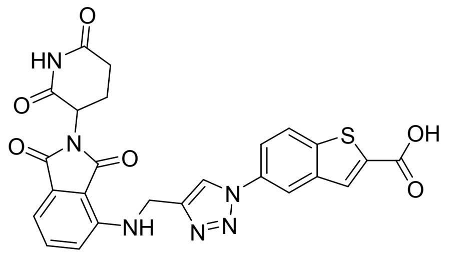

PRO-HuR3. Compound 12 (47 mg, 0.2 mmol, 1.2 eq.) and Compound 7 (55.6 mg, 0.175 mmol, 1 eq.) were dissolved in DMF. Copper sulfate pentahydrate (4.4 mg, 0.0175 mmol; 10% mmol), sodium ascorbate (13.94 mg, 0.07 mmol; 40% mmol), DABCO (3.9 mg, 0.035 mmol; 20% mmol), and acetic acid (2.1 mg, 2.001 µL, 0.035 mmol; 20% mmol) were added. The reaction was stirred at room temperature for 3 hours. After completion, the mixture was evaporated under reduced pressure and purified by using column chromatography with a gradient up to 20% MeOH in DCM, and UV-HPLC (5% - 90% ACN in H_2O + 0.1% TFA, 75 min gradient) to obtain PRO-HuR3 (5.1 mg, 4.6%). Purity was evaluated using analytical HPLC (Retention time = 5.3 minutes, purity: 99.4%). The characterization was done with NMR and MALDI TOF; ^1H NMR (300 MHz, DMSO-d_6) δ 11.09 (s, 1H), 8.76 (s, 1H), 8.44 (d, *J* = 2.3 Hz, 1H), 8.17 (d, *J* = 8.5 Hz, 2H), 7.96 (d, *J* = 6.1 Hz, 1H), 7.92 (d, *J* = 2.2 Hz, 1H), 7.62 – 7.56 (m, 1H), 7.20 (s, 1H), 7.16 (d, *J* = 5.8 Hz, 1H), 7.07 (d, *J* = 7zz 1H), 5.07 (dd, *J* = 12.7, 5.4 Hz, 1H), 4.72 (d, *J* = 6.1 Hz, 3H), 2.31 – 2.24 (m, 1H), 2.08 – 1.97 (m, 2H). ^{13}C NMR (126 MHz, DMSO-d_6) δ 173.03, 170.29, 168.97, 167.51, 146.10, 146.03, 140.91, 139.99, 136.45, 134.05, 132.36, 124.51, 121.59, 117.88, 116.34, 111.26, 110.08, 51.45, 37.89, 31.18, 22.36. Calculated MS: 553.51 $[M+Na]^+$ and measured: 553.18.

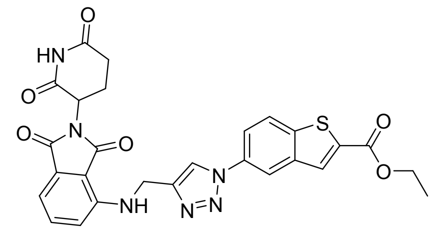

PRO-HuR4. Compound 11 (27.3 mg, 0.11 mmol, 1 eq.) and Compound 7 (28.6 mg, 0.092 mmol, 1.2 eq.) were dissolved in DMF. Copper sulfate pentahydrate (2.31 mg, 0.0092 mmol; 10% mmol), sodium ascorbate (7.33 mg, 0.368 mmol; 40% mmol), DABCO (2.1 mg, 0.0184 mmol; 20% mmol), and acetic acid (1.104 mg, 1.06 µL, 0.0184 mmol; 20% mmol) were added. The reaction was stirred at room temperature for 3 hours. After completion, the mixture was evaporated under reduced pressure and purified by using column chromatography with a gradient up to 20% MeOH in DCM to obtain PRO-HuR4 (19.3 mg, 37.6%). Purity was evaluated using analytical HPLC (Retention time = 6.5 minutes, purity: 97.3%). The characterization was done with NMR and MALDI TOF; ^1H NMR (300

MHz, Chloroform-*d*) δ 8.22 (d, *J* = 13.6 Hz, 2H), 8.09 (s, 1H), 8.04 – 7.89 (m, 2H), 7.90 – 7.77 (m, 1H), 7.50 (t, *J* = 7.9 Hz, 1H), 7.14 (dd, *J* = 7.2, 1.9 Hz, 1H), 7.03 (d, *J* = 8.6 Hz, 1H), 6.78 (s, 1H), 5.05 – 4.88 (m, 1H), 4.75 (s, 2H), 4.43 (dt, *J* = 8.5, 6.3 Hz, 2H), 2.95 – 2.04 (m, 4H), 1.43 (td, *J* = 7.1, 1.9 Hz, 3H). ^{13}C NMR (75 MHz, DMSO-d_6) δ 173.30, 170.83, 170.56, 169.25, 167.78, 146.52, 146.31, 141.32, 139.51, 136.73, 136.00, 134.81, 132.65, 131.20, 125.15, 121.93, 120.29, 118.16, 117.36, 111.56, 110.38, 62.24, 60.25, 49.07, 31.48, 21.26, 14.63. Calculated MS: 581.57 [M+Na]$^+$ and measured: 581.16.

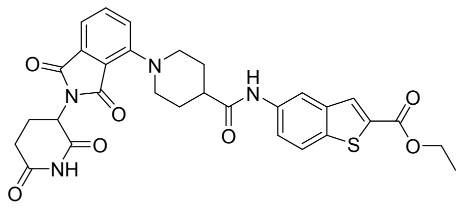

PRO-HuR5. Compound 6 (20.8 mg, 0.072 mmol, 1 eq.) was dissolved in DMF (10 mL), and DIPEA (29.2 mg, 38.43 µL, 0.225 mmol, 3 eq.) was added. Compound 14 (27.5 mg, 0.082 mmol, 1.1 eq.) was separately dissolved in DMF (2 mL) and added to the reaction. The reaction mixture was heated to 90 °C for 6 hours. The reaction was cooled to room temperature and evaporated under reduced pressure. The crude product was purified using column chromatography with a gradient up to 20% MeOH in DCM to obtain PRO-HuR5 (19.1 mg, 25%). Purity was evaluated using analytical HPLC (Retention time = 8.0 minutes, purity: 99.2%). The characterization was done with NMR and MALDI TOF; ^{1}H NMR (300 MHz, Chloroform-*d*) δ 8.53 (s, 1H), 8.28 (s, 1H), 7.95 (s, 1H), 7.87 (s, 1H), 7.70 (d, *J* = 8.7 Hz, 1H), 7.51 (t, *J* = 8.3 Hz, 2H), 7.36 (d, *J* = 7.1 Hz, 1H), 7.14 (d, *J* = 8.4 Hz, 1H), 5.01 (q, *J* = 6.0, 5.5 Hz, 1H), 4.40 (q, *J* = 7.2 Hz, 2H), 3.75 (d, *J* = 10.6 Hz, 2H), 2.95 (s, 2H), 2.91 – 2.72 (m, 4H), 2.52 (s, 1H), 2.13 (s, 4H), 1.41 (t, *J* = 7.1 Hz, 3H). ^{13}C NMR (75 MHz, DMSO-d_6) δ 173.36, 172.75, 169.97, 167.03, 166.25, 161.94, 149.93, 138.87, 136.90, 135.84, 135.71, 133.67, 133.61, 130.78, 123.97, 123.05, 120.44, 116.52, 115.00, 114.59, 61.37, 50.52, 50.39, 48.73, 42.25, 30.91, 28.35, 22.01, 14.11. Calculated MS: 589.64 [M+H]$^+$ and measured: 589.47.

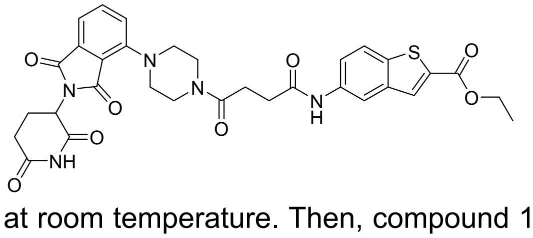

PRO-HuR6. To a solution of compound 13 (15 mg, 0.047 mmol, 1.2 eq.) in dry DMF (2 mL), DIPEA (31 mg, 35 µL, 0.24 mmol, 5 eq.) and HATU (53.11 mg, 0.14 mmol, 1.5 eq.) were added and stirred for 10 minutes at room temperature. Then, compound 15 (20 mg, 0.056 mmol, 1 eq.) was added and stirred at room temperature for 16 hours. After completion, the mixture was evaporated under reduced pressure and purified by column chromatography with a gradient up to 20% MeOH in DCM to obtain PRO-HuR6 (5.5 mg, 9.1%). Purity was evaluated using analytical HPLC (Retention time =

7.3 minutes, purity: 98.9%). The characterization was done with NMR and MALDI TOF; ^{1}H NMR (300 MHz, Chloroform-*d*) δ 8.71 (s, 1H), 8.35 (s, 1H), 8.24 (d, *J* = 1.9 Hz, 1H), 7.95 (s, 1H), 7.72 (d, *J* = 8.7 Hz, 1H), 7.61 (t, *J* = 7.8 Hz, 1H), 7.49 – 7.41 (m, 2H), 7.12 (d, *J* = 8.3 Hz, 1H), 4.96 (dd, *J* = 12.1, 5.4 Hz, 1H), 4.39 (q, *J* = 7.1 Hz, 2H), 3.81 (dt, *J* = 37.0, 5.0 Hz, 4H), 3.40 – 3.23 (m, 4H), 2.88 – 2.69 (m, 7H), 2.17 – 2.07 (m, 1H), 1.40 (t, *J* = 7.1 Hz, 3H). ^{13}C NMR (126 MHz, DMSO-d_6) δ 172.49, 170.50, 169.71, 169.67, 166.71, 166.05, 161.70, 149.14, 138.64, 136.76, 135.64, 135.38, 133.38, 133.31, 130.52, 123.57, 122.79, 119.96, 116.63, 114.94, 114.38, 61.11, 59.45, 50.51, 49.96, 44.42, 31.06, 30.64, 27.24, 21.73, 20.45, 13.86. Calculated MS: 668.69 $[M+Na]^+$ and measured: 668.42.

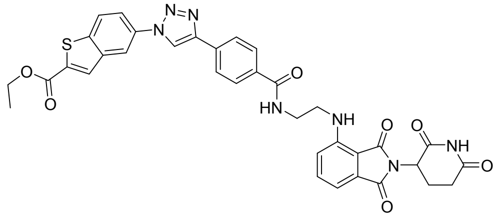

PRO-HuR7. Compound 11 (9.48 mg, 0.038 mmol, 1.2 eq.) and compound 18 (14.2 mg, 0.032 mmol, 1 eq.) were dissolved in DMF. Copper sulfate pentahydrate (0.82 mg, 0.0032 mmol; 10% mmol), sodium ascorbate (2.55 mg, 0.0128 mmol; 40% mmol), DABCO (0.72 mg, 0.0064 mmol; 20% mmol), and Acetic acid (0.0064 mg, 0.38 mmol, 0.4 mL, 20% mmol) were added. The reaction was stirred at room temperature for 3 hours. After completion, the mixture was evaporated under reduced pressure and purified by column chromatography with a gradient up to 20% MeOH in DCM to obtain PRO-HuR7 (8.5 mg, 39%). Purity was evaluated using analytical HPLC (Retention time = 8.4 minutes, purity: 91%). The characterization was done with NMR and MALDI TOF; ^{1}H NMR (300 MHz, DMSO-d_6) δ 11.10 (s, 1H), 9.49 (s, 1H), 8.79 (s, 1H), 8.65 (d, J = 2.1 Hz, 1H), 8.35 (d, J = 4.0 Hz, 1H), 8.16 – 8.02 (m, 4H), 7.98 (d, J = 8.3 Hz, 2H), 7.60 (t, J = 7.8 Hz, 1H), 7.28 (d, J = 8.6 Hz, 1H), 7.04 (d, J = 7.0 Hz, 1H), 6.88 (s, 1H), 5.06 (dd, J = 12.9, 5.3 Hz, 1H), 4.39 (q, J = 7.1 Hz, 2H), 3.51 (s, 4H), 3.03 – 2.75 (m, 2H), 2.11 – 1.95 (m, 2H), 1.37 (d, J = 7.1 Hz, 3H). ^{13}C NMR (126 MHz, DMSO-d_6) δ 173.17, 170.41, 169.03, 167.63, 166.71, 162.03, 146.97, 146.67, 141.34, 139.37, 136.55, 135.97, 134.57, 134.11, 133.08, 132.51, 131.03, 128.32, 125.41, 125.11, 121.12, 120.07, 117.54, 117.23, 110.92, 109.60, 62.11, 48.82, 41.63, 39.11, 31.25, 22.45, 14.43. Calculated MS: 692.74 $[M+H]^+$ and measured: 692.33.

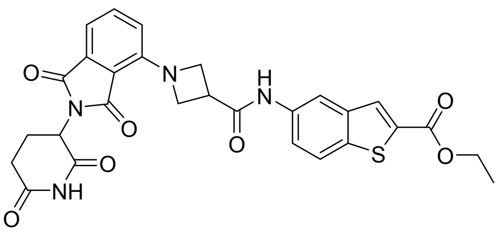

PRO-HuR8. Compound 6 (76 mg, 0.275 mmol, 1 eq.) was dissolved in DMF (10 mL), and DIPEA (313 mg, 412 µL, 0.826 mmol, 3 eq.) was added. Compound 16 (92.2 mg, 0.3 mmol, 1.1 eq.) was separately dissolved in DMF (2 mL) and added to the reaction. The reaction mixture

was heated to 90 °C for 6 hours. After completion, the reaction was cooled to room temperature and evaporated under reduced pressure. The crude product was purified using column chromatography with a gradient up to 20% MeOH in DCM, followed by UV-HPLC (5% - 90% MeOH in H2O + 0.1% TFA, 55 min gradient) to obtain PRO-HuR8 (8.3 mg, 5.5%). Purity was evaluated using analytical HPLC (Retention time = 7.9 minutes, purity: 100%). The characterization was done with NMR and MALDI TOF; ^{1}H NMR (300 MHz, DMSO-d_6) δ 11.07 (s, 1H), 10.33 (s, 1H), 8.39 (d, J = 1.9 Hz, 1H), 8.16 (s, 1H), 7.99 (d, J = 8.8 Hz, 1H), 7.72 – 7.55 (m, 2H), 7.15 (d, J = 7.1 Hz, 1H), 6.86 (d, J = 8.5 Hz, 1H), 5.06 (dd, J = 12.8, 5.3 Hz, 1H), 4.44 (t, J = 8.9 Hz, 2H), 4.37 – 4.26 (m, 4H), 3.79 – 3.63 (m, 1H), 2.87 (ddd, J = 17.5, 14.0, 5.3 Hz, 1H), 2.09 – 1.91 (m, 1H), 1.33 (t, J = 7.1 Hz, 3H), 1.22 (d, J = 6.4 Hz, 2H). ^{13}C NMR (75 MHz, DMSO-d_6) δ 173.26, 171.00, 170.47, 167.66, 166.96, 162.43, 148.22, 139.38, 137.07, 136.65, 135.49, 134.31, 133.72, 131.27, 123.68, 120.97, 120.29, 115.72, 110.86, 61.91, 49.11, 39.53, 34.75, 31.41, 22.57, 14.62. Calculated MS: 561.58 $[M+H]^+$ and measured: 561.34.

4. Figures and Tables

Oligonucleotide	Sequence 5' to 3'	Experiment	Supplier
GAPDH-F	AAT GAA GGG GTC ATT GAT GG	RT-qPCR	IDT
GAPDH-R	AAG GTG AAG GTC GGA GTC AA	RT-qPCR	IDT
Bcl2-F	ATC GCC CTG TGG ATG ACT GAG T	RT-qPCR	IDT
Bcl2-R	GCC AGG AGA AAT CAA ACA GAG GC	RT-qPCR	IDT
FOXQ1-F	CGA AGG AAG AGG GTA CGA CG	RT-qPCR	IDT
FOXQ1-R	GAG GGA CGA ACA CCT CCA AC	RT-qPCR	IDT
5'FAM	UAUUUAUUUA	Fluorescence polarization	IDT

Table S1. Sequence of oligonucleotides used in the study

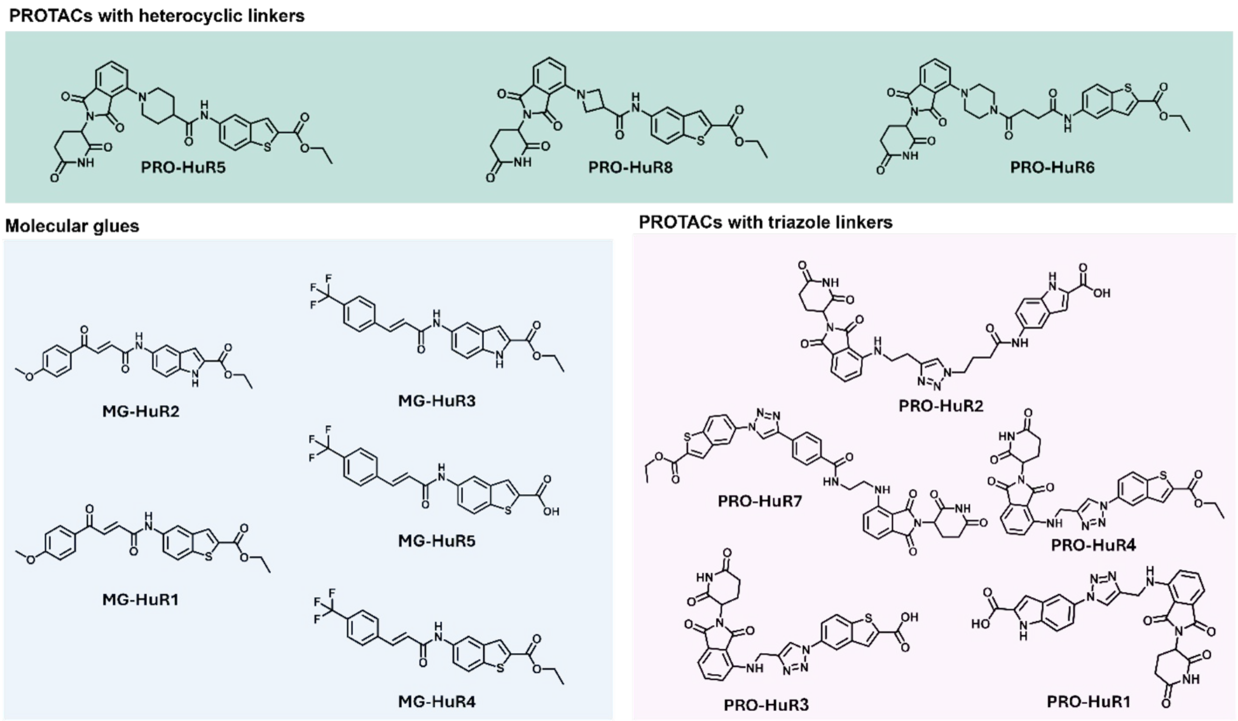

Figure S1. All synthesized compounds.

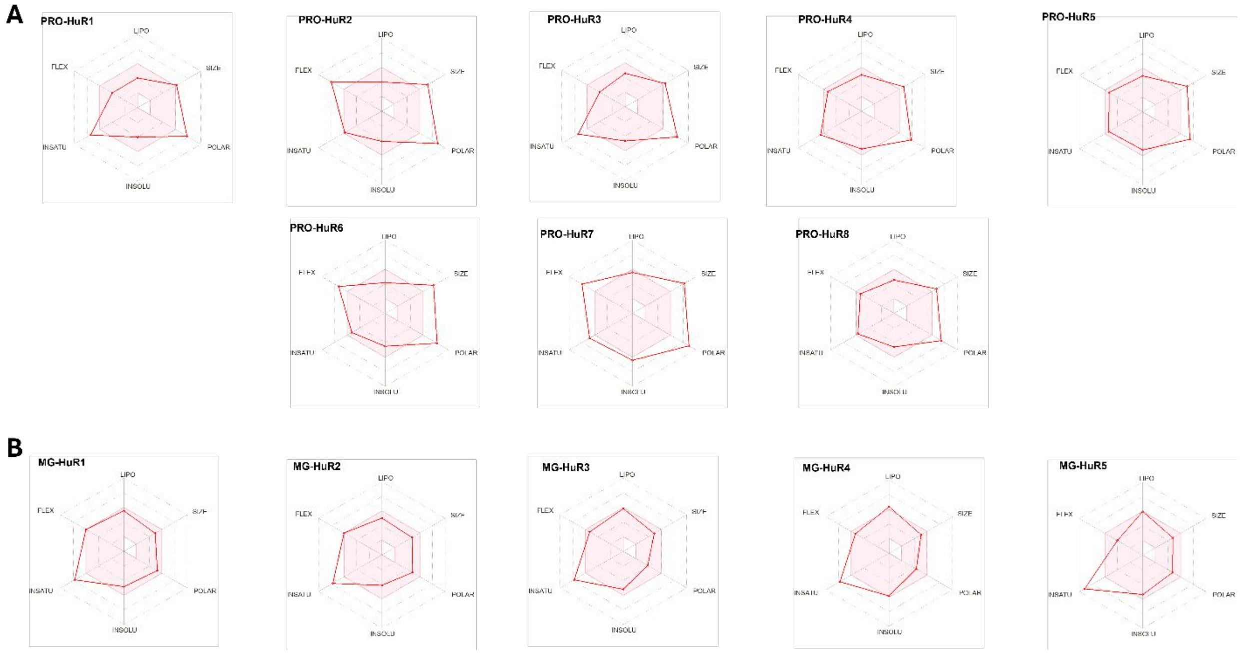

Figure S2. (A) Bioavailability for synthesized PROTACs. (B) Bioavailability for synthesized molecular glues.

A

PROTACs	Lipophilicity	Size	Polarity	Insolubility	Insaturation	Flexibility	Druglikeness		
							Lipinski	Pfizer	GSK
	XLOGP3	MW (g/mol)	TPSA Å^2	Log S (ESOL)	Fraction Csp3	Num. rotatable bonds		Yes/no	
PRO-HuR1	1.60	513.46	179.38	-4.02	0.16	6	No	Yes	No
PRO-HuR2	1.50	612.59	208.48	-4.12	0.27	12	No	Yes	No
PRO-HuR3	2.38	530.51	191.83	-4.69	0.16	6	No	Yes	No
PRO-HuR4	3.19	558.57	180.83	-5.15	0.22	8	No	Yes	No
PRO-HuR5	3.15	588.63	170.43	-5.21	0.33	8	No	Yes	No
PRO-HuR6	1.823	645.68	190.74	-4.51	0.34	11	No	Yes	No
PRO-HuR7	4.29	691.71	209.93	-6.42	0.20	12	No	Yes	No
PRO-HuR8	2.43	560.58	170.43	-4.60	0.29	8	No	Yes	No

B

Molecular glues	Lipophilicity	Size	Polarity	Insolubility	Insaturation	Flexibility	Druglikeness		
							Lipinski	Pfizer	GSK
	XLOGP3	MW (g/mol)	TPSA Å^2	Log S (ESOL)	Fraction Csp3	Num. rotatable bonds		Yes/no	
MG-HuR1	4.22	409.45	109.94	-4.83	0.14	9	Yes	Yes	No
MG-HuR2	3.33	392.40	97.49	-4.16	0.14	9	Yes	Yes	Yes
MG-HuR3	4.75	402.37	71.19	-5.18	0.14	8	Yes	No	No
MG-HuR4	5.64	419.42	83.64	-5.85	0.14	8	Yes	No	No
MG-HuR5	4.95	391.36	94.64	-5.40	0.05	6	Yes	No	No

Table S2. (A) Bioavailability and druglikeness for all PROTACs. (B) Bioavailability and druglikeness for all molecular glues.

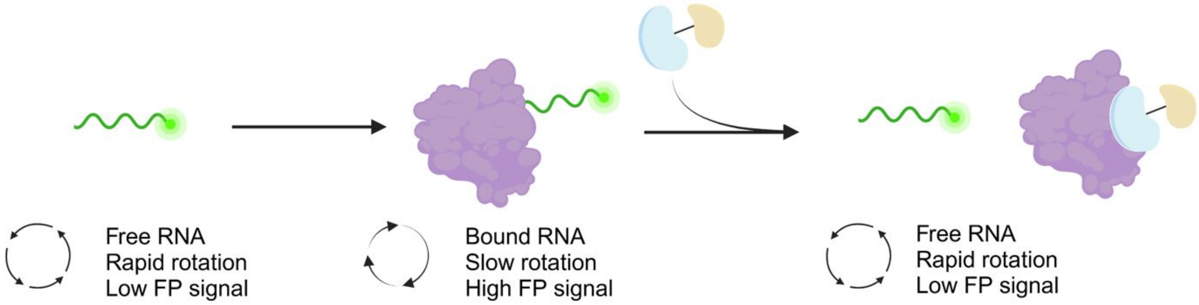

Figure S3. Fluorescence polarization (FP) assay. Free RNA rotates rapidly, producing a low FP signal, while an RNA-protein complex rotates more slowly, resulting in a high FP signal. The addition of a PROTAC or molecular glue compound displaces the RNA, restoring rapid rotation and lowering the FP signal.

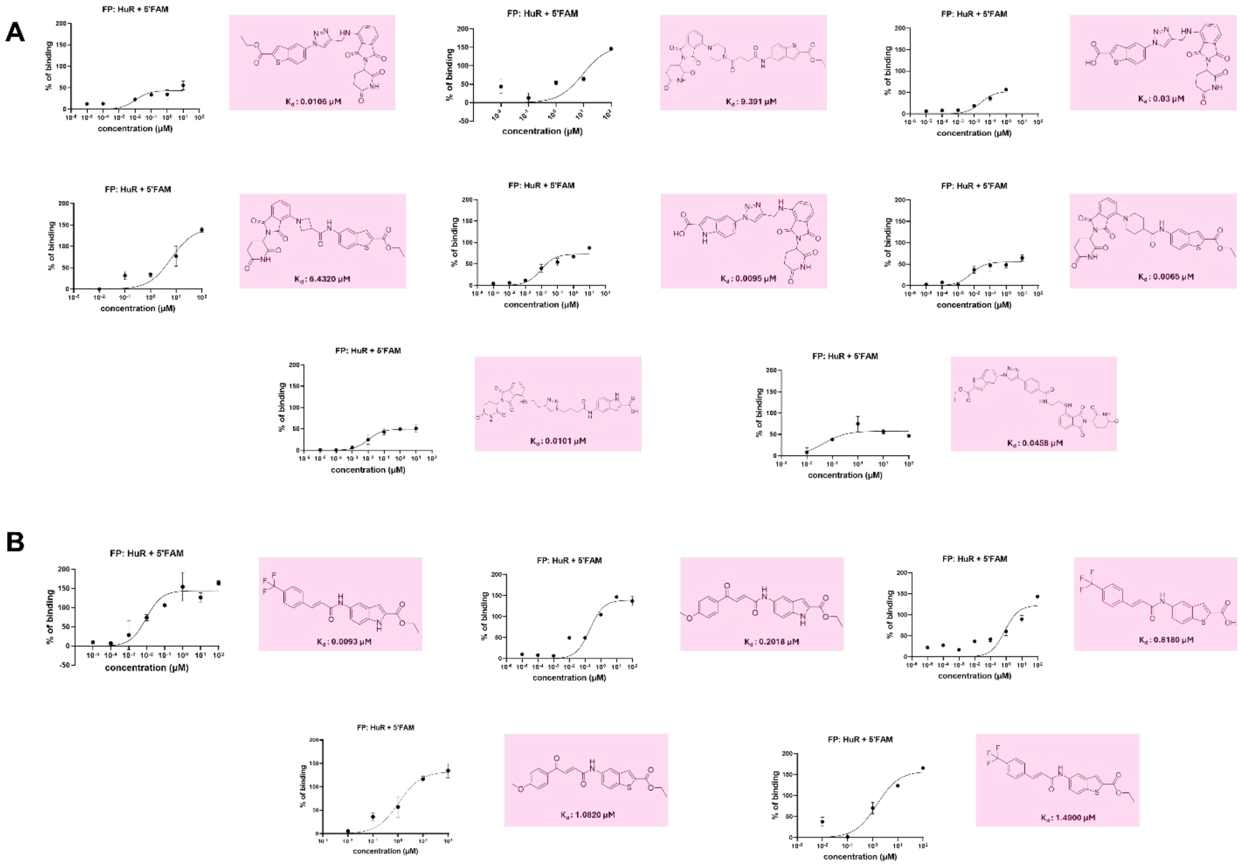

Figure S4. (A) PROTAC binding affinities for HuR, measured through inhibitory activity in FP assay. (B) molecular glues binding affinities for HuR, measured through inhibitory activity in FP assay.

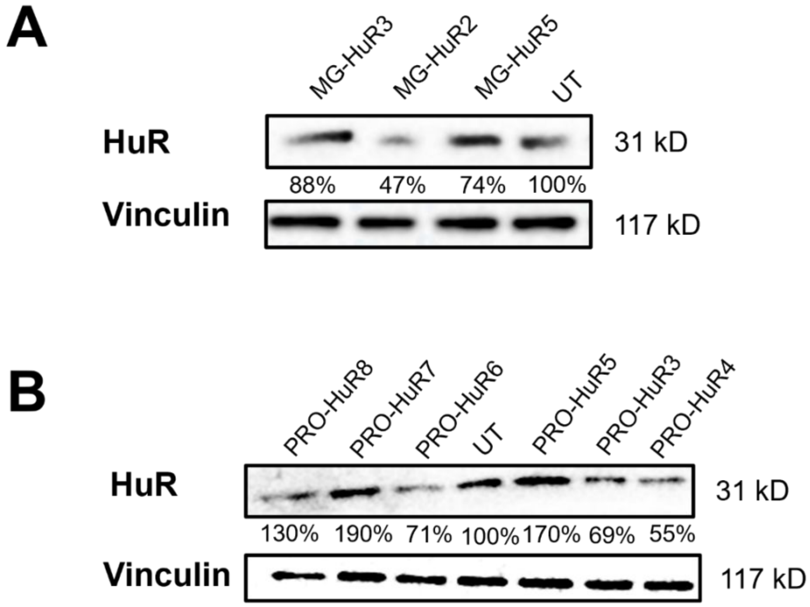

Figure S5. (A) HuR protein expression in MCF-7 cells after treatment with a 10 μM dose of selected molecular glues. (B) HuR protein expression in MCF-7 cells after treatment with a 10 μM dose of selected PROTACS.

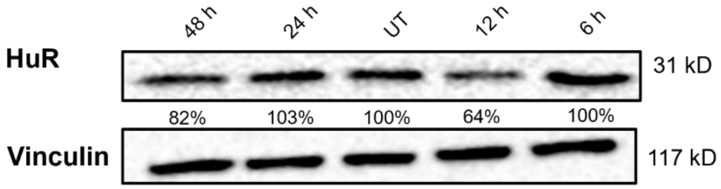

Figure S6. Time-dependent treatment of PRO-HuR4 in MCF-7, 10 μM dose.

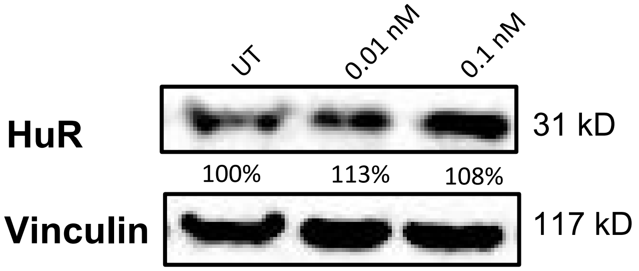

Figure S7. A dose-dependent treatment of PRO-HuR3 for 48 h.

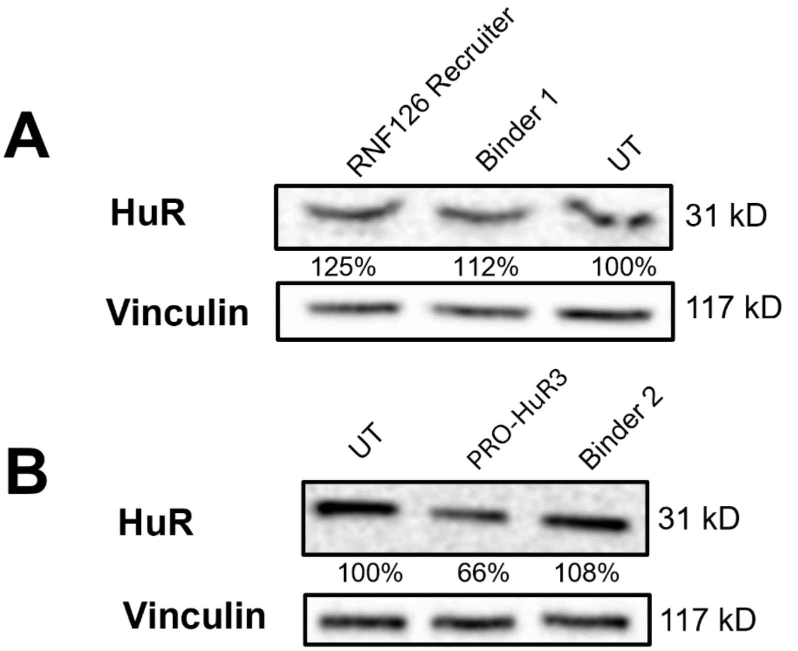

Figure S8. (A) MCF-7 cells were treated with 1 µM of Binder 1 and RNF126 Recruiter for 24 h. (B) MCF-7 cells were treated with 10 µM of Binder 2 and PRO-HuR3 for 48 h.

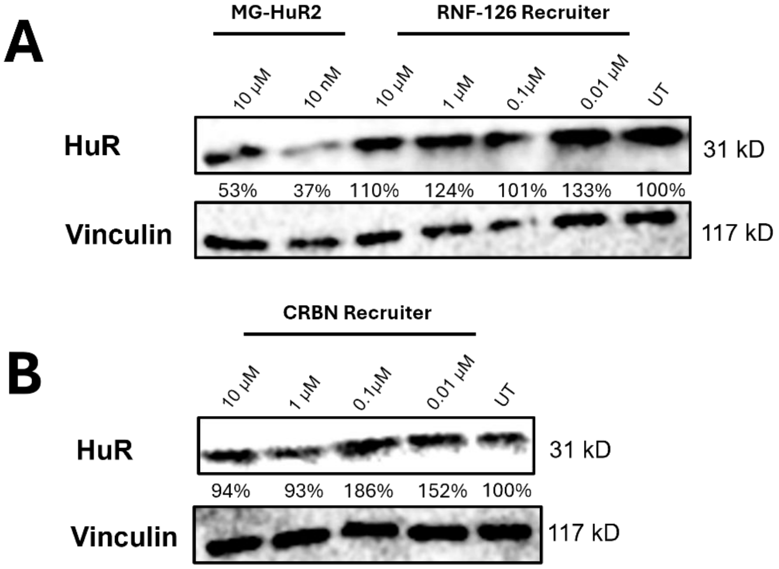

Figure S9. (A) MCF-7 cells were treated with MG-HuR2 in two doses and RNF126 recruiter in a dose dependent manner for 24 h. (B) MCF-7 cells were treated with CRBN recruiter in a dose dependent manner for 48 h.

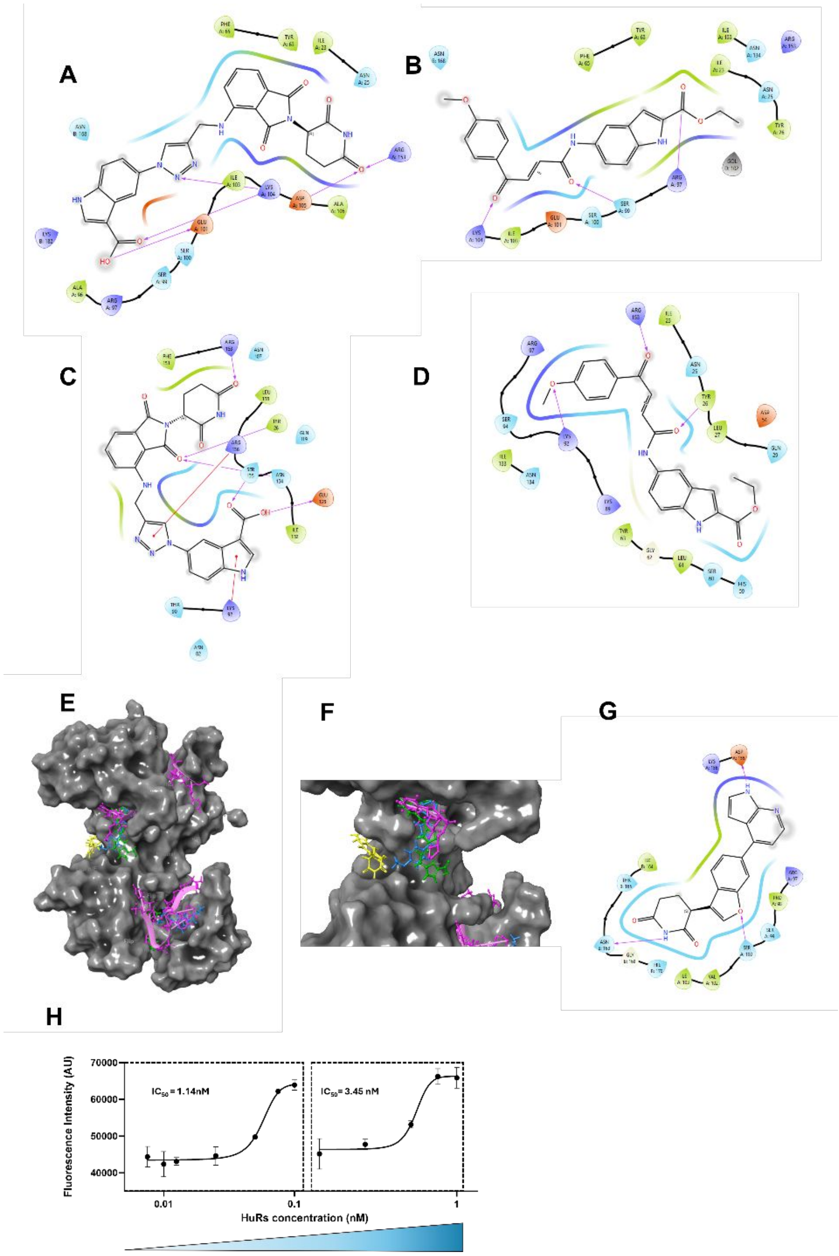

Figure S10. 2D representation of (A) PRO-HuR3 in complex with P1 binding pocket (B) MG-HuR2 in complex with P1 binding pocket C) PRO-HuR3 in complex with P2 binding pocket (D) MG-HuR2 in complex with P2 binding pocket. Hydrogen bonds, π-π stacking and halogen bonds are shown, respectively, as magenta, green and sand lines. The 2D representation was built after the MM-GBSA post-docking analysis. (E-F) 3D representation of HuR in complex with MG-HuR2 (blue) and PRO-HuR3 (green) in both RNA binding pockets, mRNA in P1 and P2 and the commercially available molecular glue (Yellow), obtained after MM-GBSA docking analysis. Atomic coordinates were obtained from the PDB model 4ED5. (G) Commercially available molecular glue in complex

near P1 binding pocket. (H) Binding affinity of 100 μM PRO-HuR3 to HuR measured by florescence intensity.

	Binding pocket P1	Binding pocket P2
MG-HuR2	-46.76 (Kcal/mole)	-39.04 (Kcal/mole)
PRO-HuR3	-44.55 (Kcal/mole)	-41.06 (Kcal/mole)
Commercial molecular glue	–26.8 (Kcal/mole)	-

Table S3. MM-GBSA score for the two RNA-Binding pockets

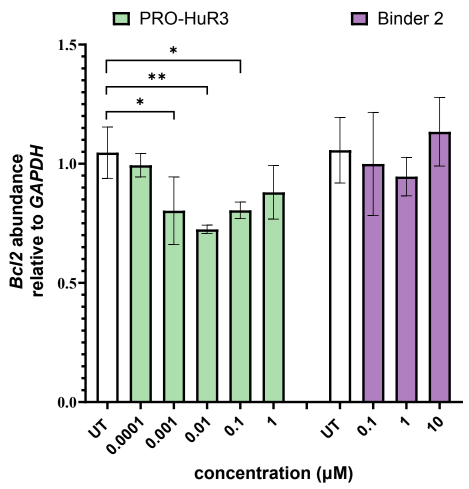

Figure S11. Abundance of *Bcl2* in MCF-7 cells treated dose-dependently with PRO-HuR3 and Binder 2 for 48 h, measured by RT-qPCR. Data is presented as the mean ± SD (n=9), * represents p≤0.05, ** represents p≤0.01, as determined by a one-way ANOVA comparison relative to UT.

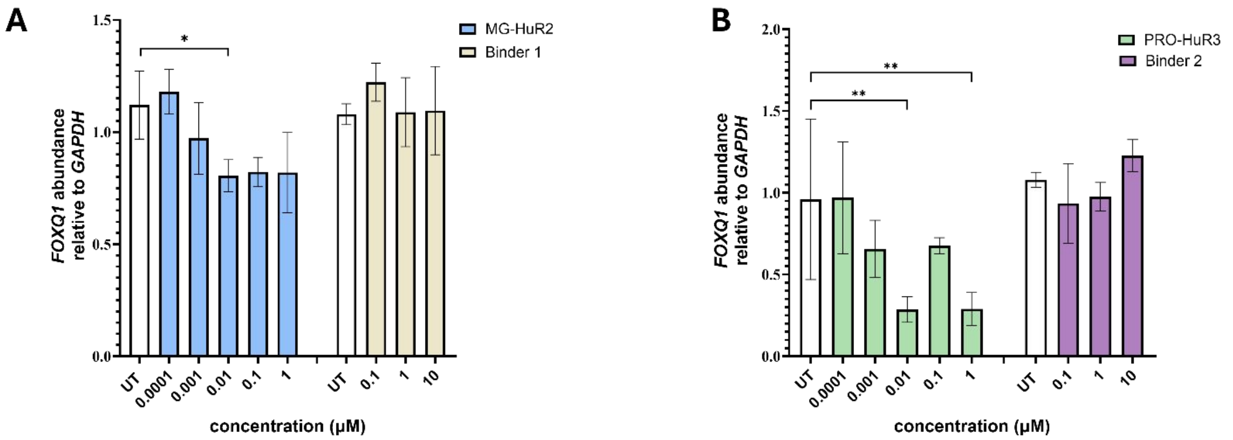

Figure S12. (A) Abundance of *FOXQ1* in MCF-7 cells treated dose-dependently with MG-HuR2 and Binder 1 for 24 h, measured by RT-qPCR. (B) Abundance of *FOXQ1* in MCF-7 cells treated dose-dependently with PRO-HuR4 and Binder 2 for 48 h, measured by RT-qPCR. Data is presented as the mean ± SD (n=9), * represents p≤0.05, ** represents p≤0.01, as determined by a one-way ANOVA comparison relative to UT.

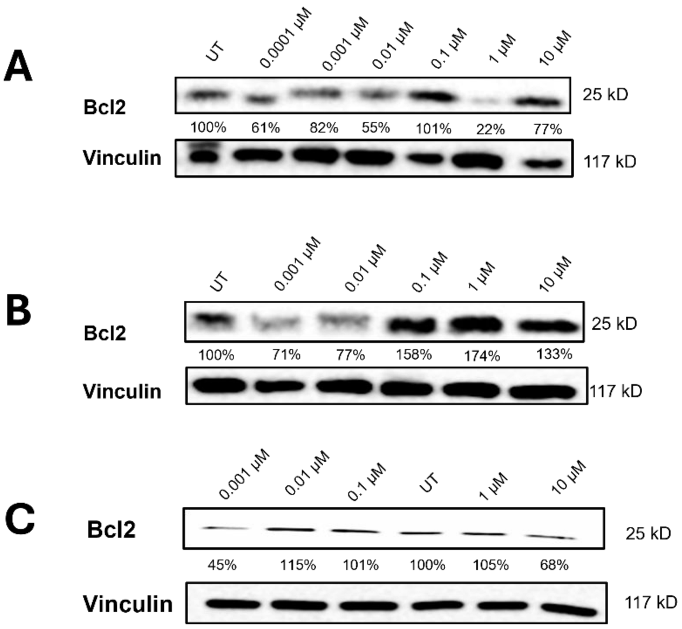

Figure S13. (A) Dose-dependent analysis of Bcl2 after treatment with MG-HuR2 in MDA-MB-231 cells for 24 h. (B) Dose-dependent analysis of Bcl2 after treatment with PRO-HuR3 in MBA-MB-231 cells for 48 h. (C) Dose-dependent analysis of Bcl2 after treatment with PRO-HuR3 in MCF-7 cells for 48 h.

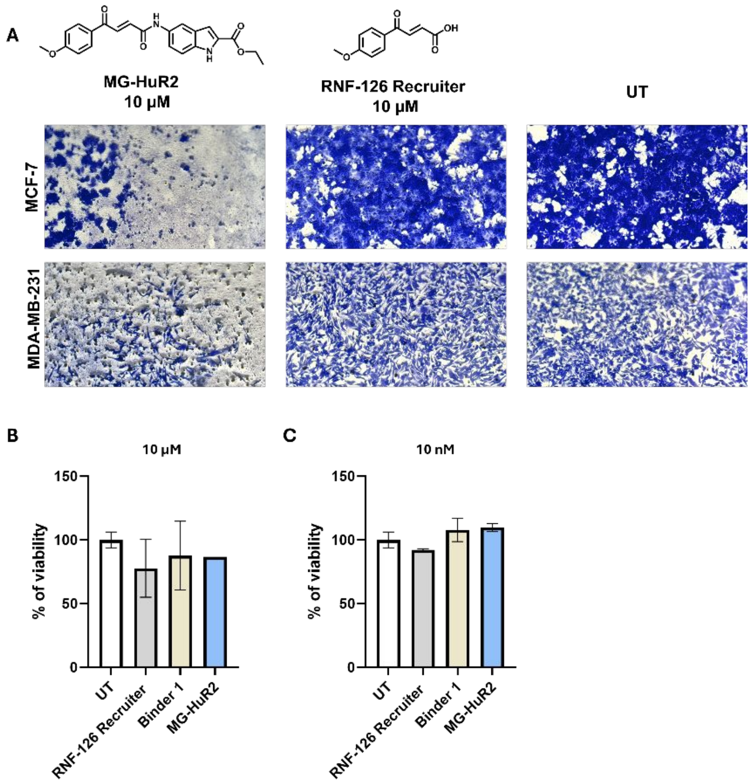

Figure S14. Viability assay of **MCF-7** cells treated with a single dose treatment of MG-HuR2 and RNF-126 recruiter for 48 h, measured using crystal violet. (B) Viability assay of **MCF-10A** cells treated with 10 µM of MG-HuR2, Binder 1 and RNF-126 recruiter for 48 h, measured using crystal violet. (C) Viability assay of **MCF-10A** cells treated with 10 nM of MG-HuR2, Binder 1 and RNF-126 recruiter for 48 h, measured using crystal violet.

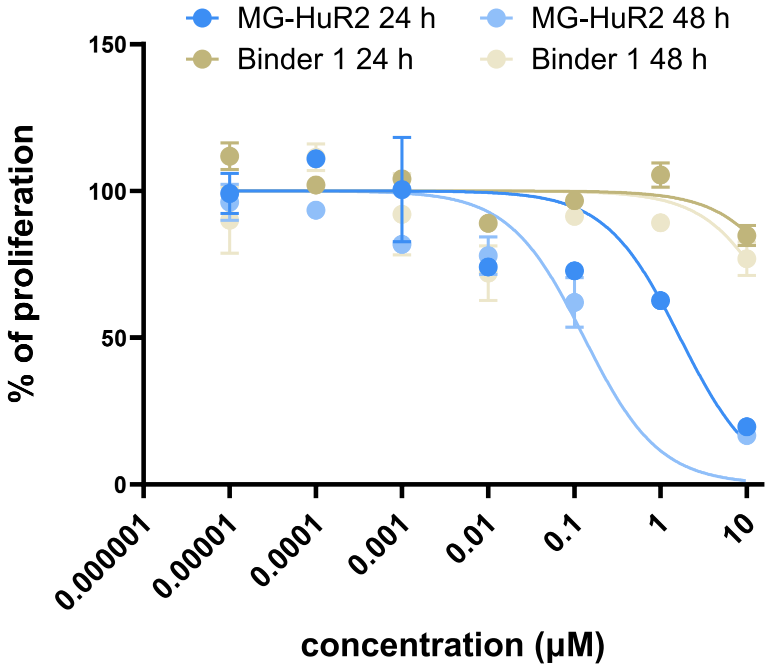

Figure S15. Dose-response experiment for MG-HuR2 for 24 and 48 h in MDA-MB-231 cells. Data is presented as the mean ± SD (n=3), curve was extrapolated via inhibitor vs. normalized response-fitting using Prism 10.2.2. The experiments were performed in triplicate.

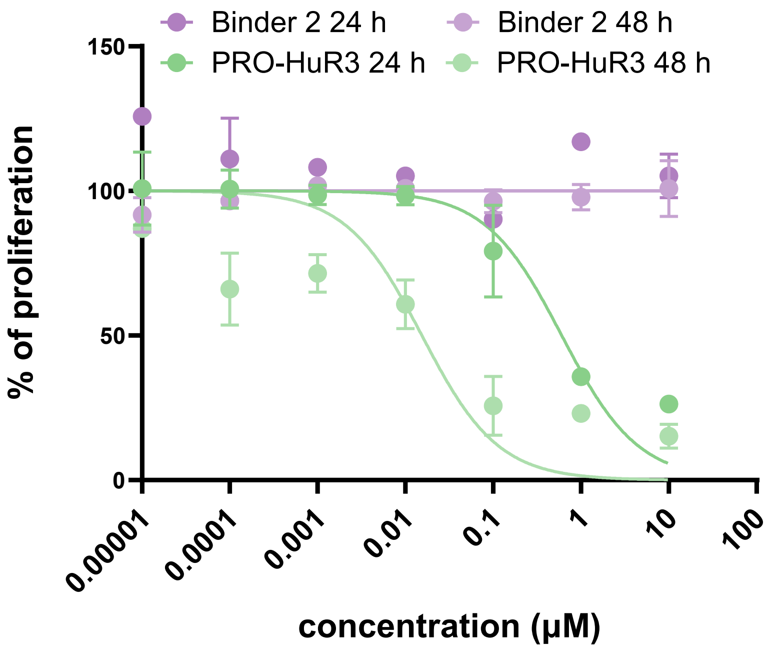

Figure S16. Dose-response experiment for PRO-HuR3 for 24 and 48 h in MCF-7 cells. Data is presented as the mean ± SD (n=3), curve was extrapolated via inhibitor vs. normalized response-fitting using Prism 10.2.2. The experiments were performed in triplicate.

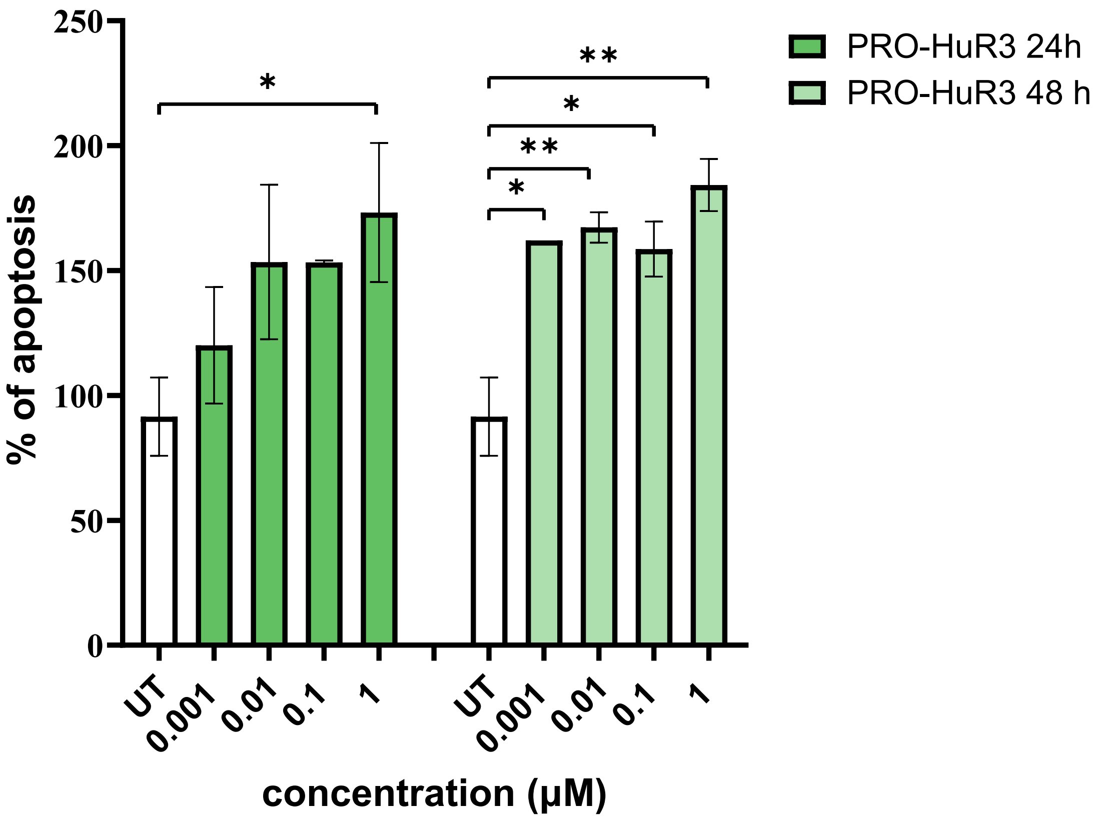

Figure S17. Dose-response experiment for PRO-HuR3 for 24 and 48 h in MCF-7 cells. Data is presented as the mean ± SD (n=3), * represents p≤0.05, ** represents p≤0.01as determined by a one-way ANOVA comparison relative to UT.

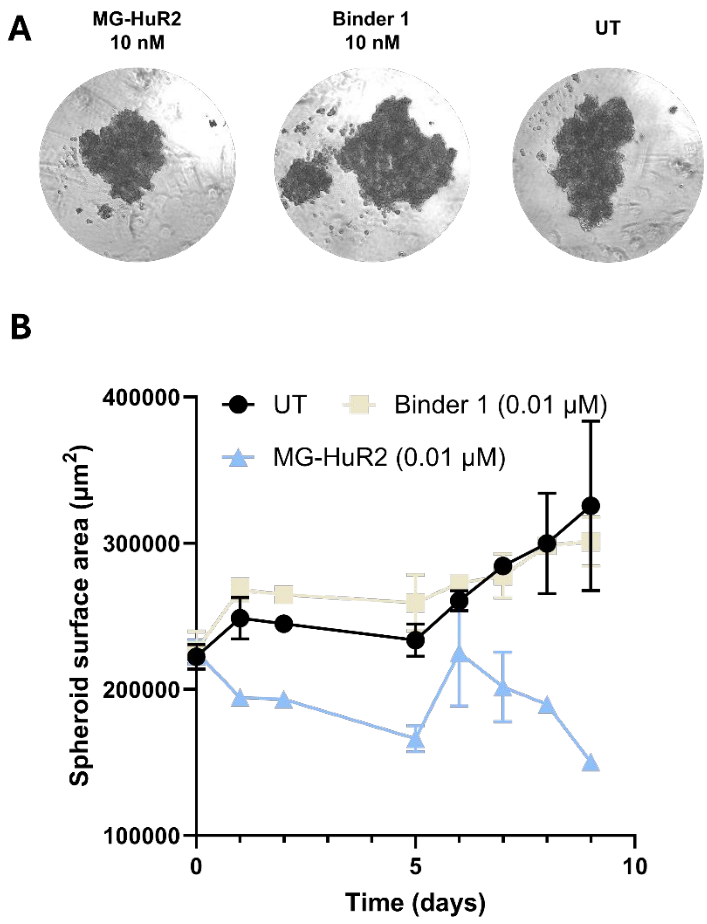

Figure S18. (A) Representative images for MDA-MB-231 spheroids growth, day 12 after treatment (B) MDA-MB-231 Spheroids surface area growth after treatment with a single dose of MG-HuR2 and Binder 1 Data is presented as the mean ± SD (n=2) .

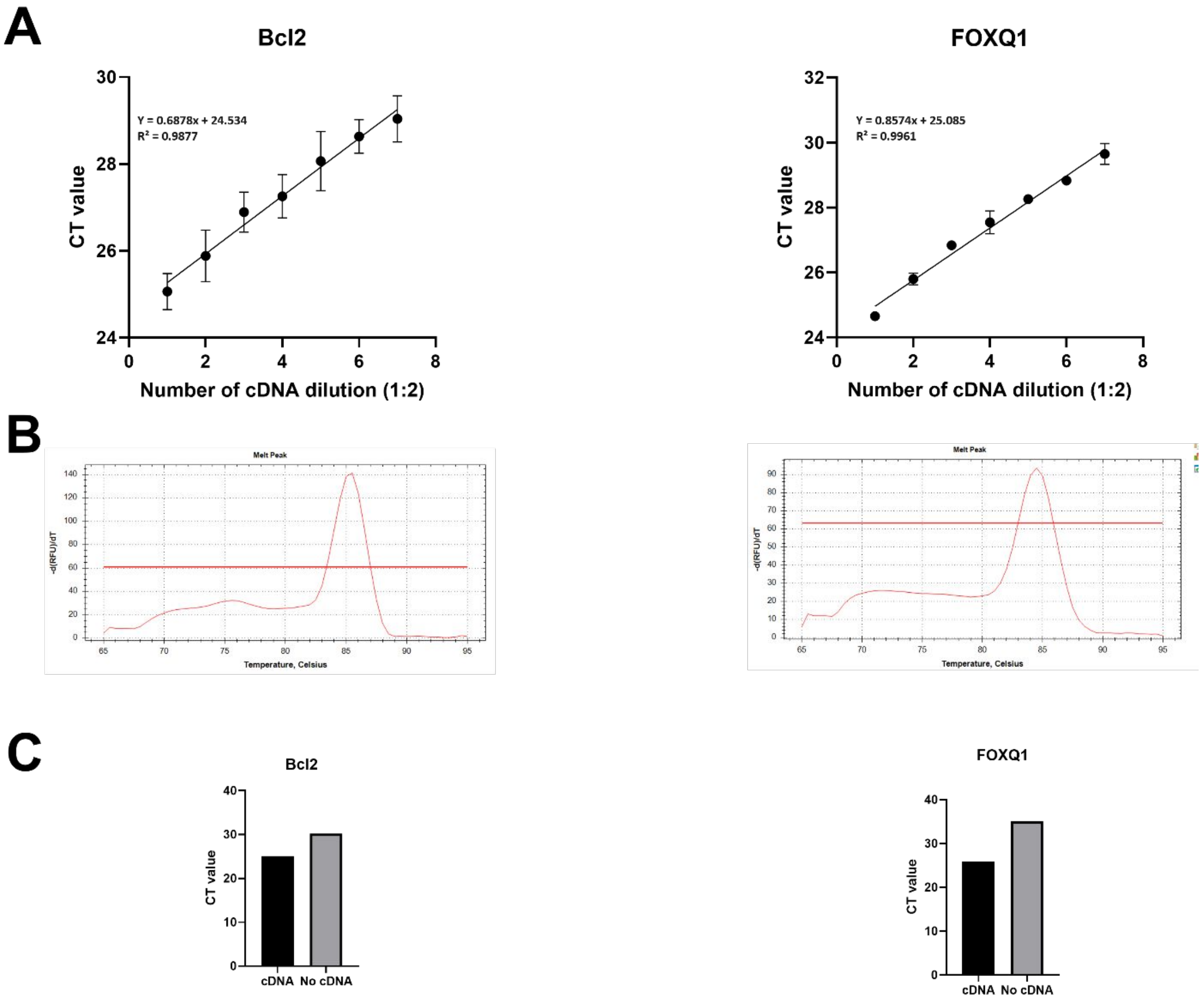

Figure S19. Validation of RT-qPCR primers used to measure levels of Bcl2 and FOXQ1 genes by RT-qPCR. (A) Ct values as a function of cDNA dilutions from reverse transcription. (B) Melting curves support that only one product is amplified for each gene. (C) No template control does not amplify by RT-qPCR (Ct values > 35).

Figure S20. Characterization of compound 2

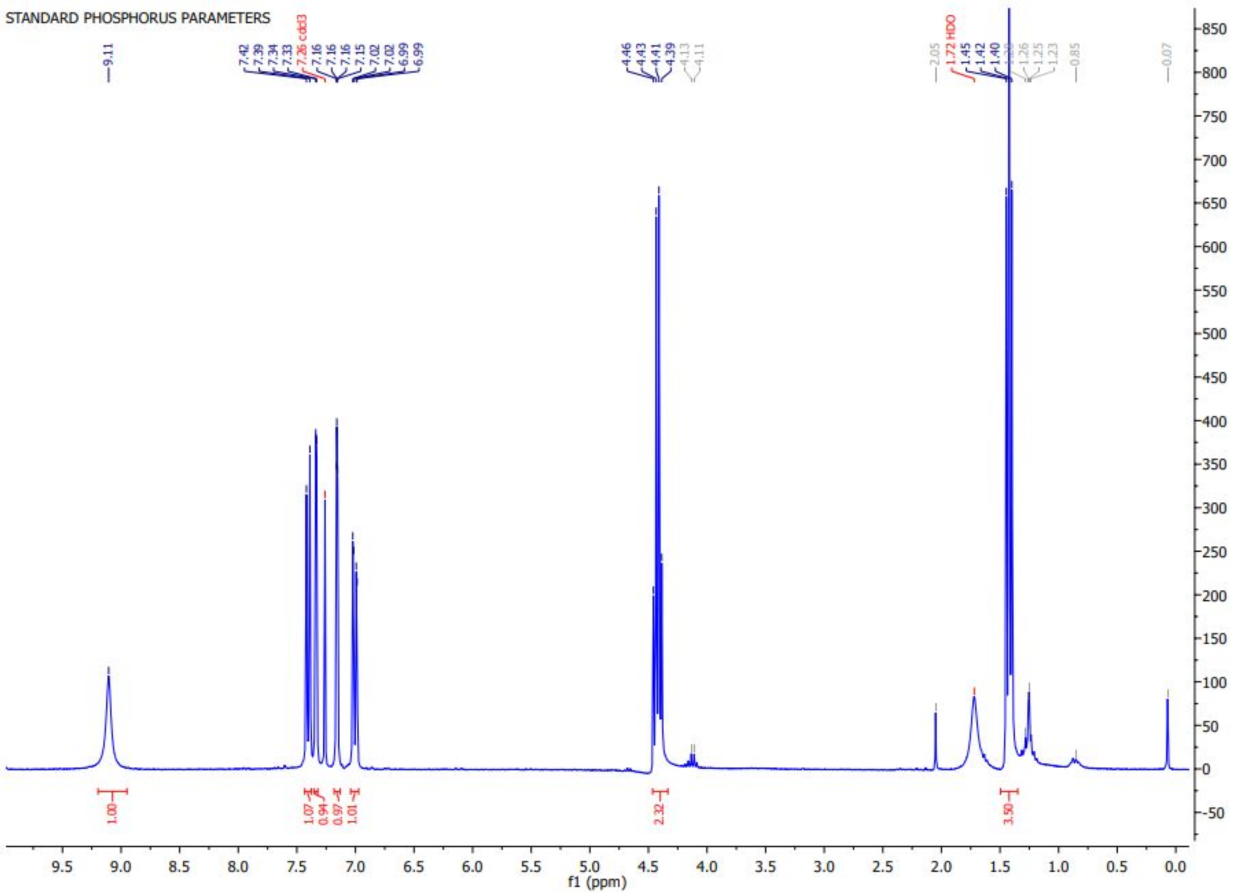

Figure S21. Characterization of compound 5

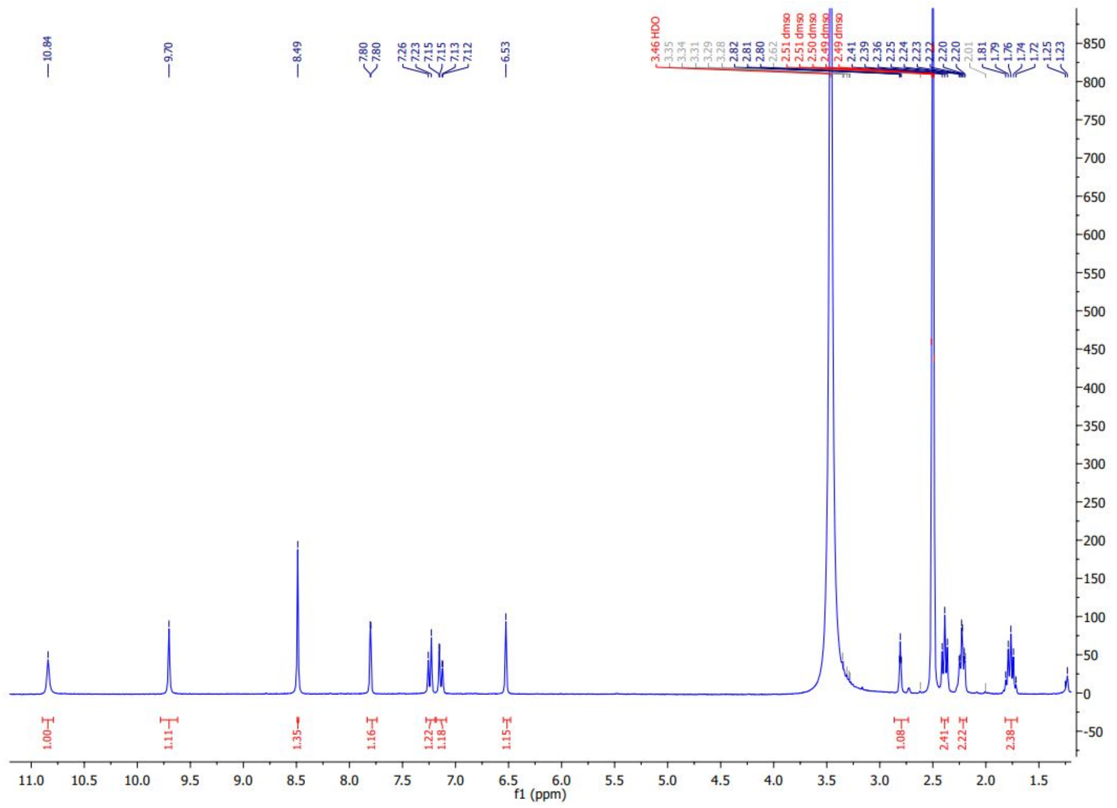

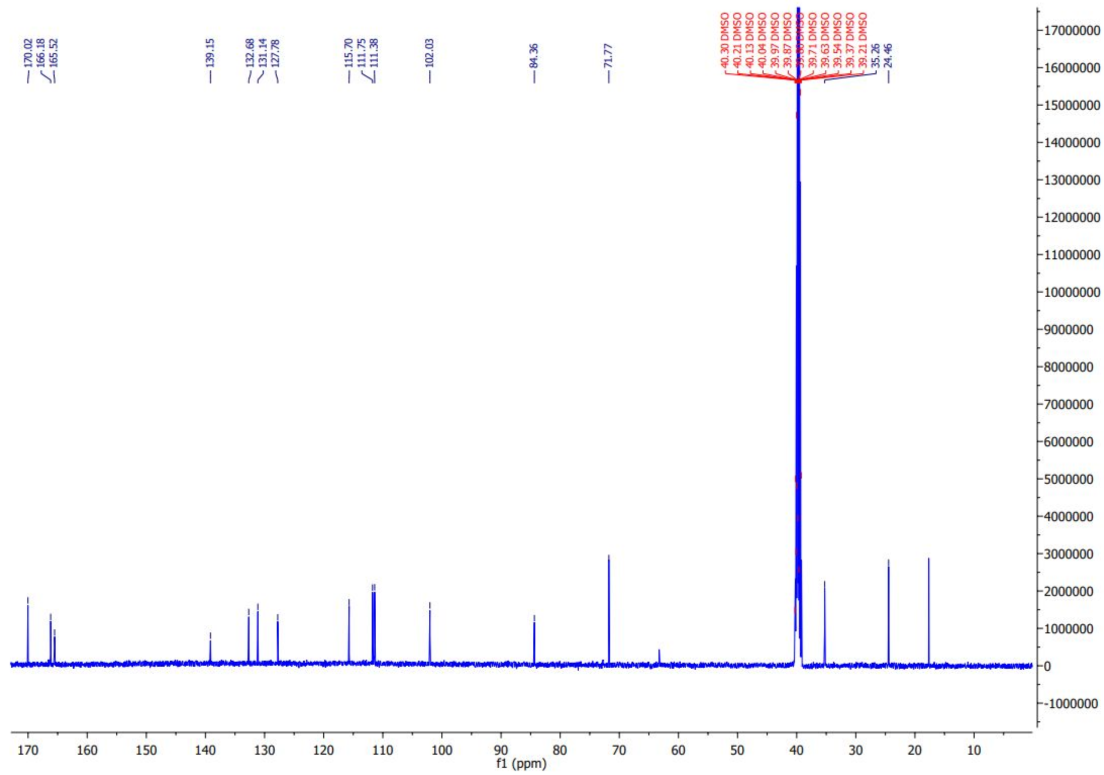

Figure S22. Characterization of compound 7

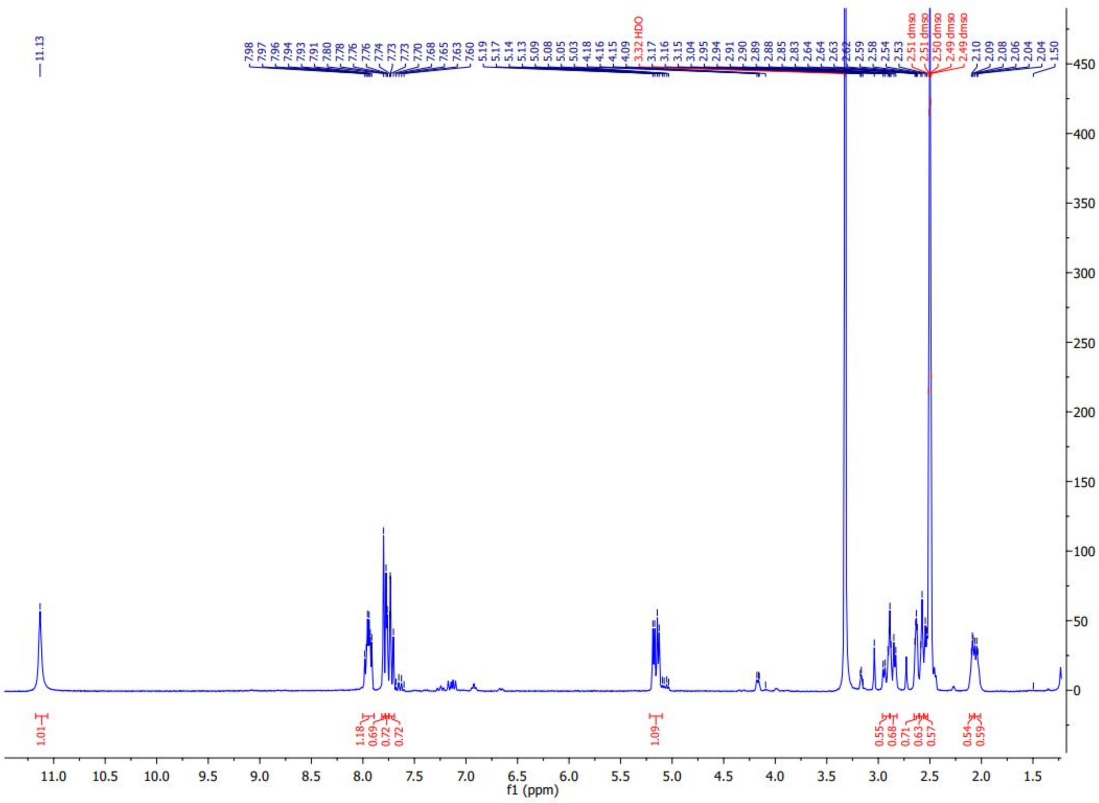

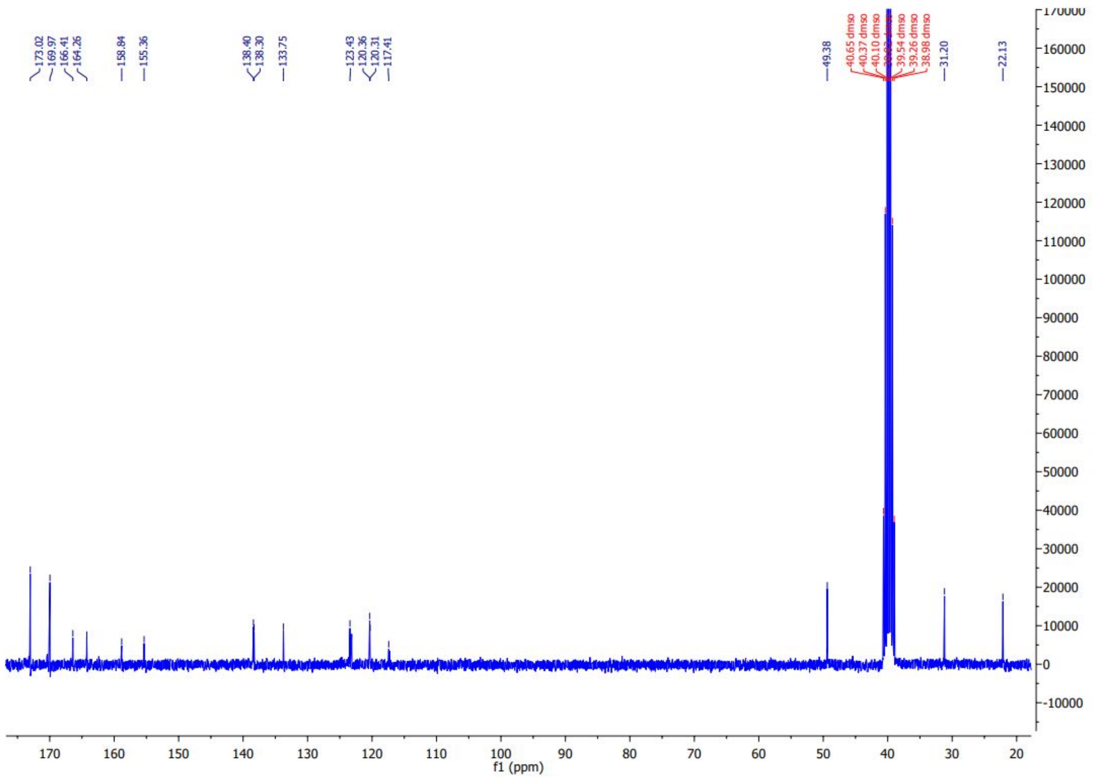

Figure S23. Characterization of compound 8

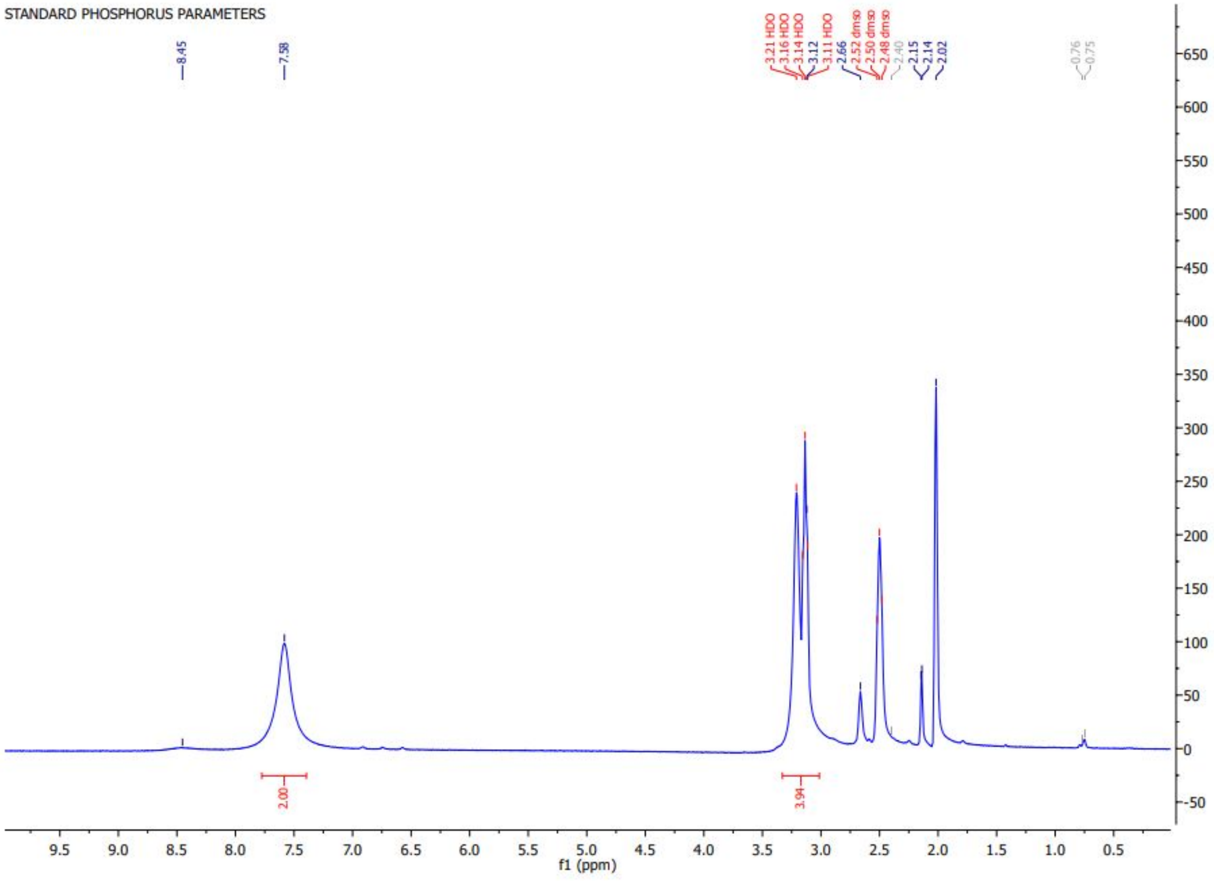

Figure S24. Characterization of compound 11

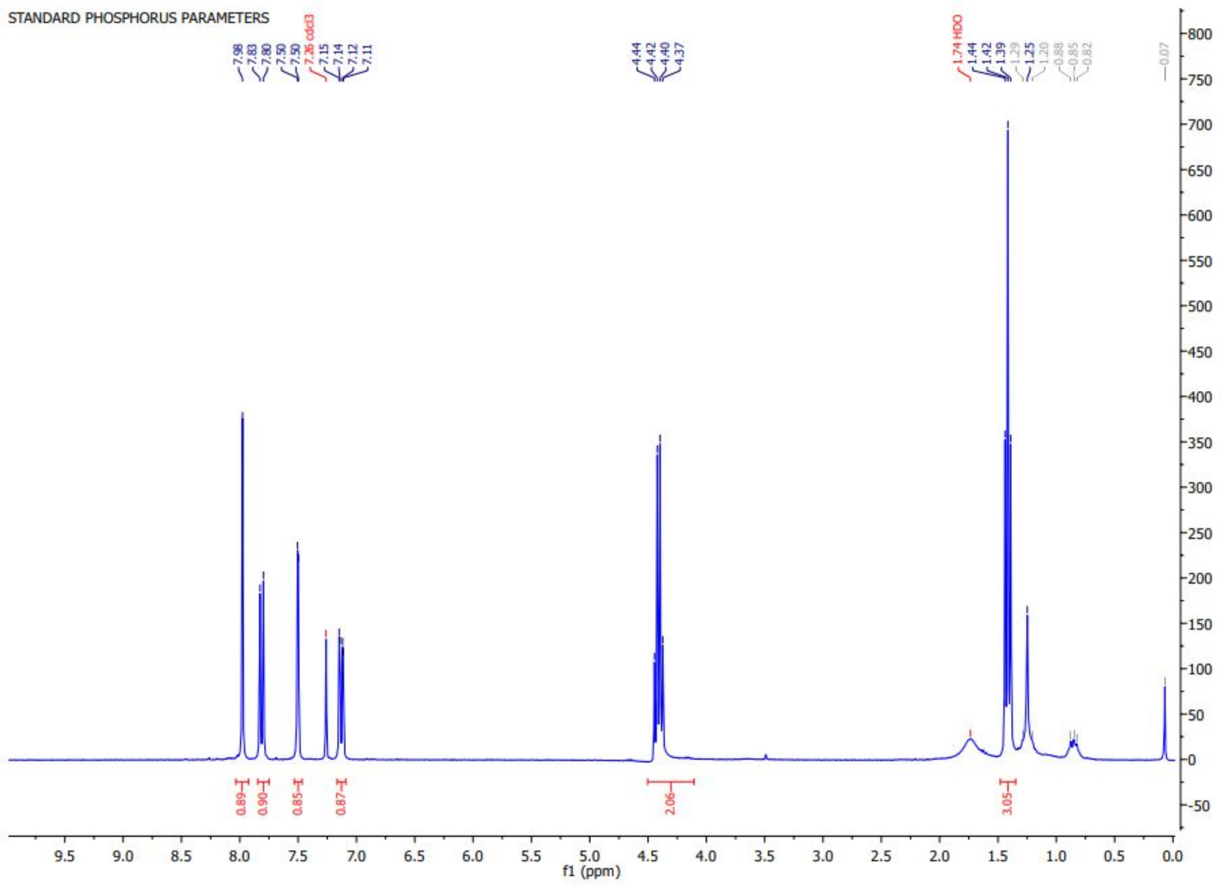

Figure S25. Characterization of compound 12

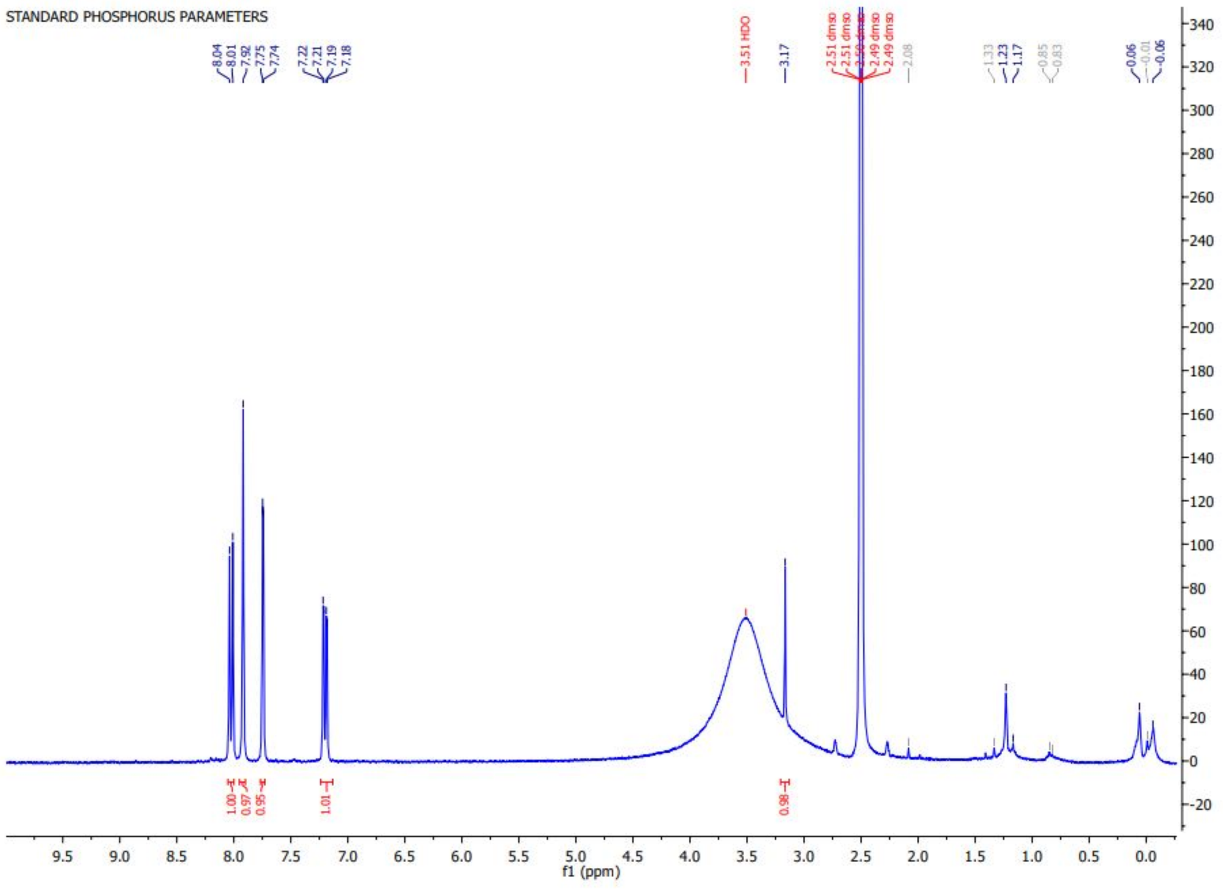

Figure S26. Characterization of compound 16

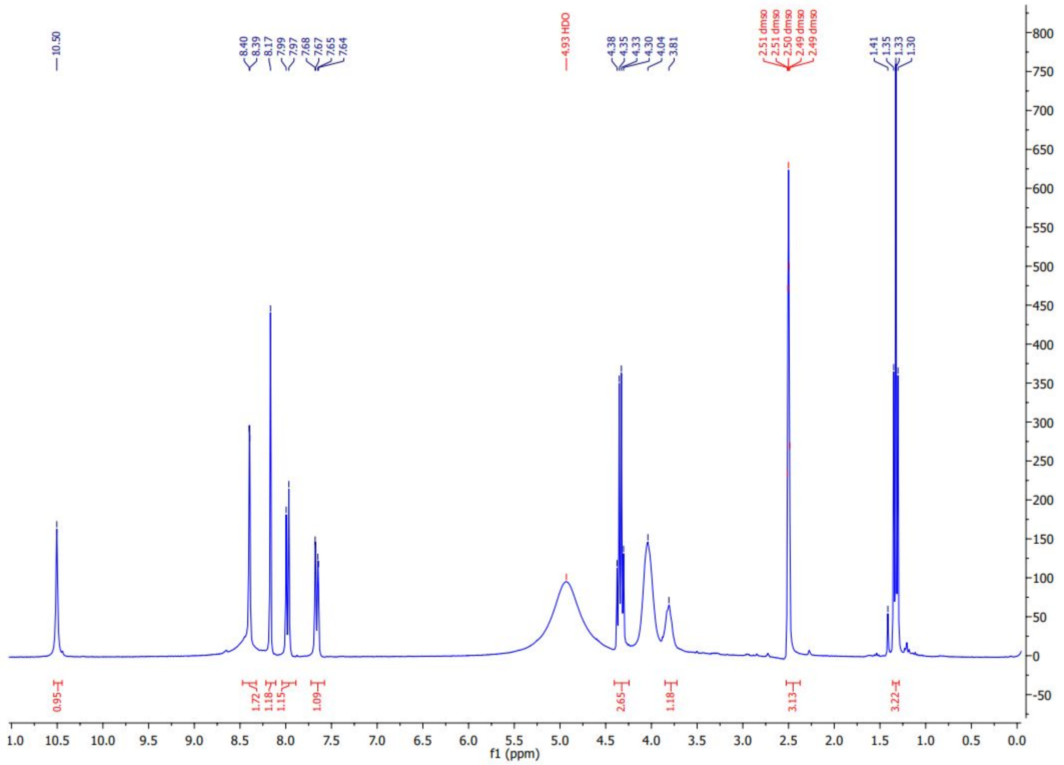

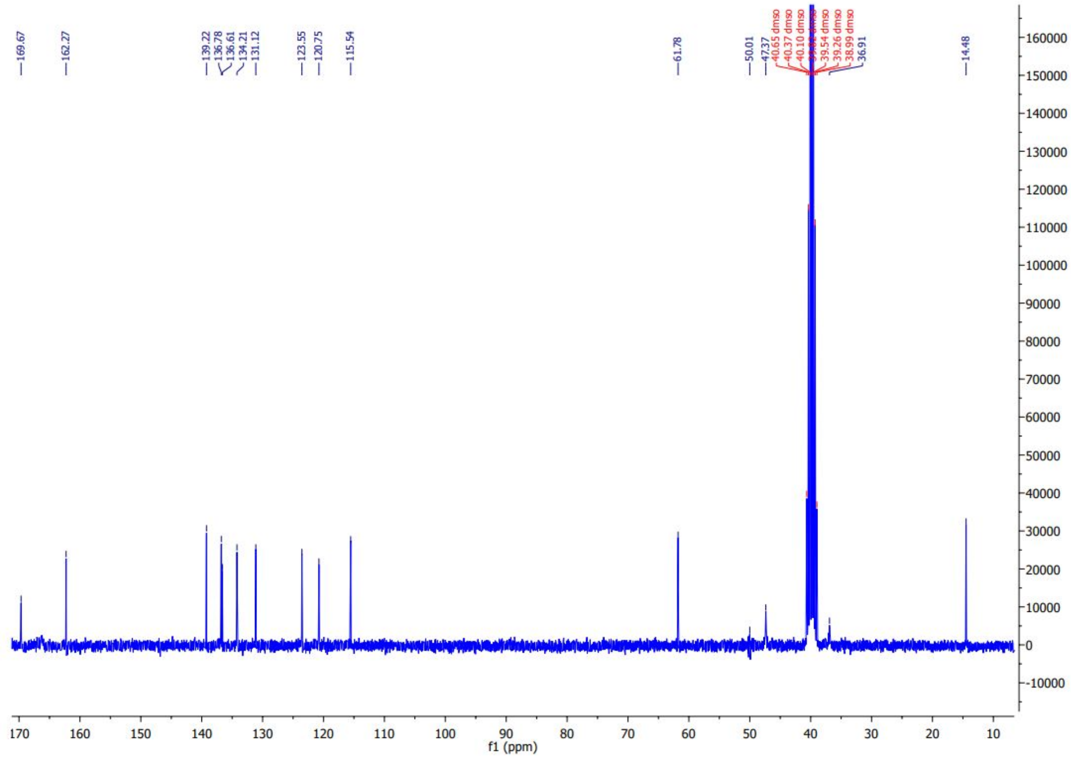

Figure S27. Characterization of PRO-HuR1

Analytical HPLC:

<Chromatogram>

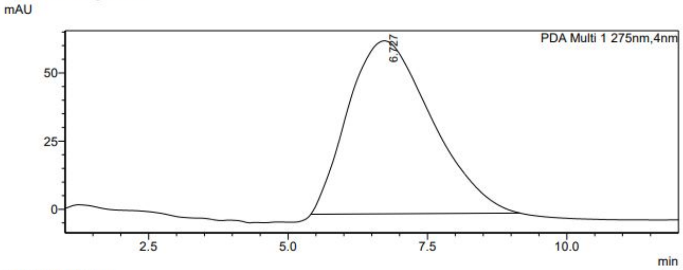

<Peak Table>

PDA Ch1 275nm

Peak#	Ret. Time	Area	Height	Area%
1	6.727	6620101	63405	100.000
Total		6620101	63405	100.000

NMR:

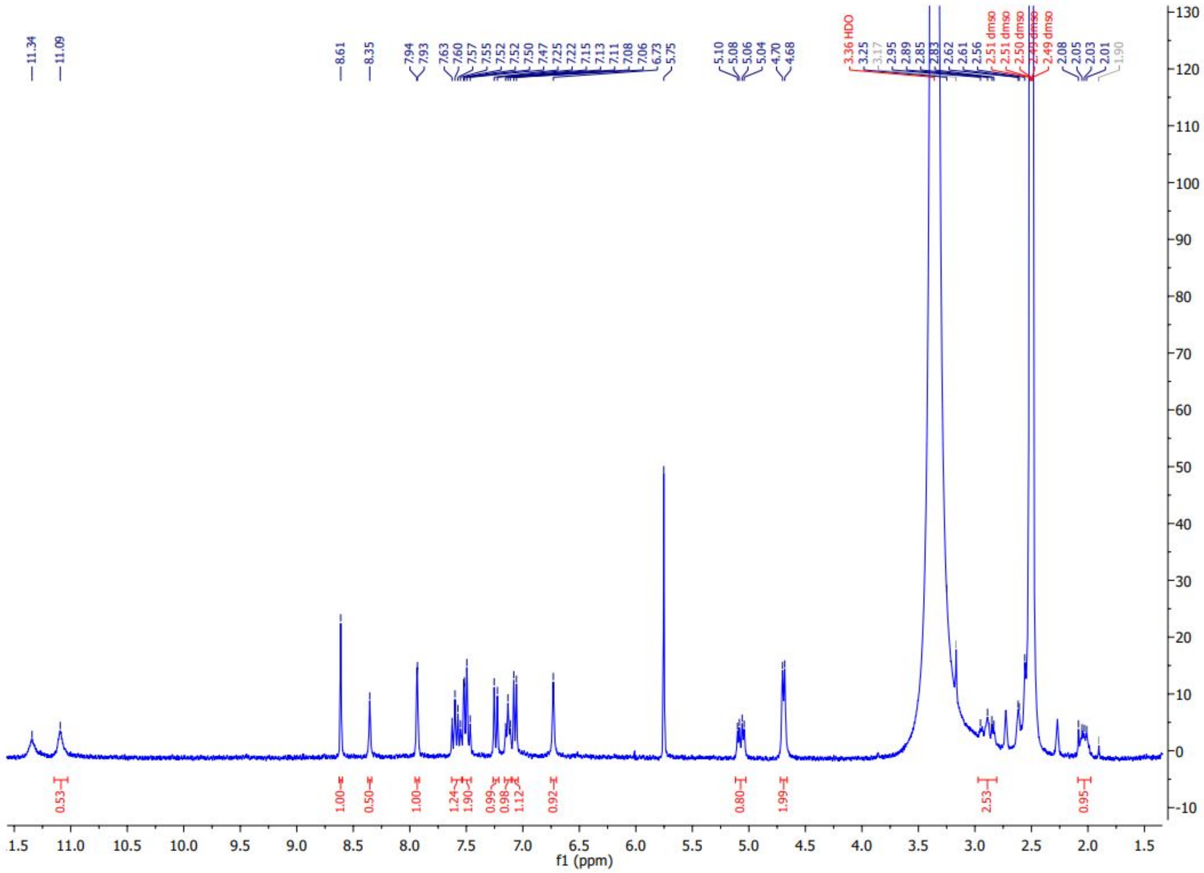

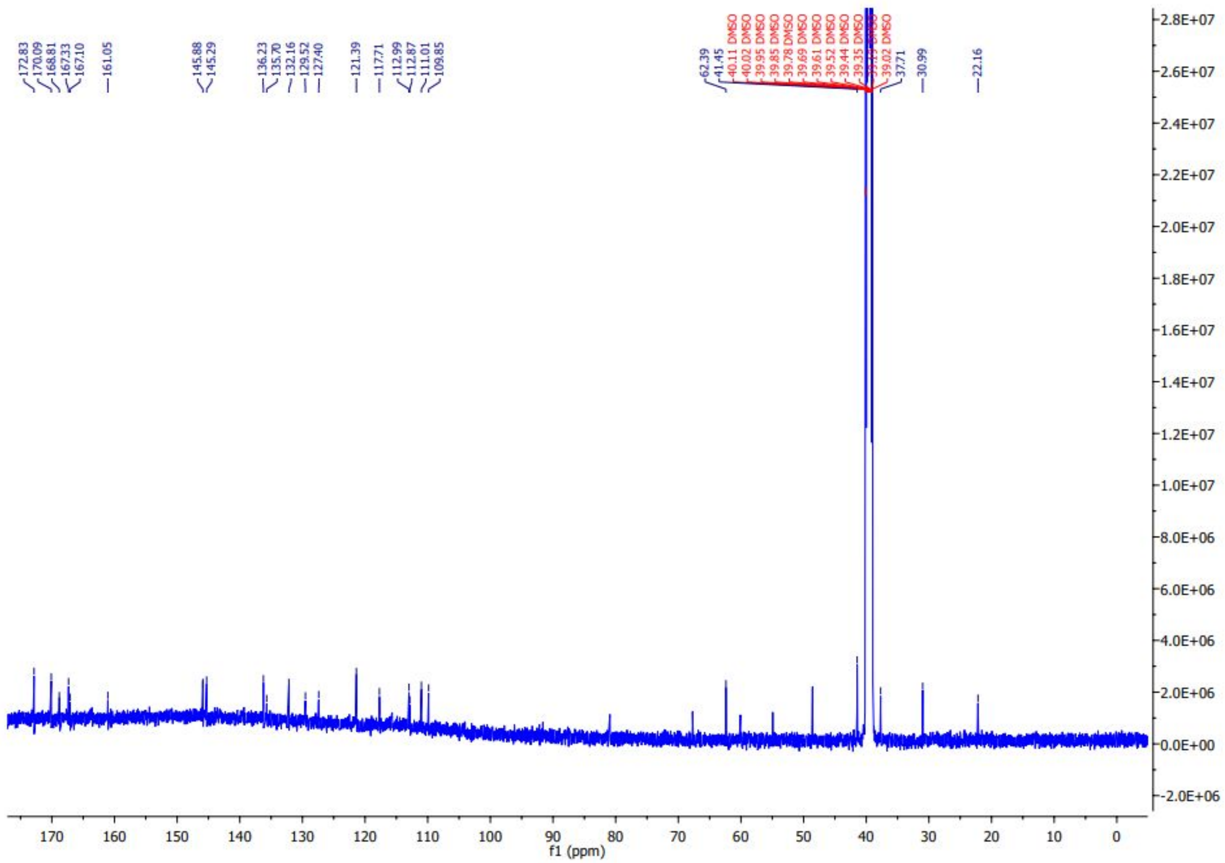

172.83
170.09
168.81
167.33
167.10
161.05
145.88
145.29
136.23
135.70
132.16
129.52
127.40
121.39
117.71
112.99
112.87
111.01
109.85
62.39
41.45
40.11 DMSO
40.02 DMSO
39.95 DMSO
39.85 DMSO
39.78 DMSO
39.69 DMSO
39.61 DMSO
39.52 DMSO
39.44 DMSO
39.35 DMSO
39.19 DMSO
39.02 DMSO
37.71
30.99
22.16
2.8E+07
2.6E+07
2.4E+07
2.2E+07
2.0E+07
1.8E+07
1.6E+07
1.4E+07
1.2E+07
1.0E+07
8.0E+06
6.0E+06
4.0E+06
2.0E+06
0.0E+00
-2.0E+06
170 160 150 140 130 120 110 100 90 80 70 60 50 40 30 20 10 0
f1 (ppm)

Figure S28. Characterization of PRO-HuR2

Analytical HPLC:

<Chromatogram>

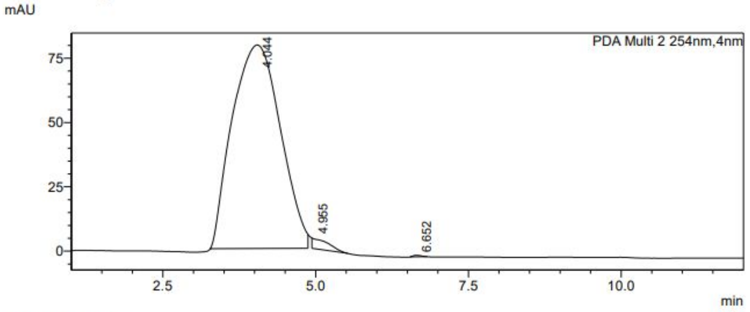

<Peak Table>

PDA Ch2 254nm

Peak#	Ret. Time	Area	Height	Area%
1	4.044	4348152	79132	98.201
2	4.955	74455	3927	1.682
3	6.652	5186	590	0.117
Total		4427793	83650	100.000

NMR:

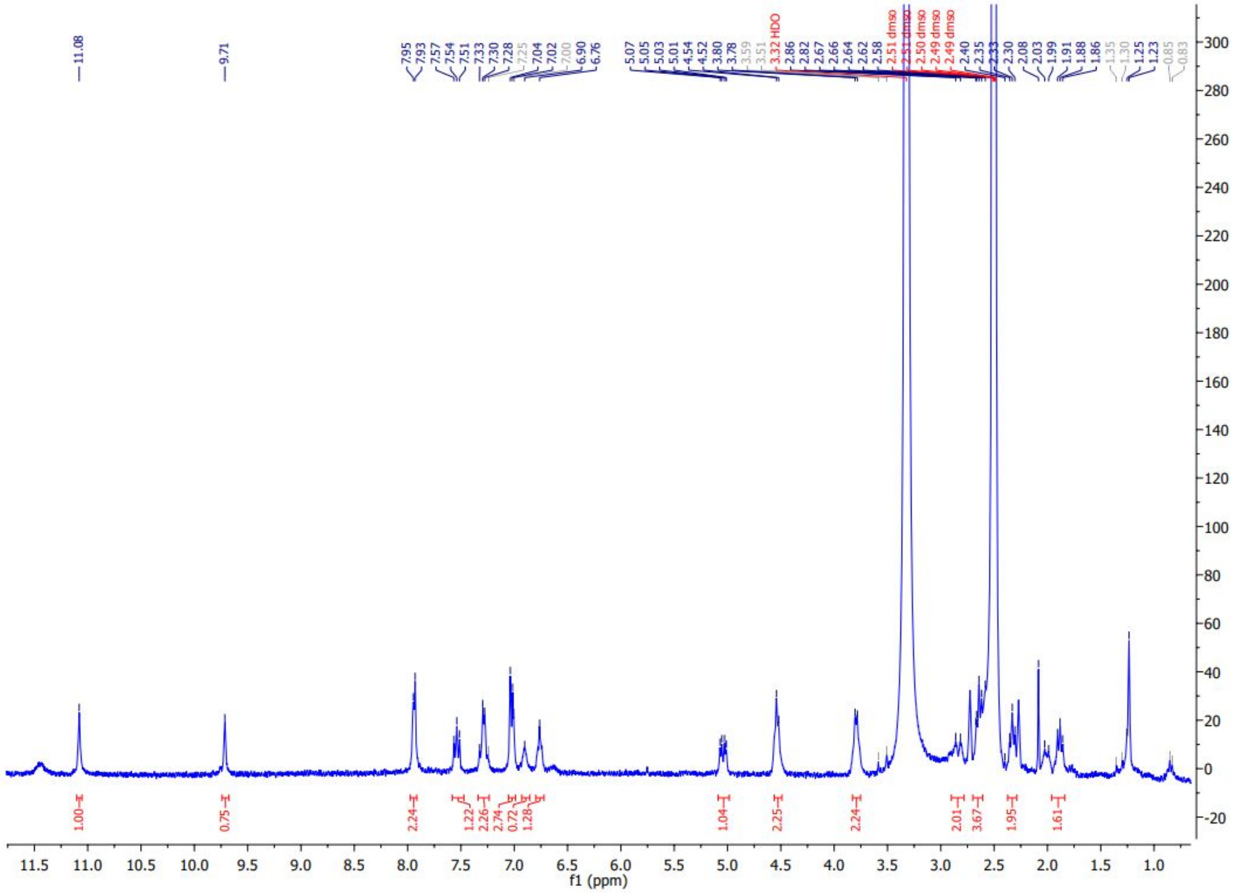

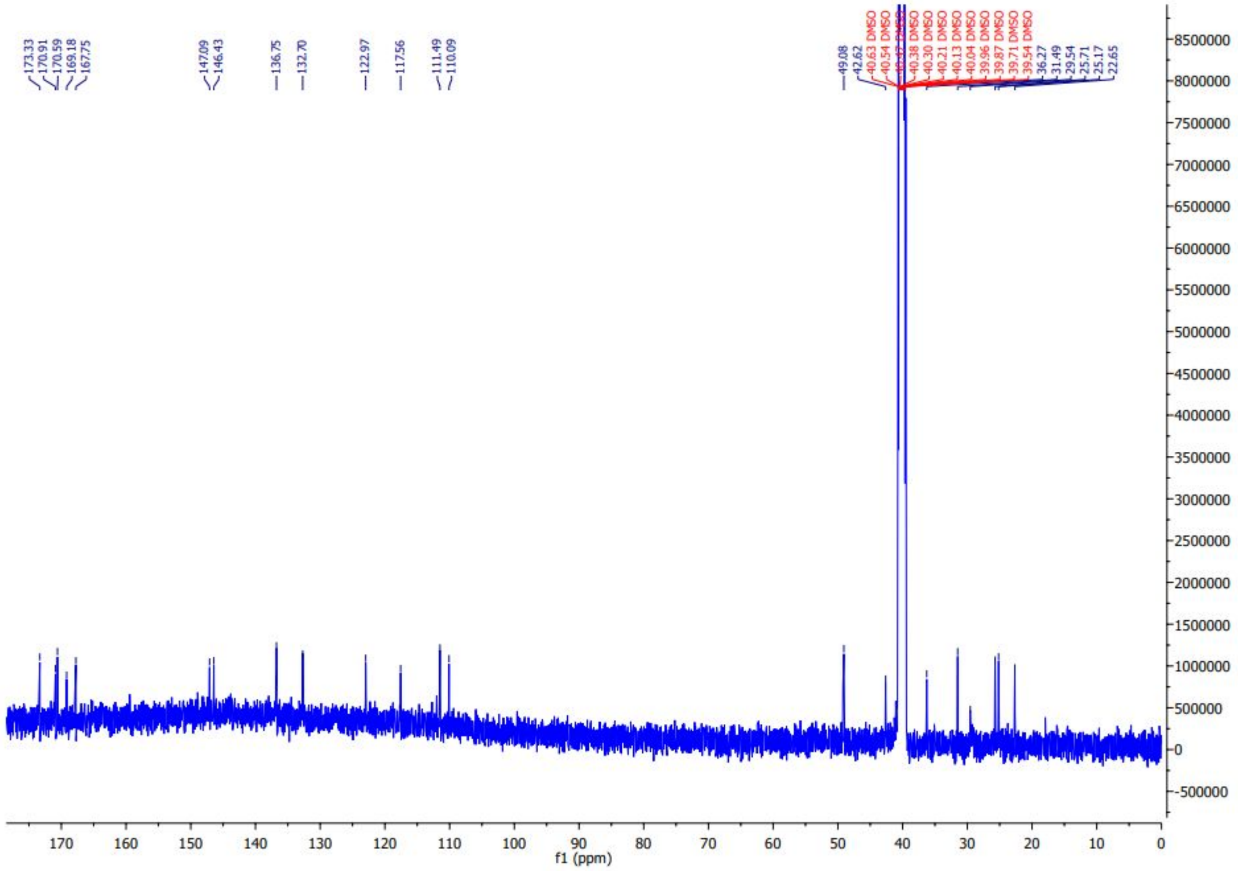
173.33
170.91
170.59
169.18
167.75
147.09
146.43
136.75
132.70
122.97
117.56
111.49
110.09
49.08
42.62
40.63 DMSO
40.54 DMSO
40.38 DMSO
40.30 DMSO
40.21 DMSO
40.13 DMSO
40.04 DMSO
39.96 DMSO
39.87 DMSO
39.71 DMSO
39.54 DMSO
36.27
31.49
29.54
25.71
25.17
22.65
f1 (ppm)

Figure S29. Characterization of MG-HuR1

Analytical HPLC:

<Chromatogram>

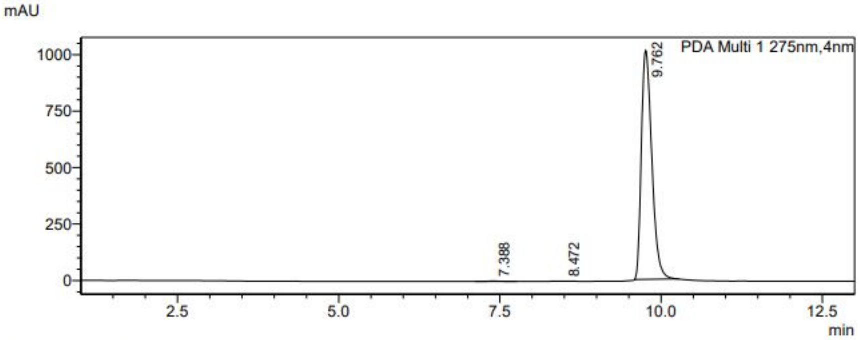

<Peak Table>

PDA Ch1 275nm

Peak#	Ret. Time	Area	Height	Area%
1	7.388	25588	2051	0.223
2	8.472	9013	782	0.079
3	9.762	11431552	1013885	99.698
Total		11466153	1016717	100.000

NMR:

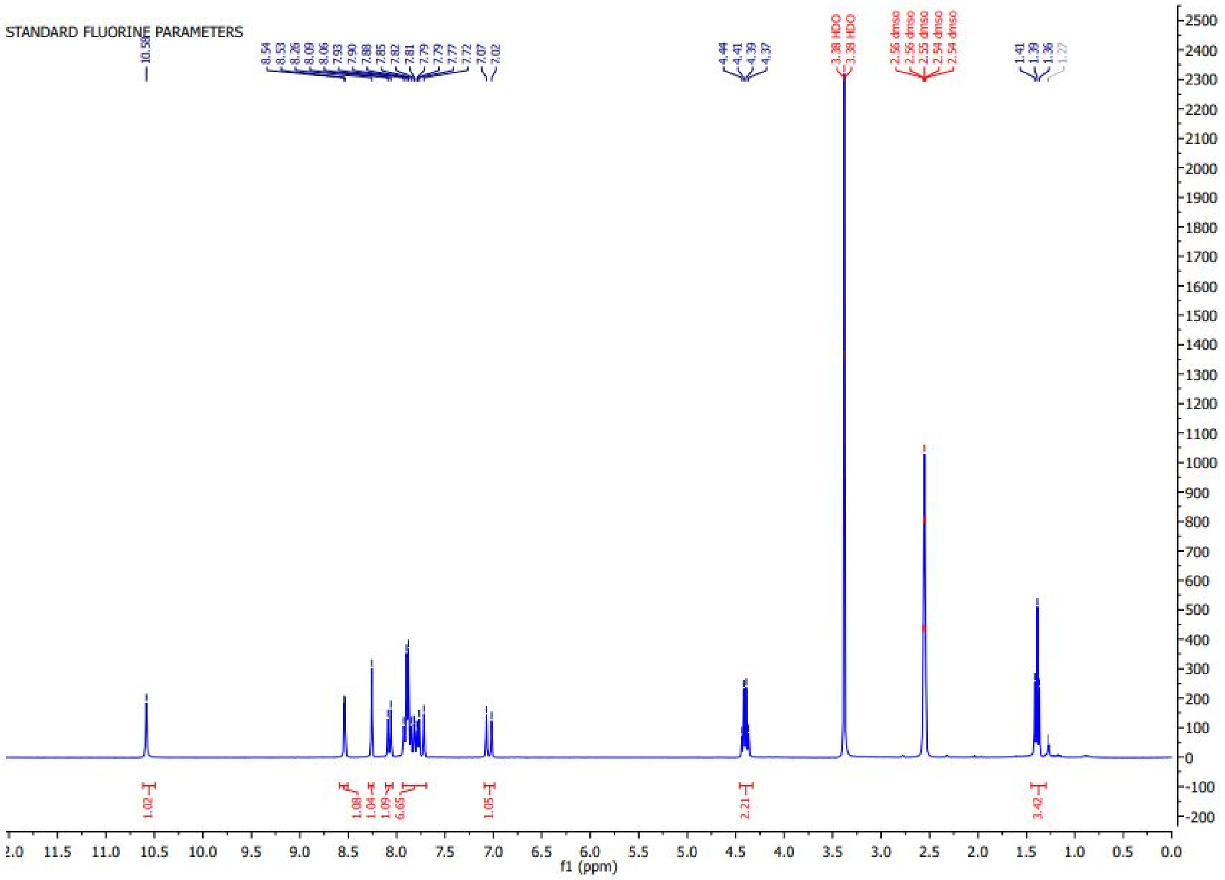

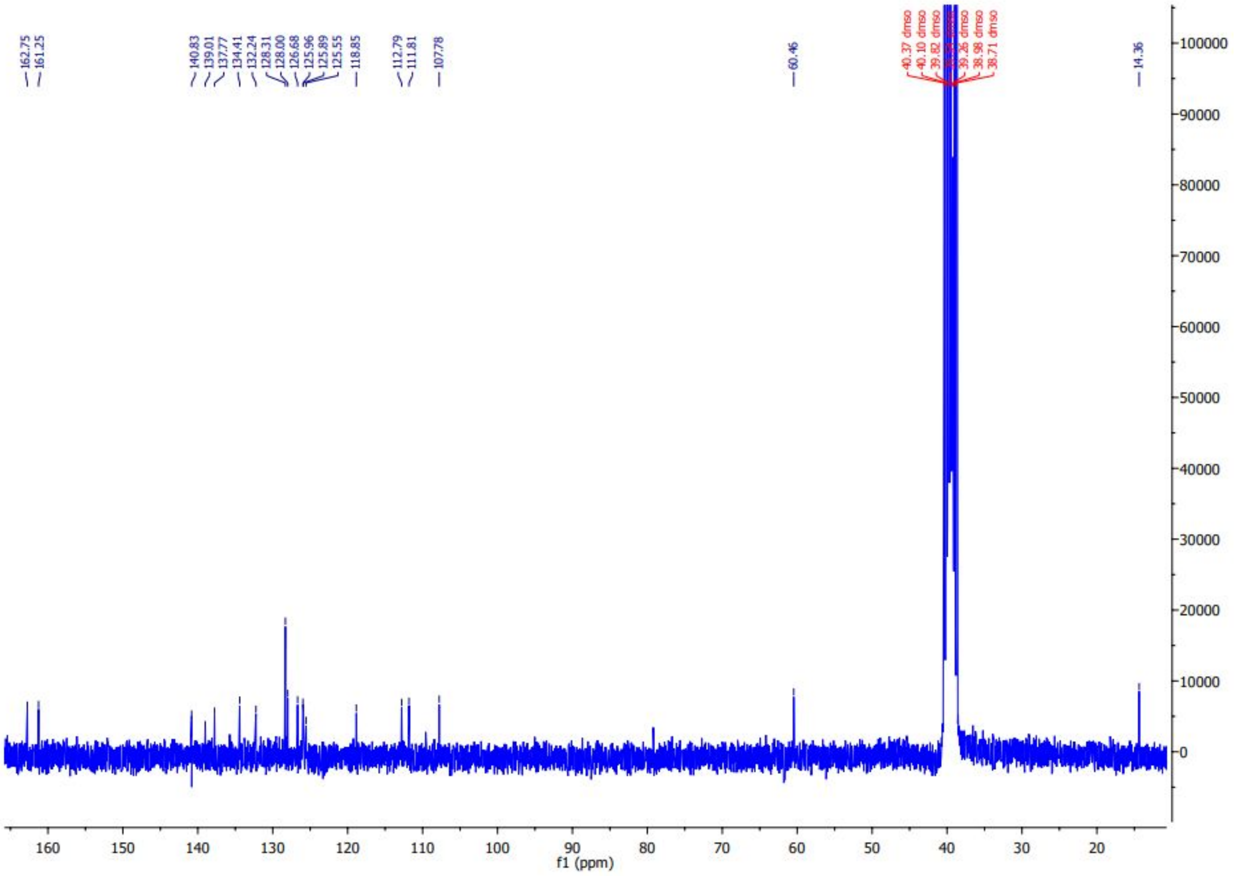
162.75
161.25
140.83
139.01
137.77
134.41
132.24
128.31
128.00
126.68
125.96
125.89
125.55
118.85
112.79
111.81
107.78
60.46
40.37 dmso
40.10 dmso
39.82 dmso
39.26 dmso
38.98 dmso
38.71 dmso
14.36
100000
90000
80000
70000
60000
50000
40000
30000
20000
10000
0
160
150
140
130
120
110
100
90
80
70
60
50
40
30
20
f1 (ppm)

Figure S30. Characterization of MG-HuR2

Analytical HPLC:

<Chromatogram>

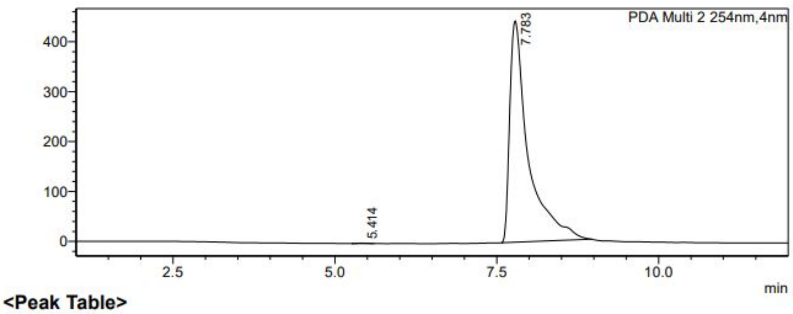

<Peak Table>

PDA Ch2 254nm

Peak#	Ret. Time	Area	Height	Area%
1	5.414	7667	877	0.086
2	7.783	8884576	443104	99.914
Total		8892242	443981	100.000

NMR:

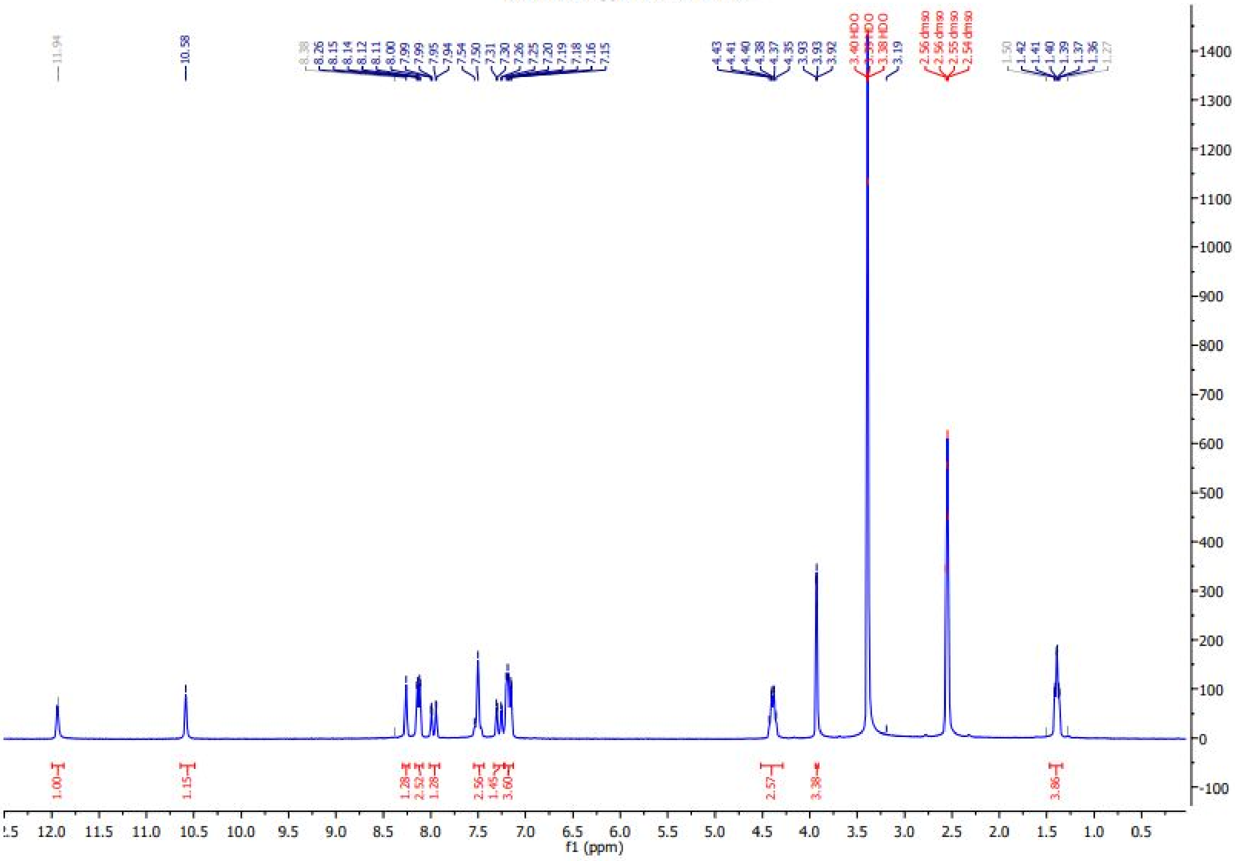

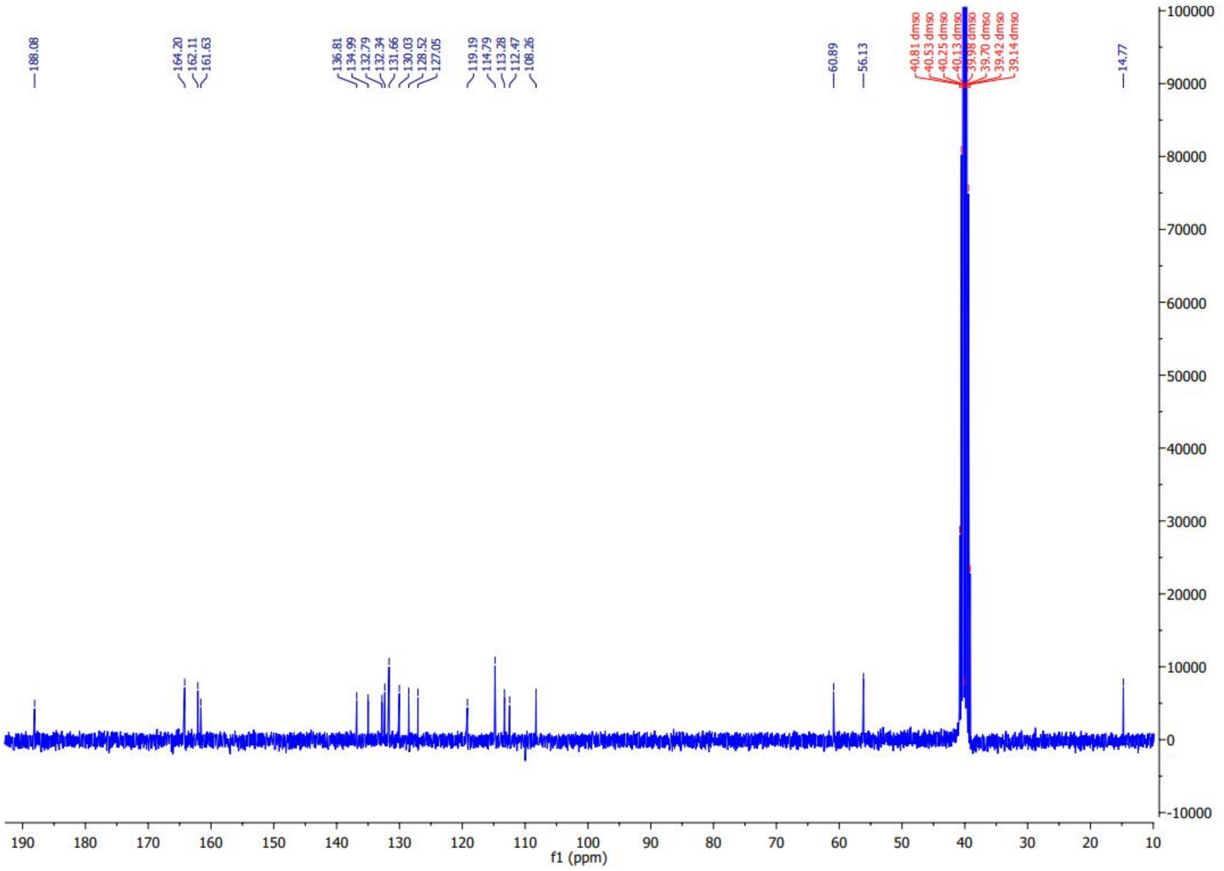
188.08
164.20
162.11
161.63
136.81
134.99
132.79
132.34
131.66
130.03
128.52
127.05
119.19
114.79
113.28
112.47
108.26
60.89
56.13
40.81 dmso
40.53 dmso
40.25 dmso
40.13 dmso
39.98 dmso
39.70 dmso
39.42 dmso
39.14 dmso
14.77
f1 (ppm)

Figure S31. Characterization of MG-HuR3

Analytical HPLC:

<Chromatogram>

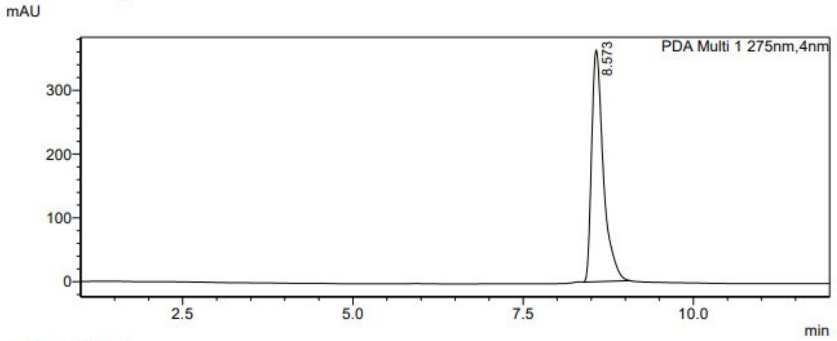

<Peak Table>

PDA Ch1 275nm

Peak#	Ret. Time	Area	Height	Area%
1	8.573	4352972	362572	100.000
Total		4352972	362572	100.000

NMR:

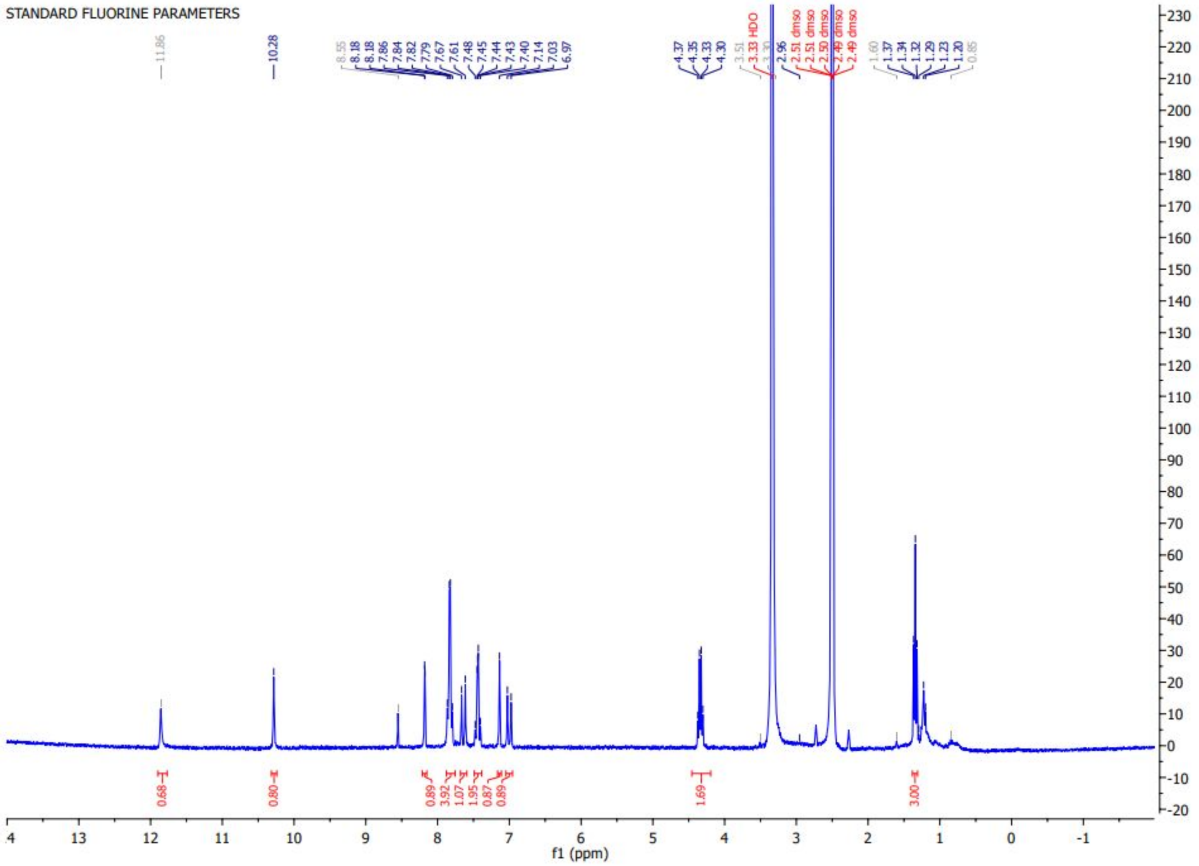

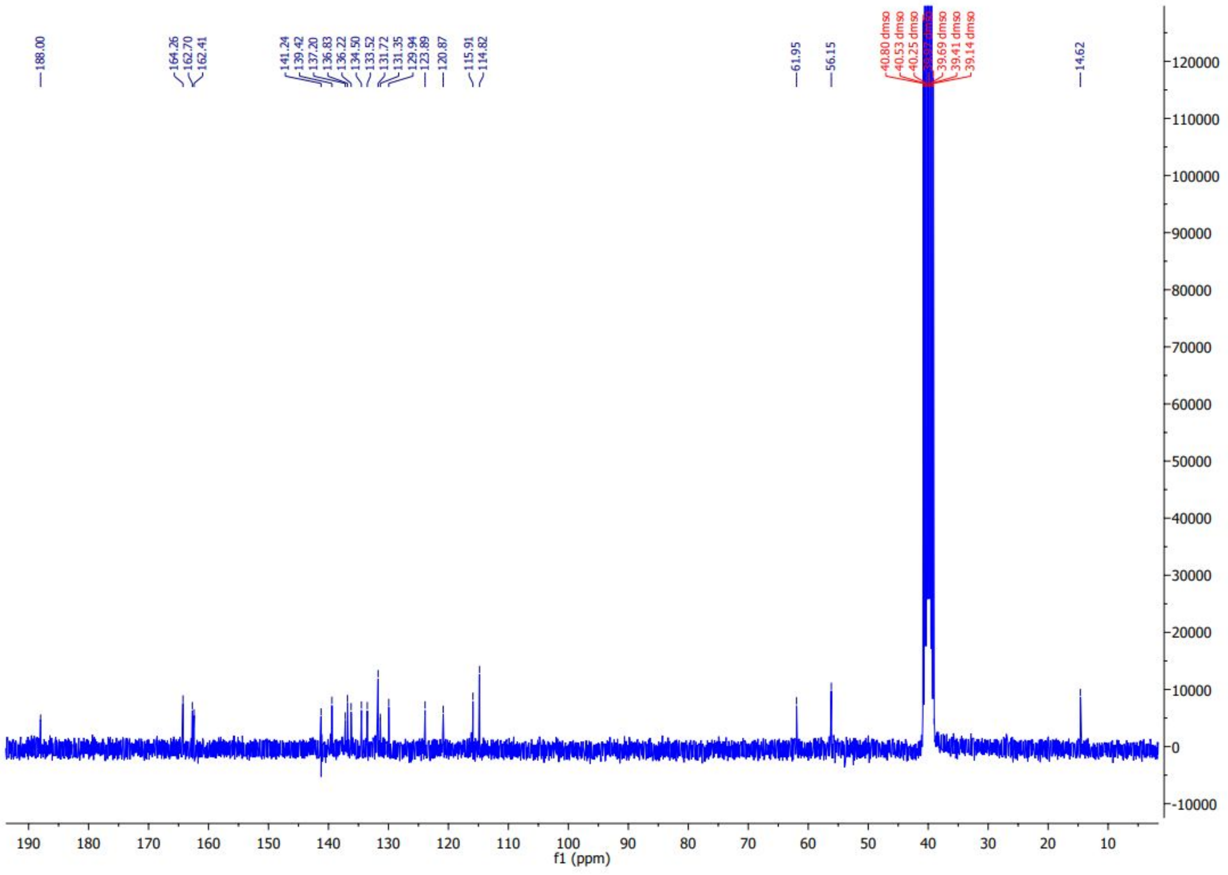

188.00
164.26
162.70
162.41
141.24
139.42
137.20
136.83
136.22
134.50
133.52
131.72
131.35
129.94
123.89
120.87
115.91
114.82
61.95
56.15
40.80 dmso
40.53 dmso
40.25 dmso
39.69 dmso
39.41 dmso
39.14 dmso
14.62
f1 (ppm)

Figure S32. Characterization of MG-HuR4

Analytical HPLC:

<Chromatogram>

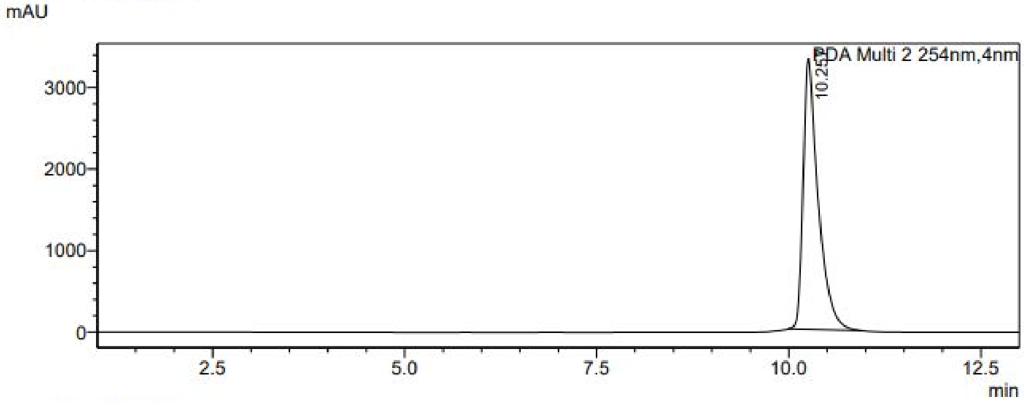

<Peak Table>

PDA Ch2 254nm

Peak#	Ret. Time	Area	Height	Area%
1	10.257	45012261	3317091	100.000
Total		45012261	3317091	100.000

NMR:

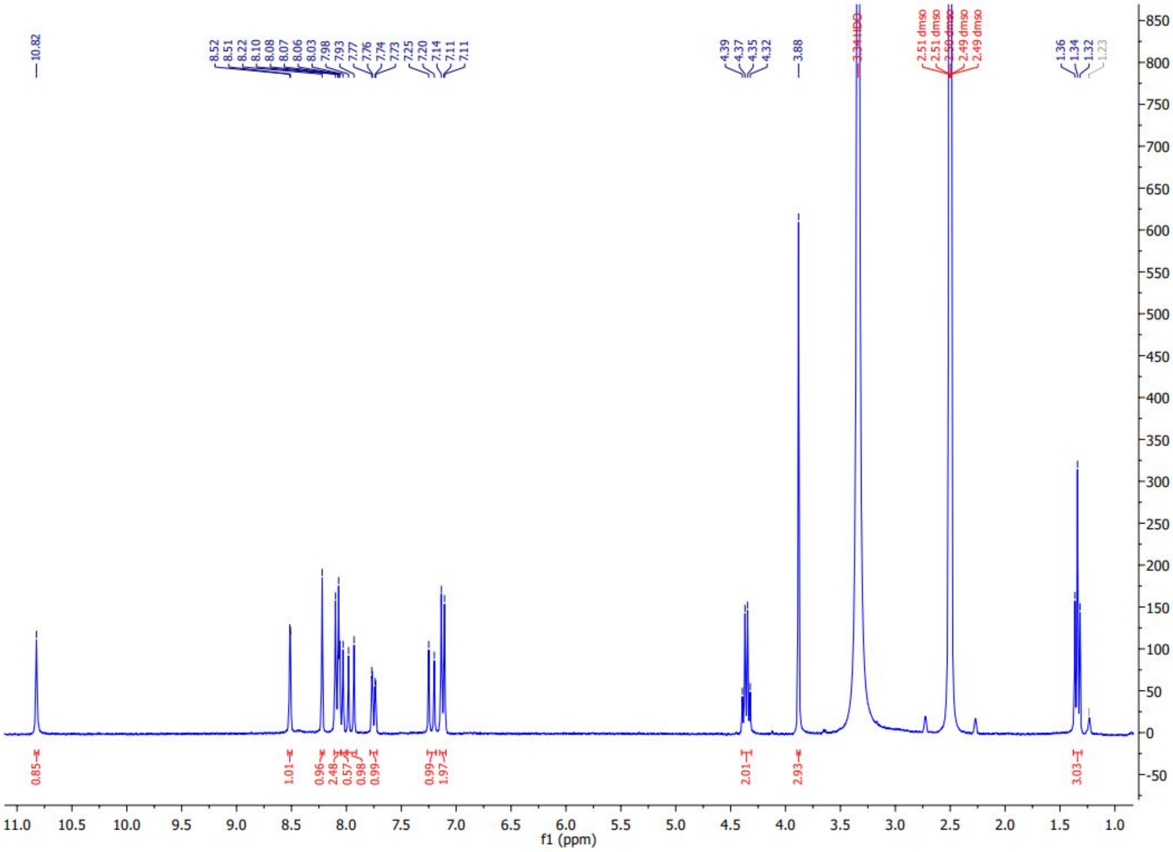

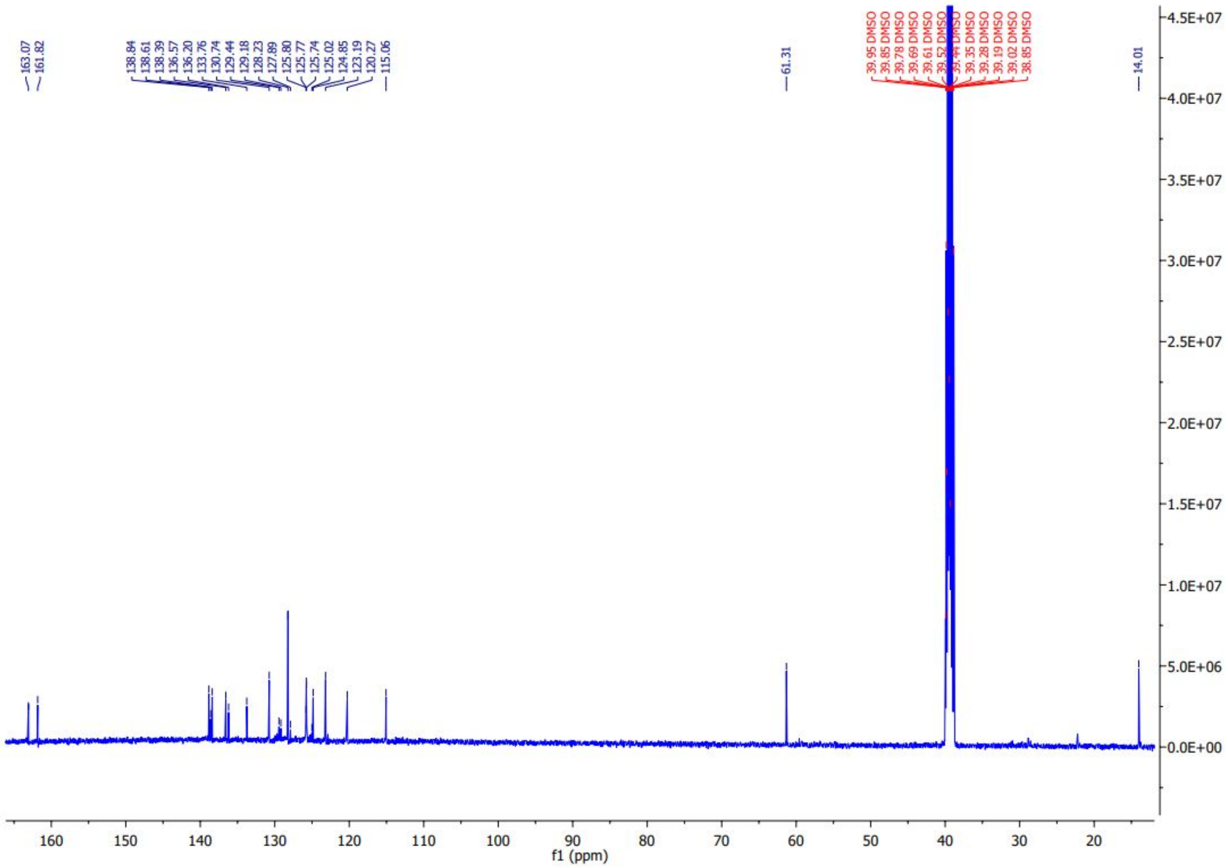
163.07
161.82
138.84
138.61
138.39
136.57
136.20
133.76
130.74
129.44
129.18
128.23
127.89
125.80
125.77
125.74
125.02
124.85
123.19
120.27
115.06
61.31
39.95 DMSO
39.85 DMSO
39.78 DMSO
39.69 DMSO
39.61 DMSO
39.52 DMSO
39.44 DMSO
39.35 DMSO
39.28 DMSO
39.19 DMSO
39.02 DMSO
38.85 DMSO
14.01
f1 (ppm)

Figure S33. Characterization of MG-HuR5

Analytical HPLC:

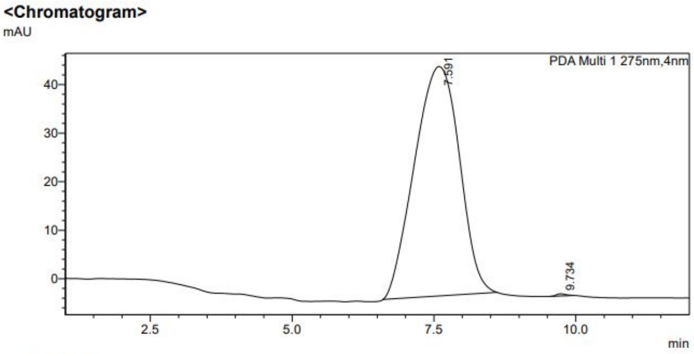

<Peak Table>

PDA Ch1 275nm

Peak#	Ret. Time	Area	Height	Area%
1	7.591	2612077	47271	99.796
2	9.734	5327	447	0.204
Total		2617403	47718	100.000

NMR:

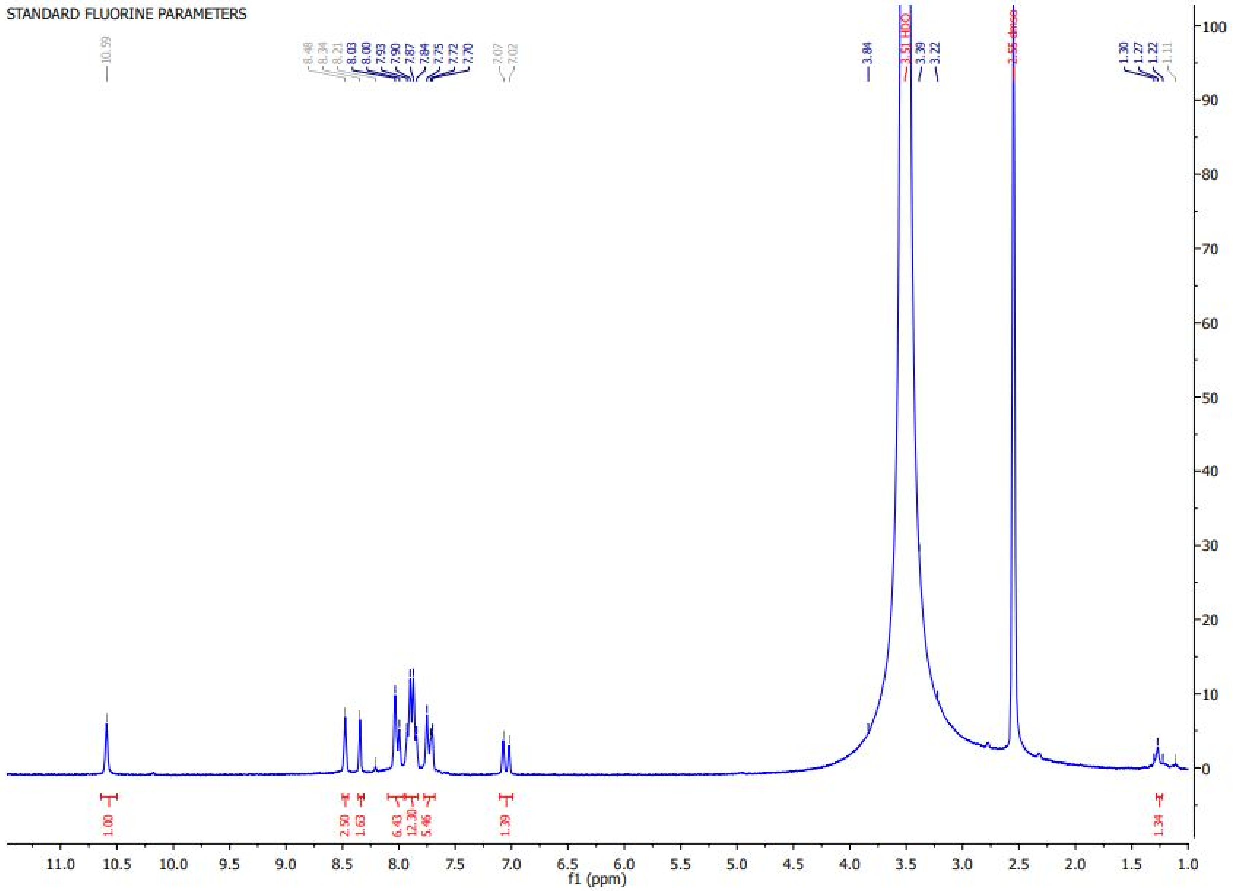

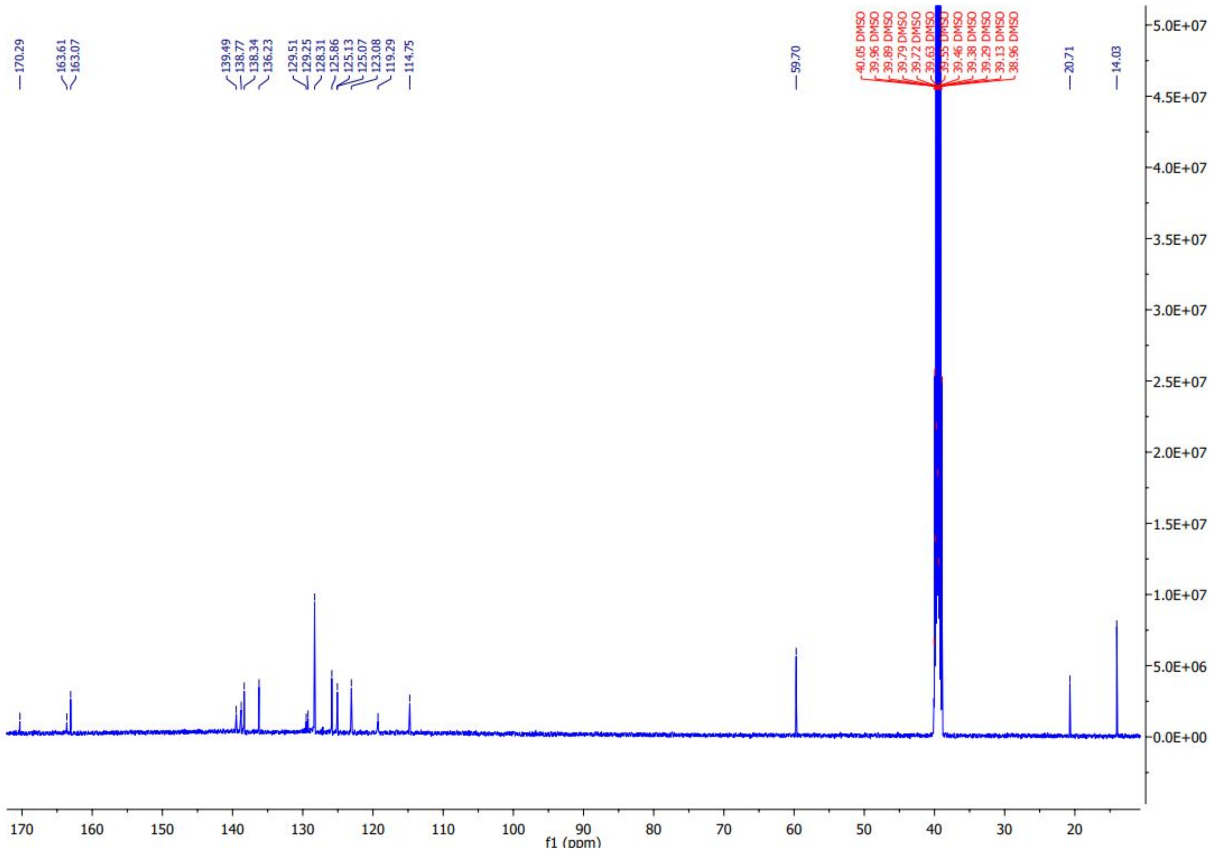
170.29
163.61
163.07
139.49
138.77
138.34
136.23
129.51
129.25
128.31
125.86
125.13
125.07
123.08
119.29
114.75
59.70
40.05 DMSO
39.96 DMSO
39.89 DMSO
39.79 DMSO
39.72 DMSO
39.63 DMSO
39.55 DMSO
39.46 DMSO
39.38 DMSO
39.29 DMSO
39.13 DMSO
38.96 DMSO
20.71
14.03
5.0E+07
4.5E+07
4.0E+07
3.5E+07
3.0E+07
2.5E+07
2.0E+07
1.5E+07
1.0E+07
5.0E+06
0.0E+00
170
160
150
140
130
120
110
100
90
80
70
60
50
40
30
20
f1 (ppm)

Figure S34. Characterization of PRO-HuR3

Analytical HPLC:

<Chromatogram>

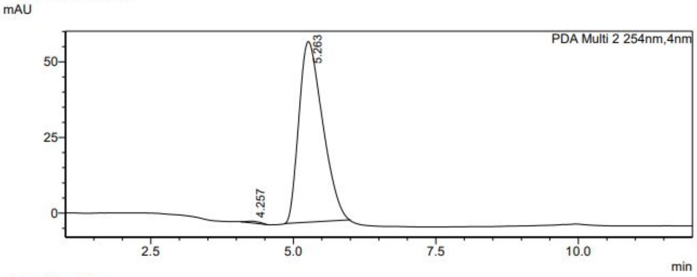

<Peak Table>

PDA Ch2 254nm

Peak#	Ret. Time	Area	Height	Area%
1	4.257	9387	515	0.538
2	5.263	1734029	59686	99.462
Total		1743415	60201	100.000

NMR:

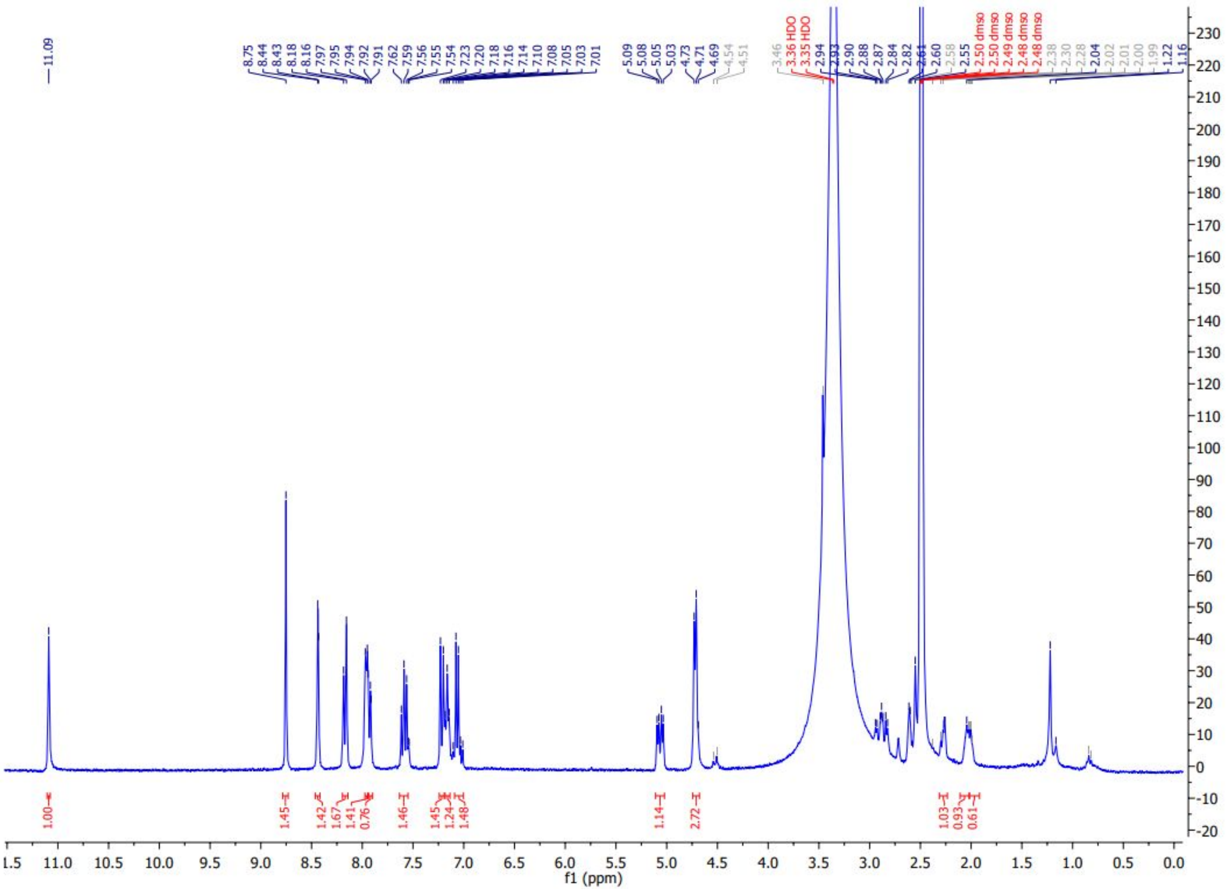

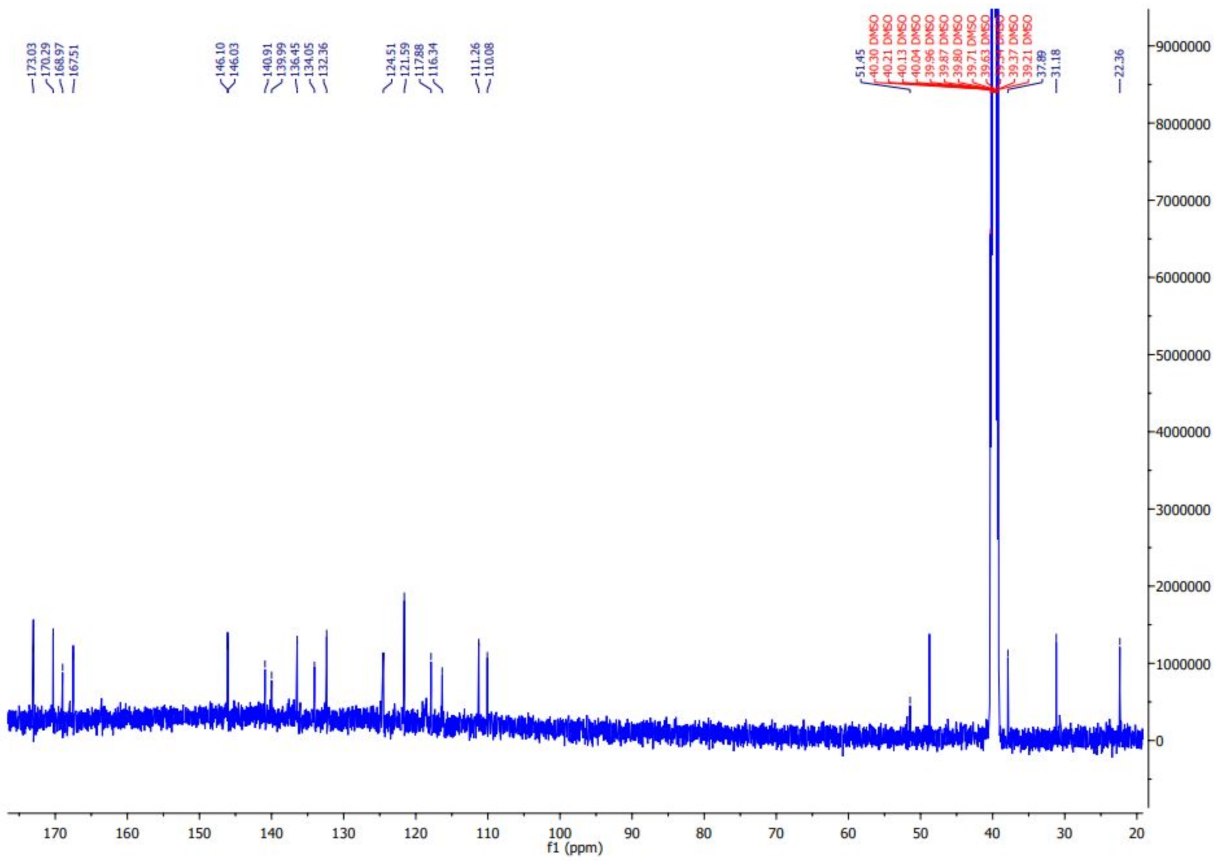

173.03
170.29
168.97
167.51
146.10
146.03
140.91
139.99
136.45
134.05
132.36
124.51
121.59
117.88
116.34
111.26
110.08
51.45
40.30 DMSO
40.21 DMSO
40.13 DMSO
40.04 DMSO
39.96 DMSO
39.87 DMSO
39.80 DMSO
39.71 DMSO
39.63 DMSO
39.37 DMSO
39.21 DMSO
37.89
31.18
22.36
9000000
8000000
7000000
6000000
5000000
4000000
3000000
2000000
1000000
0
170
160
150
140
130
120
110
100
90
80
70
60
50
40
30
20
f1 (ppm)

Figure S35. Characterization of PRO-HuR4

Analytical HPLC:

<Chromatogram>

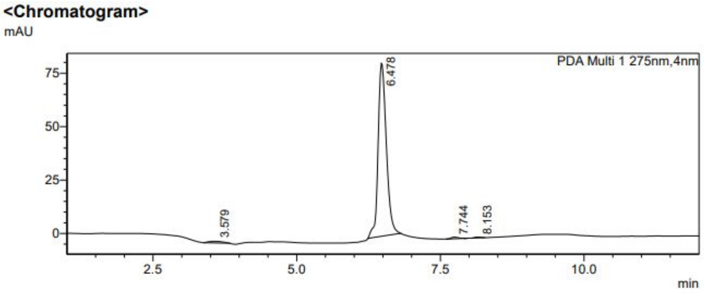

<Peak Table>

PDA Ch1 275nm

Peak#	Ret. Time	Area	Height	Area%
1	3.579	13318	794	1.600
2	6.478	809980	80813	97.298
3	7.744	6649	725	0.799
4	8.153	2528	357	0.304
Total		832475	82689	100.000

NMR:

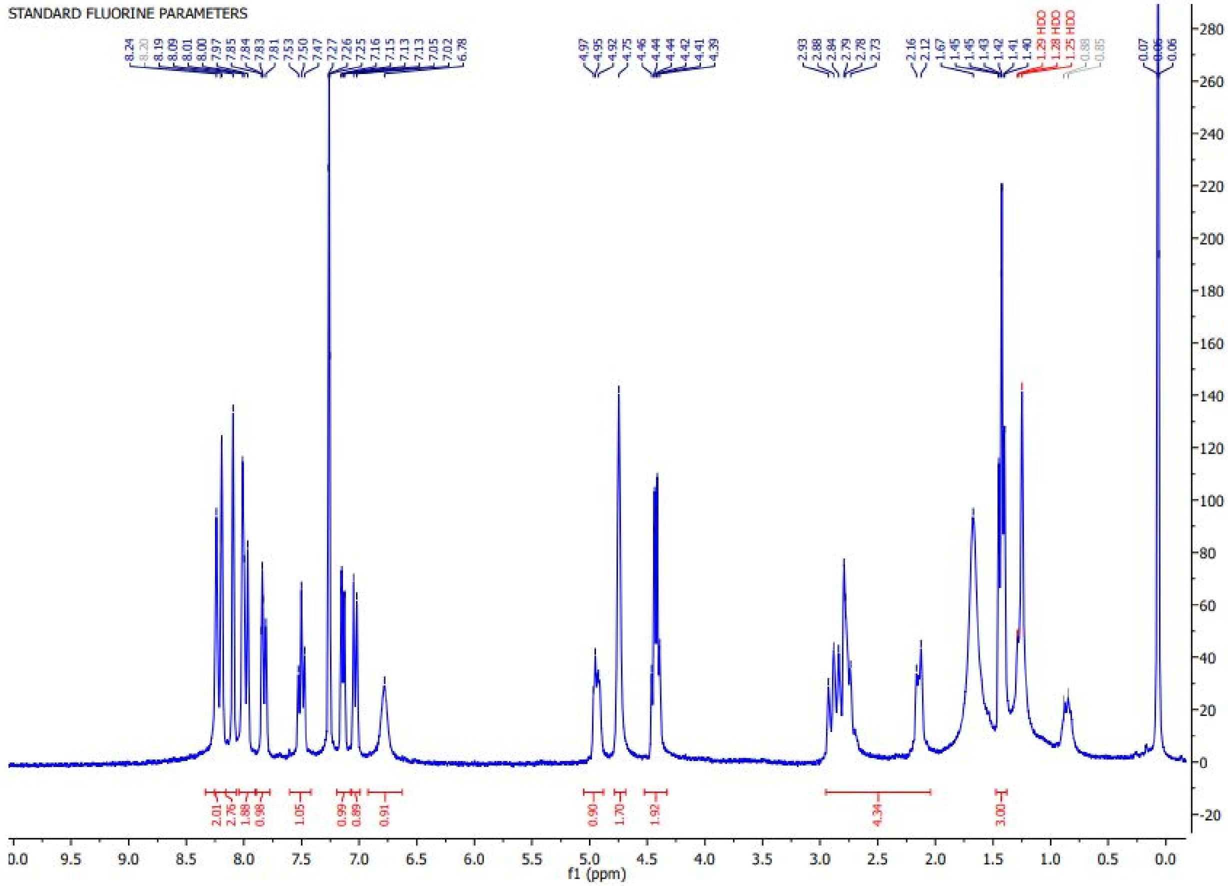

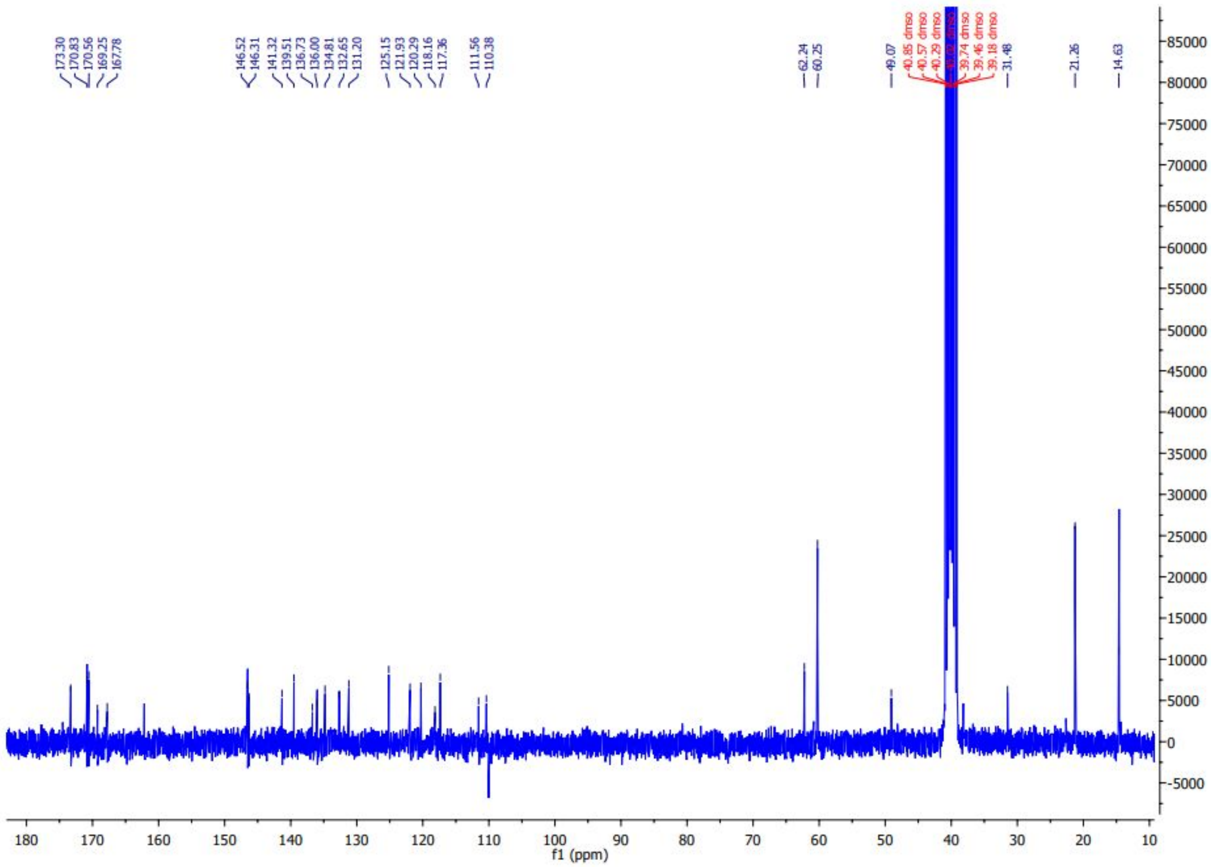

173.30
170.83
170.56
169.25
167.78
146.52
146.31
141.32
139.51
136.73
136.00
134.81
132.65
131.20
125.15
121.93
120.29
118.16
117.36
111.56
110.38
62.24
60.25
49.07
40.85 dmso
40.57 dmso
40.29 dmso
39.74 dmso
39.46 dmso
39.18 dmso
31.48
21.26
14.63
f1 (ppm)

Figure S36. Characterization of PRO-HuR5

Analytical HPLC:

<Chromatogram>

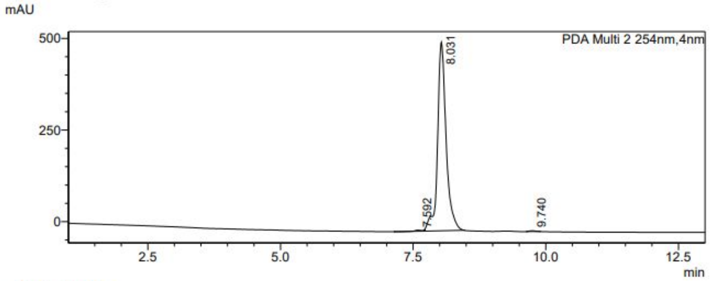

<Peak Table>

PDA Ch2 254nm

Peak#	Ret. Time	Area	Height	Area%
1	7.592	29382	2903	0.505
2	8.031	5774576	514180	99.207
3	9.740	16776	2051	0.288
Total		5820735	519133	100.000

NMR:

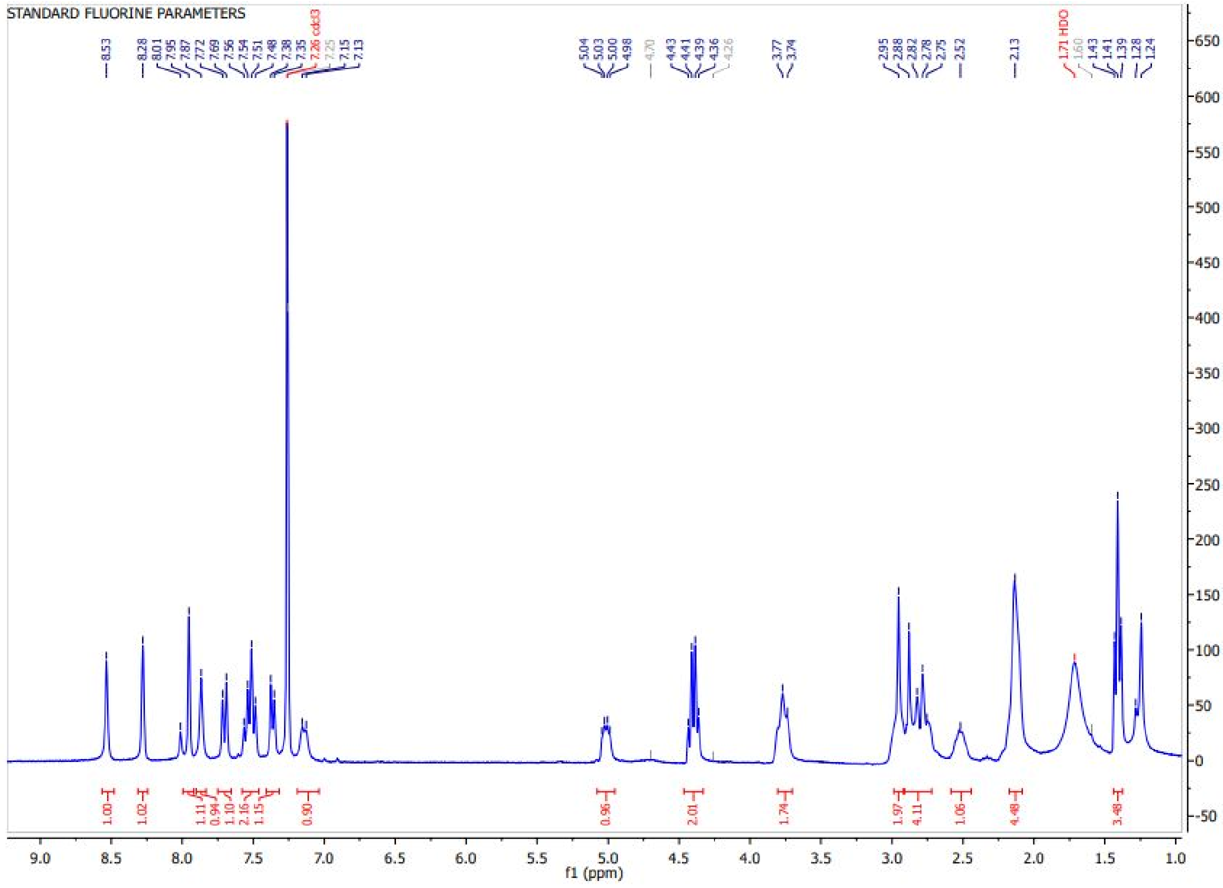

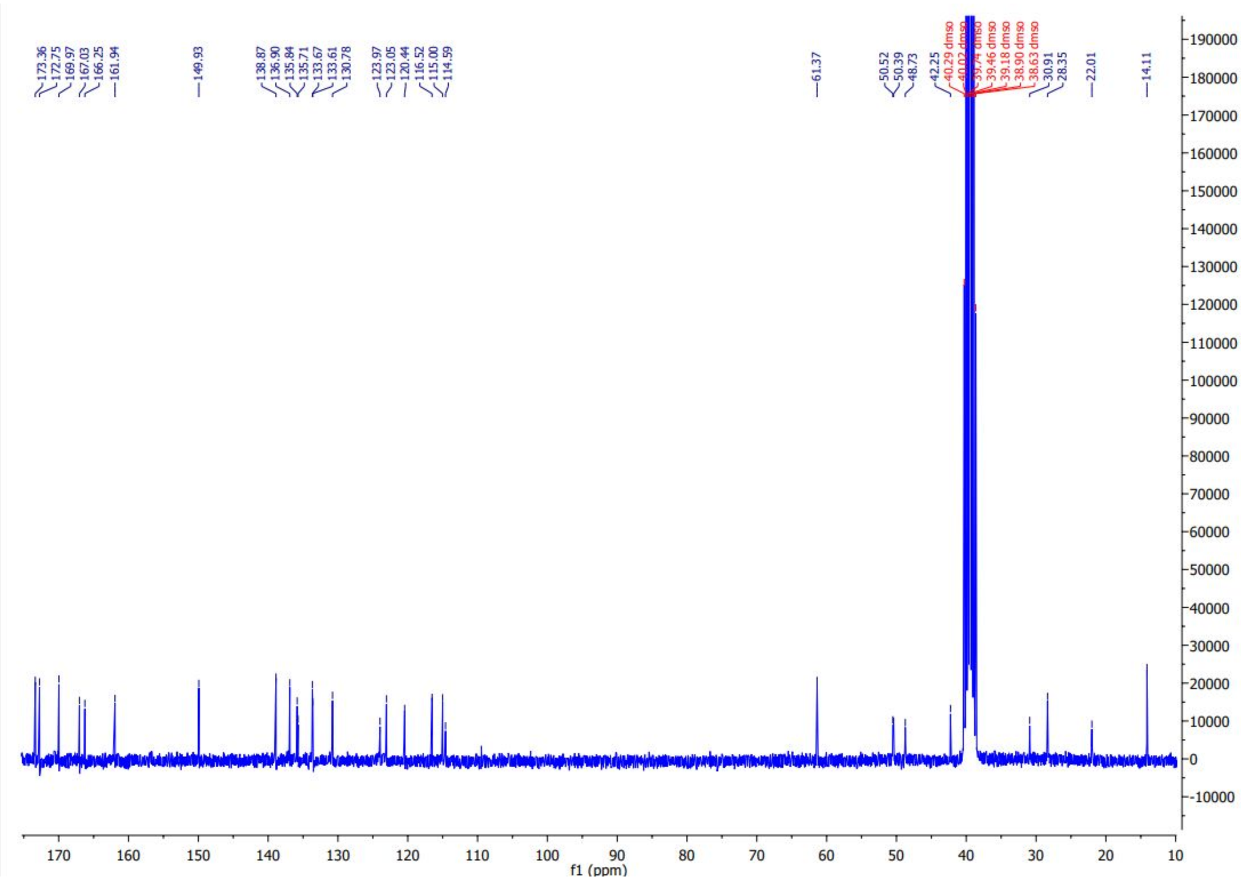
173.36
172.75
169.97
167.03
166.25
161.94
149.93
138.87
136.90
135.84
135.71
133.67
133.61
130.78
123.97
123.05
120.44
116.52
115.00
114.59
61.37
50.52
50.39
48.73
42.25
40.29 dmso
40.02 dmso
39.74 dmso
39.46 dmso
39.18 dmso
38.90 dmso
38.63 dmso
30.91
28.35
22.01
14.11
f1 (ppm)

Figure S37. Characterization of PRO-HuR6

Analytical HPLC:

<Chromatogram>

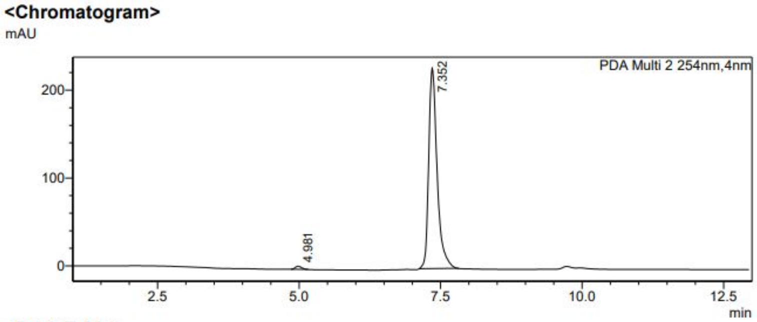

<Peak Table>

PDA Ch2 254nm

Peak#	Ret. Time	Area	Height	Area%
1	4.981	26356	3452	1.102
2	7.352	2365218	227752	98.898
Total		2391574	231204	100.000

NMR:

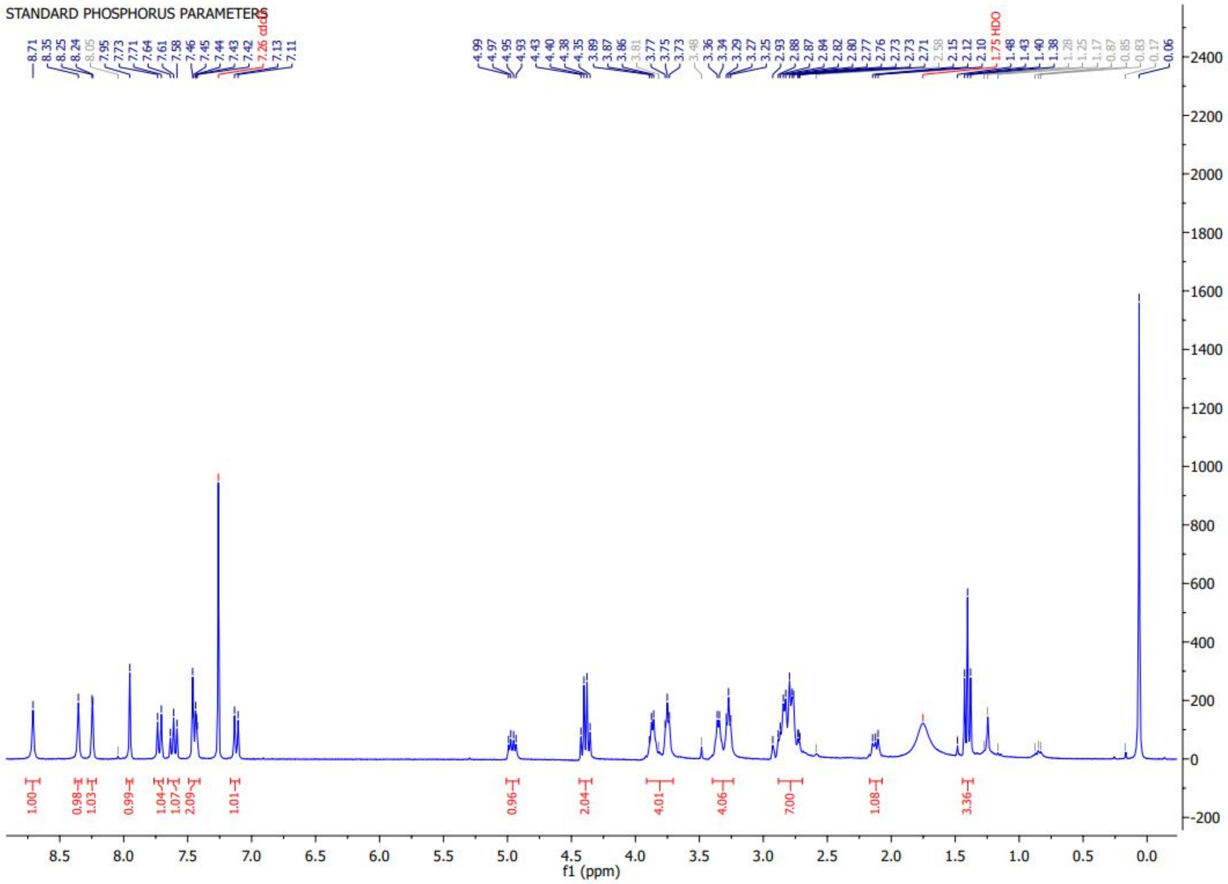

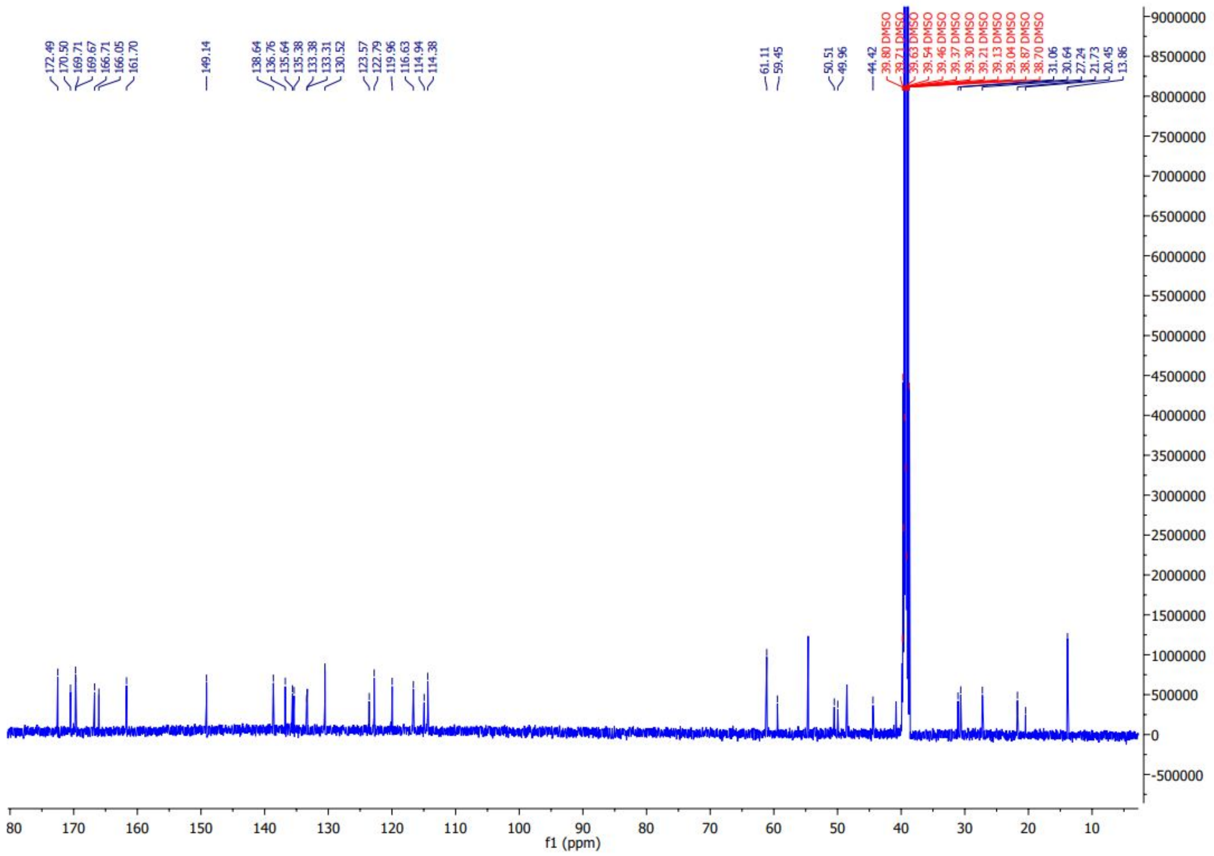
172.49
170.50
169.71
169.67
166.71
166.05
161.70
149.14
138.64
136.76
135.64
135.38
133.38
133.31
130.52
123.57
122.79
119.96
116.63
114.94
114.38
61.11
59.45
50.51
49.96
44.42
39.80 DMSO
39.71 DMSO
39.63 DMSO
39.54 DMSO
39.46 DMSO
39.37 DMSO
39.30 DMSO
39.21 DMSO
39.13 DMSO
39.04 DMSO
38.87 DMSO
38.70 DMSO
31.06
30.64
27.24
21.73
20.45
13.86
9000000
8500000
8000000
7500000
7000000
6500000
6000000
5500000
5000000
4500000
4000000
3500000
3000000
2500000
2000000
1500000
1000000
500000
0
-500000
80 170 160 150 140 130 120 110 100 90 80 70 60 50 40 30 20 10
f1 (ppm)

Figure S38. Characterization of PRO-HuR7

Analytical HPLC:

<Chromatogram>

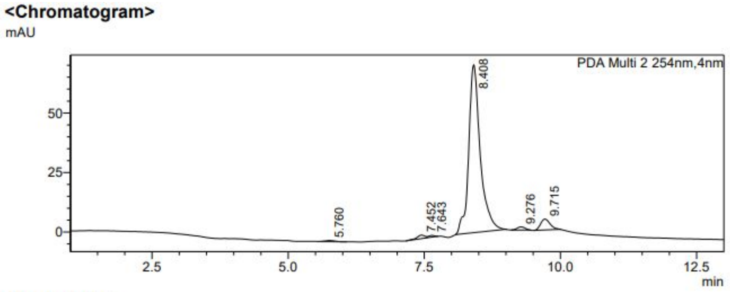

<Peak Table>

PDA Ch2 254nm

Peak#	Ret. Time	Area	Height	Area%
1	5.760	5804	455	0.512
2	7.452	15200	1412	1.342
3	7.643	5009	644	0.442
4	8.408	1038460	70392	91.679
5	9.276	13213	1311	1.166
6	9.715	55030	4536	4.858
Total		1132716	78750	100.000

NMR:

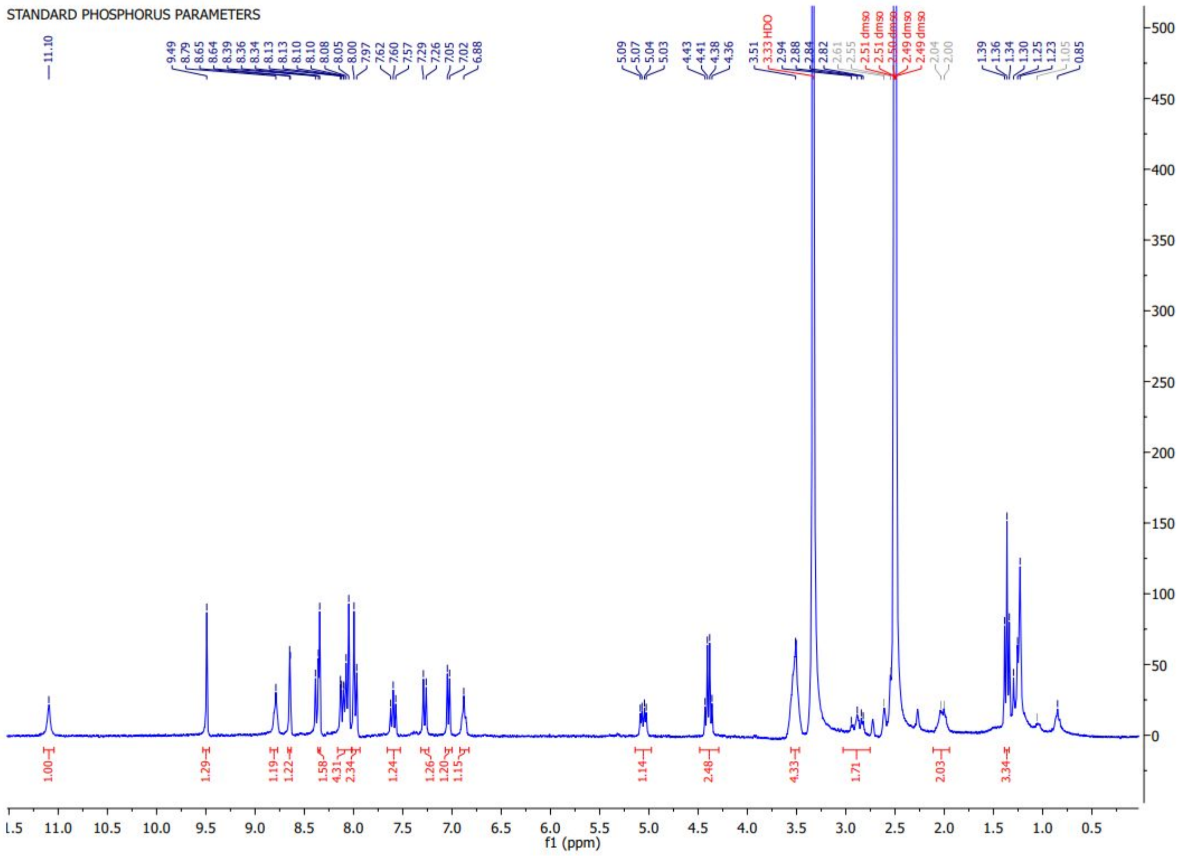

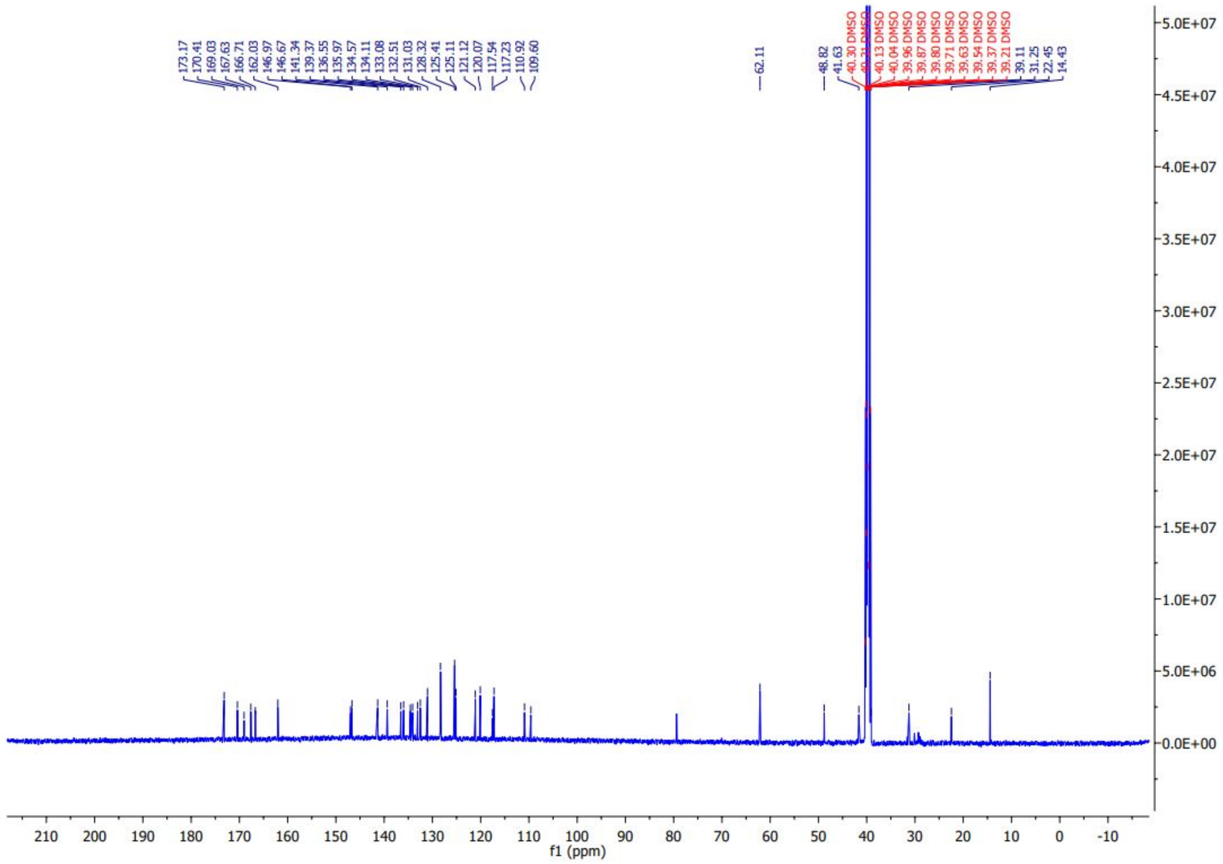
173.17
170.41
169.03
167.63
166.71
162.03
146.97
146.67
141.34
139.37
136.55
135.97
134.57
134.11
133.08
132.51
131.03
128.32
125.41
125.11
121.12
120.07
117.54
117.23
110.92
109.60
62.11
48.82
41.63
40.30 DMSO
40.13 DMSO
40.04 DMSO
39.96 DMSO
39.87 DMSO
39.80 DMSO
39.71 DMSO
39.63 DMSO
39.54 DMSO
39.37 DMSO
39.21 DMSO
39.11
31.25
22.45
14.43
f1 (ppm)

Figure S39. Characterization of PRO-HuR8

Analytical HPLC:

<Chromatogram>

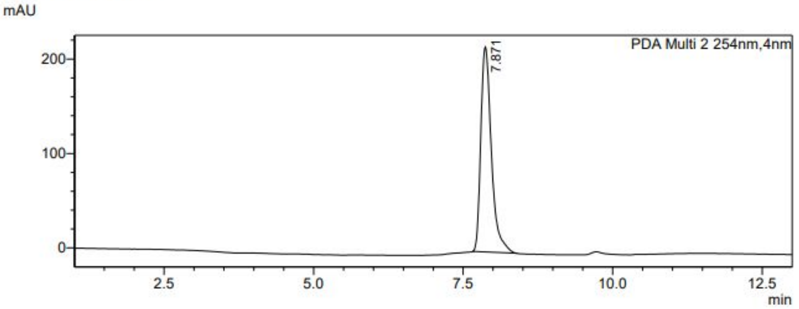

<Peak Table>

PDA Ch2 254nm

Peak#	Ret. Time	Area	Height	Area%
1	7.871	2673531	217087	100.000
Total		2673531	217087	100.000

NMR:

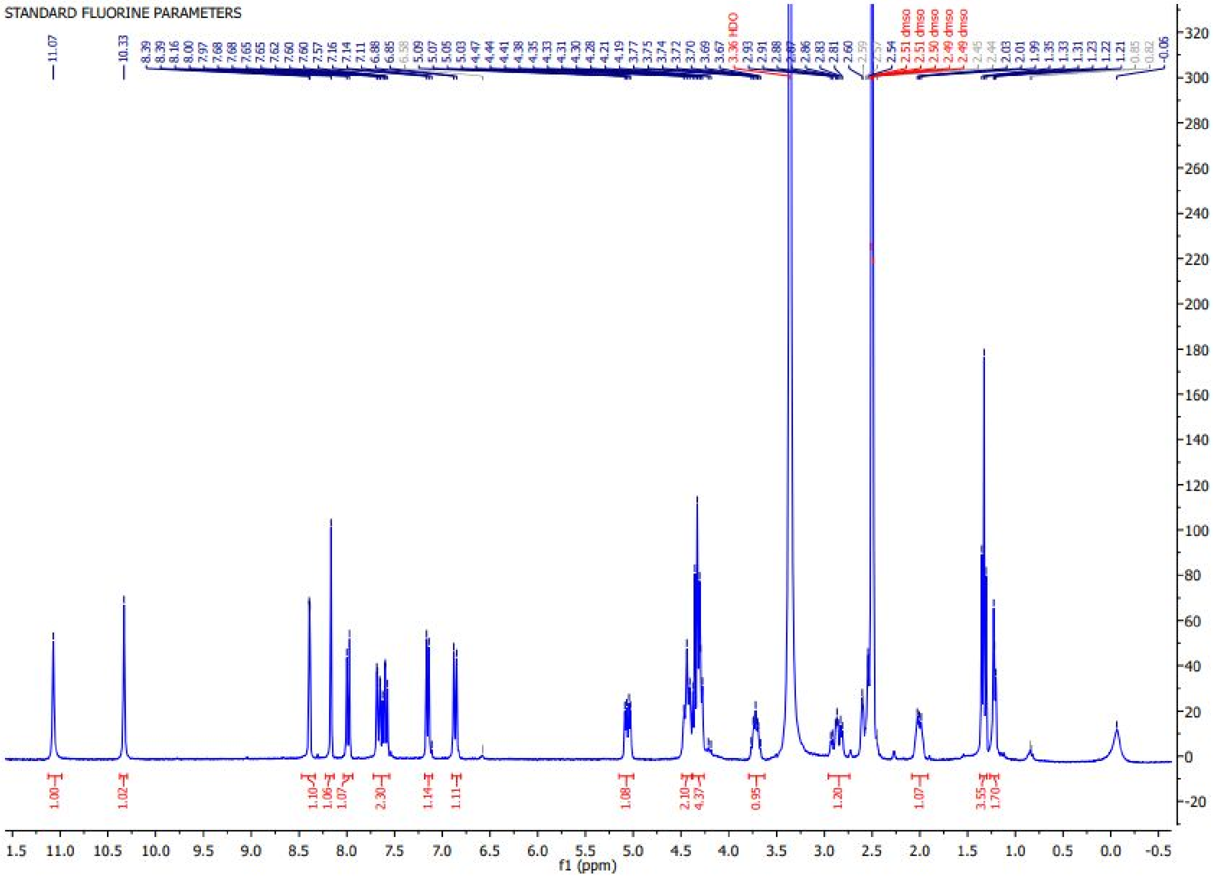

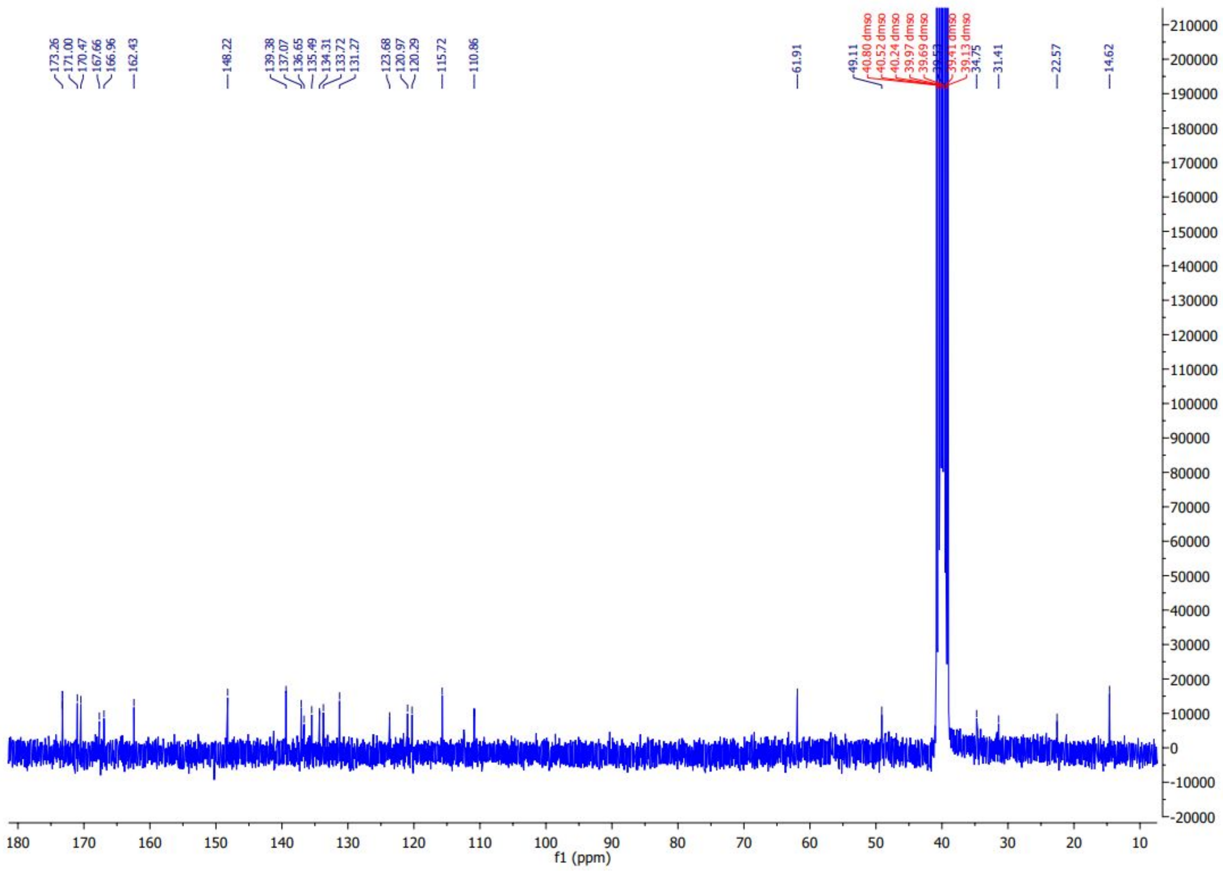

173.26
171.00
170.47
167.66
166.96
162.43
148.22
139.38
137.07
136.65
135.49
134.31
133.72
131.27
123.68
120.97
120.29
115.72
110.86
61.91
49.11
40.80 dmso
40.52 dmso
40.24 dmso
39.97 dmso
39.69 dmso
39.41 dmso
39.13 dmso
34.75
31.41
22.57
14.62
f1 (ppm)

5. References

(1) David Pierson, P.; Fettes, A.; Freichel, C.; Gatti-Mac Arthur, S.; Hertel, C.; Huwyler, J.; Mohr, P.; Nakagawa, T.; Nettekoven, M.; Plancher, J.-M.; Raab, S.; Richter, H.; Roche, O.; María Rodríguez Sarmiento, R.; Schmitt, M.; Schuler, F.; Takahashi, T.; Taylor, S.; Ullmer, C.; Wiegand, R. SUPPORTING INFORMATION 5-Hydroxyindole-2-Carboxylic Acid Amides: Novel Histamine-3 Receptor Inverse Agonists for the Treatment of Obesity. *J Med Chem* **2009**, *52* (13), 855–3868. https://doi.org/https://doi.org/10.1021/jm900409x.

(2) Ambrose, A. J.; Sivinski, J.; Zerio, C. J.; Zhu, X.; Godek, J.; Kumirov, V. K.; Coma Brujas, T.; Torra Garcia, J.; Annadurai, A.; Schmidlin, C. J.; Werner, A.; Shi, T.; Zavareh, R. B.; Lairson, L.; Zhang, D. D.; Chapman, E. Discovery and Development of a Selective Inhibitor of the ER Resident Chaperone Grp78. *J Med Chem* **2023**, *66* (1), 677–694. https://doi.org/10.1021/acs.jmedchem.2c01631.

(3) Nowak, R. P.; Deangelo, S. L.; Buckley, D.; He, Z.; Donovan, K. A.; An, J.; Safaee, N.; Jedrychowski, M. P.; Ponthier, C. M.; Ishoey, M.; Zhang, T.; Mancias, J. D.; Gray, N. S.; Bradner, J. E.; Fischer, E. S. Plasticity in Binding Confers Selectivity in Ligand-Induced Protein Degradation Article. *Nat Chem Biol* **2018**, *14* (7), 706–714. https://doi.org/10.1038/s41589-018-0055-y.

(4) Gnaccarini, C.; Ben-Tahar, W.; Mulani, A.; Roy, I.; Lubell, W. D.; Pelletier, J. N.; Keillor, J. W. Site-Specific Protein Propargylation Using Tissue Transglutaminase. *Org Biomol Chem* **2012**, *10* (27), 5258–5265. https://doi.org/10.1039/c2ob25752a.

(5) Patil, K. M.; Chin, D.; Seah, H. L.; Shi, Q.; Lim, K. W.; Phan, A. T. G4-PROTAC: Targeted Degradation of a G-Quadruplex Binding Protein. *Chemical Communications* **2021**, *57* (95), 12816–12819. https://doi.org/10.1039/d1cc05025g.

(6) Van Snick, W.; Nulens, W.; Jambon, S.; Dehaen, W. A Facile Synthetic Route towards Substituted Thieno[3,2-e]Indoles. *Synthesis (Stuttg)* **2009**, No. 5, 767–774. https://doi.org/10.1055/s-0028-1083366.

(7) Fieser, L. F.; Kennelly, R. G. HI). *obshch. i m D.I. Mendeleeva 15*, 57–1611. https://doi.org/10.1039/P19740000575.

(8) Fang, X.; Zhang, T.; Fang, W.; Zhang, G.; Li, Y.; Li, Y. Synthesis of Functionalized Triazoles on DNA via Azide-Acetonitrile “Click” Reaction. *Org Lett* **2023**, *25* (46), 8326–8331. https://doi.org/10.1021/acs.orglett.3c03404.

(9) Zhou, F.; Chen, L.; Cao, C.; Yu, J.; Luo, X.; Zhou, P.; Zhao, L.; Du, W.; Cheng, J.; Xie, Y.; Chen, Y. Development of Selective Mono or Dual PROTAC Degrader Probe of CDK Isoforms. *Eur J Med Chem* **2020**, *187*. https://doi.org/10.1016/j.ejmech.2019.111952.

(10) Chen, Z.; Wang, M.; Wu, D.; Zhao, L.; Metwally, H.; Jiang, W.; Wang, Y.; Bai, L.; McEachern, D.; Luo, J.; Wang, M.; Li, Q.; Matvekas, A.; Wen, B.; Sun, D.; Chinnaiyan, A. M.; Wang, S. Discovery of CBPD-409 as a Highly Potent, Selective, and Orally Efficacious CBP/P300 PROTAC Degrader for the Treatment of Advanced Prostate Cancer. *J Med Chem* **2024**, *67* (7), 5351–5372. https://doi.org/10.1021/acs.jmedchem.3c01789.

(11) Qiu, X.; Sun, N.; Kong, Y.; Li, Y.; Yang, X.; Jiang, B. Chemoselective Synthesis of Lenalidomide-Based PROTAC Library Using Alkylation Reaction. *Org Lett* **2019**, *21* (10), 3838–3841. https://doi.org/10.1021/acs.orglett.9b01326.
